# Genome comparisons reveal accessory genes crucial for the evolution of apple Glomerella leaf spot pathogenicity in *Colletotrichum* fungi

**DOI:** 10.1111/mpp.13454

**Published:** 2024-04-15

**Authors:** Xiaofei Liang, Wei Yu, Yanan Meng, Shengping Shang, Huanhuan Tian, Zhaohui Zhang, Jeffrey A. Rollins, Rong Zhang, Guangyu Sun

**Affiliations:** ^1^ State Key Laboratory of Crop Stress Biology in Arid Areas College of Plant Protection, Northwest A&F University Yangling China; ^2^ Department of Plant Pathology University of Florida Gainesville Florida USA

**Keywords:** ascomycetes, *Colletotrichum*, genome comparison, long‐read sequencing, *Malus domestica*, secondary metabolite, virulence evolution

## Abstract

Apple Glomerella leaf spot (GLS) is an emerging fungal disease caused by *Colletotrichum fructicola* and other *Colletotrichum* species. These species are polyphyletic and it is currently unknown how these pathogens convergently evolved to infect apple. We generated chromosome‐level genome assemblies of a GLS‐adapted isolate and a non‐adapted isolate in *C. fructicola* using long‐read sequencing. Additionally, we resequenced 17 *C. fructicola* and *C. aenigma* isolates varying in GLS pathogenicity using short‐read sequencing. Genome comparisons revealed a conserved bipartite genome architecture involving minichromosomes (accessory chromosomes) shared by *C. fructicola* and other closely related species within the *C. gloeosporioides* species complex. Moreover, two repeat‐rich genomic regions (1.61 Mb in total) were specifically conserved among GLS‐pathogenic isolates in *C. fructicola* and *C. aenigma*. Single‐gene deletion of 10 accessory genes within the GLS‐specific regions of *C. fructicola* identified three that were essential for GLS pathogenicity. These genes encoded a putative non‐ribosomal peptide synthetase, a flavin‐binding monooxygenase and a small protein with unknown function. These results highlight the crucial role accessory genes play in the evolution of *Colletotrichum* pathogenicity and imply the significance of an unidentified secondary metabolite in GLS pathogenesis.

## Graphical abstract

Apple Glomerella leaf spot isolates in *Colletotrichum fructicola* contain pathogenicity‐associated accessory genes that suppress plant defences by synthesizing an unidentified secondary metabolite.

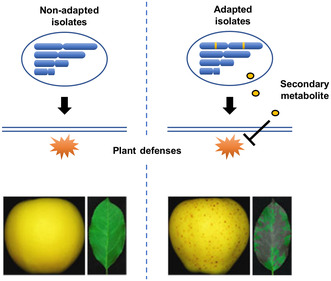

## 1 INTRODUCTION

The genus *Colletotrichum* (Glomerellales, Ascomycota) is one of the most important groups of fungal plant pathogens (Dean et al., 2012). It contains over 200 species belonging to at least 16 species complexes (Bhunjun et al., 2021; Liu et al., 2022). The genus collectively infects nearly 3000 plant species and causes significant harm to many valuable fruits, vegetables, ornamentals and cereals (Cannon et al., 2012; O'Connell et al., 2012). On apple (*Malus domestica*), *Colletotrichum* species cause two significant diseases, apple bitter rot (ABR) and Glomerella leaf spot (GLS) (Chen et al., 2022; Rockenbach et al., 2016; Velho et al., 2015). ABR is a long‐standing disease worldwide that has been documented since the 1800s (Alwood, 1890; Brook, 1977). It causes extensive watery rot on near‐mature or in‐storage fruits. GLS is a foliar disease that damages young leaves by producing irregular necrotic lesions and defoliation. GLS also damages apple fruits by producing small sunken lesions that, however, do not progress into water‐soaking rot. GLS was initially described in the 1970s (Taylor, 1971) and is currently only found in the United States, Brazil, China and Uruguay (Alaniz et al., 2019; Chen et al., 2022; Gonzalez et al., 2006; Hamada et al., 2019). In the field, all apple cultivars are susceptible to ABR, but only those in the Golden Delicious group, specifically Gala, are susceptible to GLS (Liu et al., 2016; Rockenbach et al., 2016).

The differences in symptoms, history of occurrence and host cultivar and tissue specificity between GLS and ABR diseases suggest distinct pathogenic mechanisms. Pathogens that cause GLS and ABR are both taxonomically diverse and polyphyletic (Chen et al., 2022; Liang et al., 2022). There have been reports of nine distinct species belonging to *Colletotrichum gloeosporioides* species complex (CGSC), *Colletotrichum acutatum* species complex (CASC) and *Colletotrichum boninense* species complex (CBSC) capable of causing GLS. Meanwhile, more than 10 ABR species belonging to the CGSC and the CASC have been reported. Interestingly, certain *Colletotrichum* species (e.g., *C. fructicola*) contain specialized isolates capable of causing either GLS or ABR (Rockenbach et al., 2016). Such intraspecific pathogenic differentiation and polyphyletic distribution of GLS pathogen species indicate multiple origins of GLS pathogenicity, which may have their origins in horizontal transfer of a pathogenicity determinant(s). While comparisons between GLS and ABR isolates have been made in terms of hydrolytic enzymatic activities (Velho et al., 2018) and global transcriptomes (Jiang et al., 2022), genetic processes underpinning the evolutionary origin of GLS pathogenicity remain mostly unclear.

Filamentous plant pathogens, including phylogenetically unrelated fungi and oomycetes, interact antagonistically with their plant hosts, creating a co‐evolutionary arms race between pathogenicity and defence. For these pathogens, genome compartmentalization is a key host adaptation and virulence‐related genes are more likely to be found in rapidly evolving genomic regions such as gene‐sparse and repeat‐rich regions, AT‐rich isochores or accessory chromosomes (Dong et al., 2015; Frantzeskakis et al., 2019). Accessory genes located within these regions promote pathogen infection by acting as phytoalexin‐detoxifying enzymes, plant defence suppressors, host‐selective toxins and other defined and undefined functions (Bertazzoni et al., 2018). Over evolutionary time, accessory genes also promote the expansion of pathogenicity towards new plant varieties or species, posing a continual threat to crop production (Bertazzoni et al., 2018).

In order to analyse genetic factor(s) associated with GLS pathogenicity, we generated chromosome‐level genome assemblies of two *C. fructicola* isolates, 1104‐7 and LJ19, derived from apple GLS lesion and bell pepper (*Capsicum annuum*) fruit, respectively. Artificial inoculation showed that 1104‐7 is GLS pathogenic, whereas LJ19 is GLS nonpathogenic. We performed genome comparison between the two isolates and among closely related species belonging to CGSC. We found that *C. fructicola* and other CGSC species share a conserved bipartite genome architecture pattern, with two short minichromosomes exhibiting high inter‐ and intraspecific variation. Furthermore, using 17 additional *C. fructicola* and *C. aenigma* isolates derived from different plant hosts and being either pathogenic or nonpathogenic for GLS, we performed genome resequencing analysis and identified two genomic regions that are GLS specific. Through deletion analysis with 10 accessory genes within these two GLS‐specific regions in *C. fructicola*, we identified three that are important for GLS pathogenicity. The results of our study shed light on virulence mechanisms and evolution in *Colletotrichum* fungi and emphasize the significance of accessory genes in controlling apple GLS pathogenicity.

## 2 RESULTS

### 2.1 Near‐complete genome assemblies of two *C. fructicola* isolates

**FIGURE 1:**
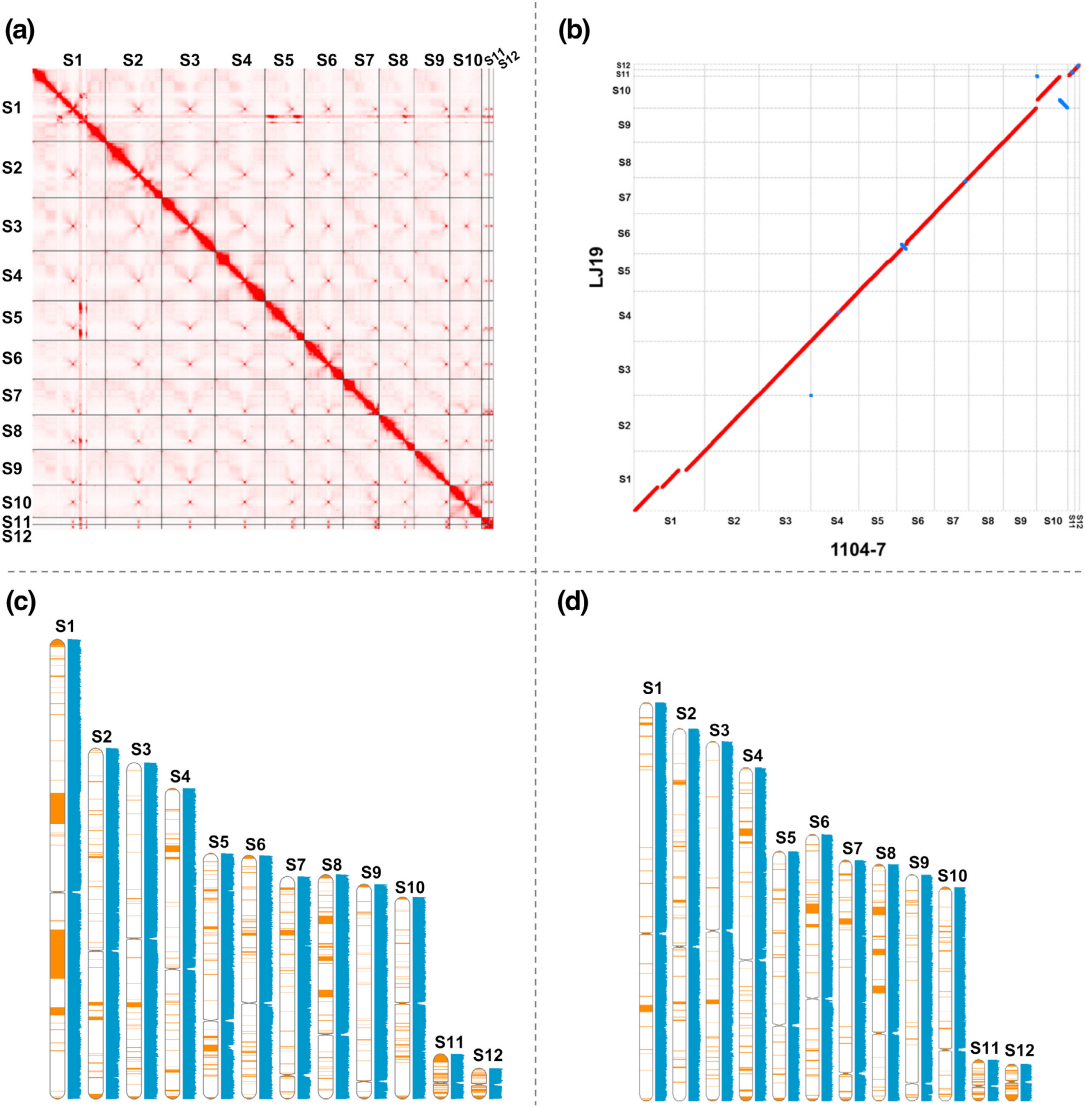
Assembly of *Colletotrichum fructicola* 1104‐7 and LJ19 genomes from nanopore long reads. (a) Genome‐wide Hi‐C contact map of 1104‐7. Putative centromeres are indicated by cross‐like patterns. (b) Dot plot showing the genome alignment between 1104‐7 and LJ19. Matches were identified using nucmer in Mummer, forward matches are in red and reverse matches are in blue, only highly similar matches (DNA identity >99%, length >10 kb) are shown. (c) 1104‐7 genome queried through BlastN with LJ19. The 1104‐7 genomic regions lacking matches in LJ19 are highlighted in brown and slide window of 10 kb GC content is shown in blue. Matches were identified by BlastN search with a stringency cut‐off of DNA identity >99% and length >10 kb. Note the large GC content drop near putative centromeres. (d) Lineage‐specific regions of LJ19 genome queried through BlastN with 1104‐7.

Nanopore sequencing generated reads with total sizes of 7.56 Gb (N50 length = 27.87 kb) and 27.46 Gb (N50 length = 28.97 kb) for the GLS‐pathogenic isolate 1104‐7 and GLS‐nonpathogenic isolate LJ19, respectively. Using these reads, two genome assemblies were produced using NextDenovo, with total lengths of 58.55 and 55.69 Mb (Table S1). There were 12 scaffolds in each assembly. For 1104‐7, five scaffolds had copies of a telomeric repeat (TTAGGG) on both ends and three scaffolds had telomeric repeats on one end. Aligned reads at six additional scaffold ends extended outwards to reach telomeric repeats, supporting that these ends are nearly complete (Table S2). For LJ19, five scaffolds had repeats on both ends and six scaffolds had repeats on one end, and three additional scaffold ends were close to telomeric repeats. Previously generated Hi‐C reads for 1104‐7 (Liang et al., 2020) were used to validate the accuracy of the 1104‐7 assembly. In the Hi‐C contact map, the strongest signal was diagonal and each contig contained a putative centromere, indicating a correspondence relationship between acquired contig and chromosome (Figure 1a). A high degree of chromosome collinearity was seen between 1104‐7 and LJ19, with no interchromosomal translocation events detected (Figure 1b). In both assemblies, the 3′ end of scaffold S4 corresponded to ribosomal DNA repeats, whereas scaffolds S11 and S12 corresponded to putative minichromosomes (length <1 Mb). Using a rigorous criterion (DNA identity >99%, length >10 kb), 92.23% (52.29 Mb) of LJ19 genomic DNA could be aligned to the 1104‐7 reference genome (Figure 1c), whereas 89.30% (52.84 Mb) of 1104‐7 genomic DNA could be aligned to LJ19 using the same criterion (Figure 1d).

We previously reported a 1104‐7 genome assembly (Liang et al., 2020) using the same set of nanopore reads data as here, but using a different assembling workflow (a combination of Canu and Flye), which had 16 scaffolds in total. The sizes of the two assembly versions were similar (58.55 Mb vs. 58.69 Mb), and the variation in the number of scaffolds was caused by the combination of five scaffolds from the previous version (S4, S11, S14–S16) into one scaffold (S1) in the current version (Figure S1). Up to 99.54% and 99.25% of the sequences from the previous and current genomes, respectively, could be aligned using Mummer‐based alignment with a strict cut‐off (DNA identity >99%, length >10 kb). Given that the two assemblies were highly congruent and the new assembly was more complete, it was used for all subsequent analyses in this work.

### 2.2 Repetitive elements and bipartite genome architecture of *C. fructicola*

Our repeat analysis process predicted 2588 repetitive elements (REs) totalling 3.32 Mb in length from the 1104‐7 genome. Similarly, 2054 REs totalling 2.43 Mb in length were found in LJ19. At the order level (Wicker et al., 2007), total lengths of TIR DNA transposons, LINE retrotransposons and LTR retrotransposons occupied 38.17%, 32.44% and 29.39%, respectively, of the total predicted TE spaces in 1104‐7 (Figure S2a). In LJ19, total lengths of TIRs, LINEs and LTRs occupied 45.51%, 35.39% and 19.10% of the total predicted spaces, respectively (Figure S2a). Compared to LINEs and LTRs, TIRs and unknown elements were generally more divergent from the corresponding consensus sequences (Figure S2b).

Along the scaffolds, REs were dispersed unevenly. First, scaffold ends and presumptive centromere regions were abundant in REs. In 1104‐7, REs covered 60.45% of scaffold end spaces (50 kb distance range) and 82.88% of putative centromere spaces (border characterized by a rapid shift in GC content, 0.57 Mb total space). Second, short scaffolds (<1 Mb) that resembled minichromosomes contained considerably greater proportions of REs relative to long scaffolds (Figure S2c). The average RE coverage per cent values for short scaffolds (S11 and S12) in 1104‐7 and LJ19 were over five times higher than the equivalent values for long scaffolds (S1–S10). In 1104‐7, S11 and S12, which account for 2.64% of the whole genome, contained 12.95% of all annotated REs.

Repeat‐induced point mutation (RIP) is a crucial genome defence mechanism in fungi that renders repetitive DNA inactive during sexual reproduction. RIP entails C:G to T:A transition between sequence pairs that share more than 80% identity over a minimum length of 400 bp. *C. fructicola* sexual reproduction is common in nature and the 1104‐7 strain itself produces fertile perithecia on potato dextrose agar (PDA) (Liang et al., 2021). The 1104‐7 genome contains orthologues of RID (Cf1104nano2|16180) and Dim‐2 (Cf1104nano2|13031) that function in RIP in other fungi. To ascertain whether RIP contributes towards TE silencing in *C. fructicola*, we calculated the RIP index (CpA+TpG)/(ApG+GpT) values for individual REs greater or equal to 400 bp and belonging to recognized RE families with more than 10 copies. A total of 20 RE families were examined. The average RIP index value for 18 families was less than the genome‐wide average (1.22 ± 0.11, 10 kb window). In addition, the average RIP index values for nine RE families were <0.8 (Figure S2d,e), a RIP‐indicative criterion (Hane & Oliver, 2008). These features imply that RIP is active in *C. fructicola*.

### 2.3 Minichromosome‐driven two‐speed genome evolution in CGSC

**FIGURE 2:**
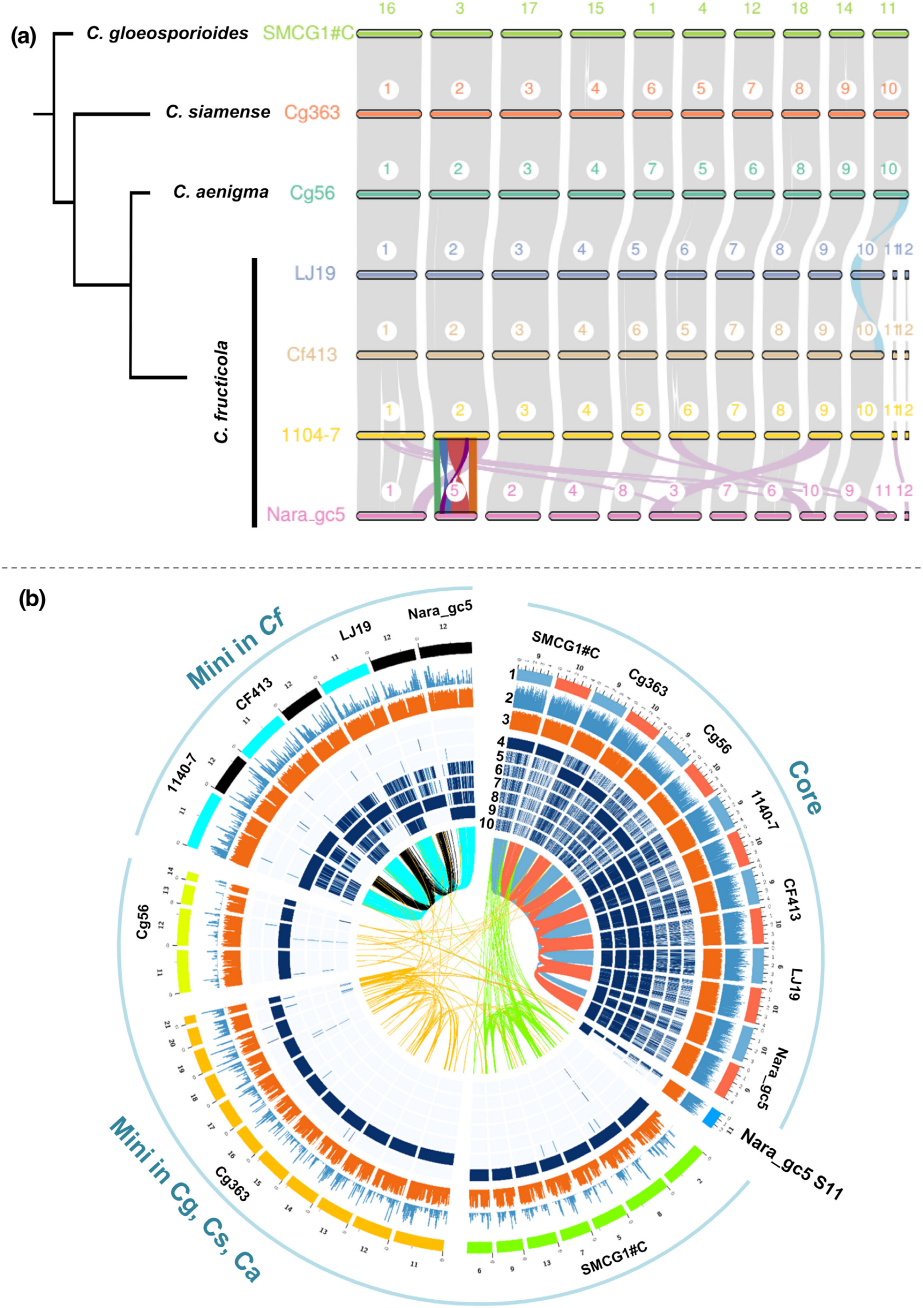
Genome architecture organization in the *Colletotrichum gloeosporioides* species complex (CGSC). (a) Macrosynteny conservation among CGSC genomes revealed by MCscan analysis. Note the conserved core chromosomes in *C. fructicola* and other CGSC species except for extensive rearrangements in Nara_gc5. Short scaffolds in SMCG1#C, Cg363 and Cg56 were omitted from display. (b) Circos plot showing different DNA sequence characteristics between core chromosomes (represented by S9 and S10 in 1104‐7) and short CGSC scaffolds (candidate minichromosomes). Tracks from outside to inside: 1, DNA track; 2, gene density; 3, GC content; 4–10, heatmaps showing the coverage rates of different query strains along 10 kb sliding windows, darker colour indicates high sequence identity. The heatmaps were based on BlastN outputs (90% identity, alignment length ≥10 kb, merged if <1 kb apart); 4, *C. gloeosporioides* SMCG1#C; 5, *C. siamense* Cg363; 6, *C. aenigma* Cg56; 7–10, *C. fructicola*; 7, 1104‐7; 8, CF413; 9, LJ19; 10, Nara_gc5. Inner lines link homologous DNA identified by Blast (90% identity, alignment length ≥10 kb).

The *C. gloeosporioides* species complex (CGSC) is made up of more than 20 closely related species. However, the complex's genomic evolution has largely gone unexplored. Prior to this study, five long‐read based chromosome‐level genome assemblies have been established within CGSC. Here, we analysed macrosynteny conservation among 1104‐7, LJ19 and the other five CGSC genomes. These seven genomes represent four CGSC species, namely *C. fructicola* (1104‐7, LJ19, CF413, Nara_gc5), *C. aenigma* (Cg56), *C. siamense* (Cg363) and *C. gloeosporioides* (SMCG1#C) (Gan et al., 2021; Huang et al., 2019). The comparison revealed two distinct groups of chromosomes (scaffolds) (Figure 2a). Group I chromosomes (corresponding to S1–S10 in 1104‐7) were long (>3 Mb) and with high levels of cross‐species collinearity, which were designated as conserved core chromosomes. Differing from group I members, chromosomes (scaffolds) in group II were shorter (0.13–0.92 Mb) and highly divergent among species (Figures 2b and S3), which were referred to as minichromosome‐like scaffolds (MLSs). Compared with core chromosomes, MLSs had much lower levels of GC content and gene density, but higher levels of RE content and higher fractions of lineage‐specific genes and unannotated genes (Figure S4). Such bipartite differentiation pattern was shared by all four CGSC species. In terms of gene functions, transporters and CAZYs were universally enriched for MLSs in all four species, whereas no uniform enrichment of small secreted proteins (SSPs), cytochrome P450s or transcription factors was observed (Figure S5).

Within *C. fructicola*, MLSs (matching to 1104‐7 scaffolds S11 and S12) displayed much lower cross‐genome alignment coverage rates and shorter alignment block lengths than core chromosomes (Figure S6). Additionally, one MLS (1104‐7 scaffold S12) exhibited intraspecific presence–absence polymorphism. A homologue scaffold was found in LJ19 and CF413 but not in Nara_gc5. Furthermore, Illumina reads mapping failed to detect this chromosome in an additional 4 out of 15 *C. fructicola* isolates (Figure 6), indicating a plastic presence–absence polymorphism of MLSs within *C. fructicola*.

### 2.4 Nascent intraspecific chromosomal rearrangements in *C. fructicola*

**FIGURE 3:**
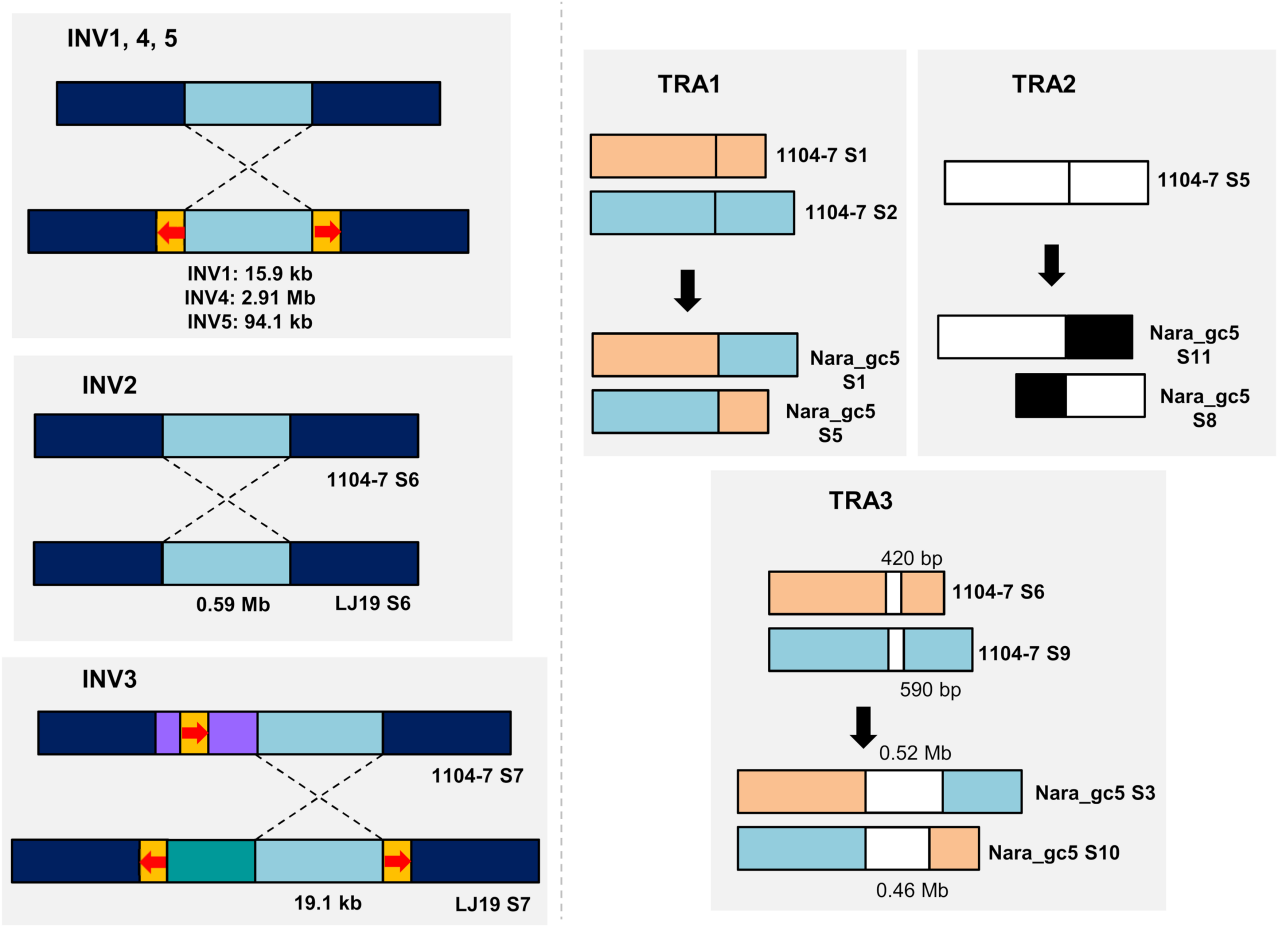
Schematic representation of identified intraspecific, isolate‐specific chromosomal rearrangement events (inversions, INV1, 4, 5; translocations, TRA1, 2, 3) on core chromosomes of *Colletotrichum fructicola* based on comparison of four high‐quality genomes (1104‐7, LJ19, Nara_gc5, CF413). For INVs (left), yellow boxes with red arrows indicate putative transposon elements. Purple and cyan boxes in INV3 indicate variable DNAs. Detailed descriptions of DNA rearrangement events for INV1–5 and TRA1–3 are listed in Figures S7 and Table S3.

Chromosomal rearrangement events can drive virulence evolution in plant‐pathogenic fungi (de Jonge et al., 2013; Faino et al., 2016), the occurrence of which, however, has not been characterized in *Colletotrichum*. Here, we annotated eight isolate‐specific rearrangement events (>10 kb, inversions and translocations) within core chromosomes of *C. fructicola* based on manual analysis of genome alignment of the four high‐quality genomes (1104‐7, LJ19, CF413, Nara_gc5). These events include five inversions and three translocations, which are summarized in Figure 3. Rearrangements of minichromosome were more complicated and were not examined in this study. Long‐read mapping validated all synteny breakpoints (BPs) (Figures [Link], [Link], [Link], [Link], [Link], [Link], [Link], [Link]). DNA sequences at each synteny BP were manually annotated to postulate the cause and functional impact of the corresponding DNA rearrangement event (Table S3, Figures [Link], [Link], [Link], [Link], [Link], [Link], [Link], [Link]). DNA inversions tend to accompany inverted repeat elements, as is the case for four out of five chromosomal inversions (1, 3, 4 and 5). Moreover, two of three translocation events (all of which took place in Nara_gc5) involved the integration of lineage‐specific DNAs at the BP locations. For translocation 2, the ancestral chromosome (represented by 1104‐7 S5) was divided into two segments in Nara_gc5 (3.88 and 0.9 Mb), which merged with two lineage‐specific DNAs (0.39 and 1.86 Mb, respectively). For translocation 3, 420 and 590 bp DNA sequences in 1104‐7 were replaced by 0.52 and 0.46 Mb lineage‐specific DNAs, respectively, in Nara_gc5. Among a total of 16 synteny BPs, four inversion BPs (inversion 2 and 4) and two translocation BPs (translocation 1) were intragenic, which may disrupt the functions of the corresponding genes (Table S3).

### 2.5 Secondary metabolite biosynthetic enzymes and candidate effectors of *C. fructicola*

**FIGURE 4:**
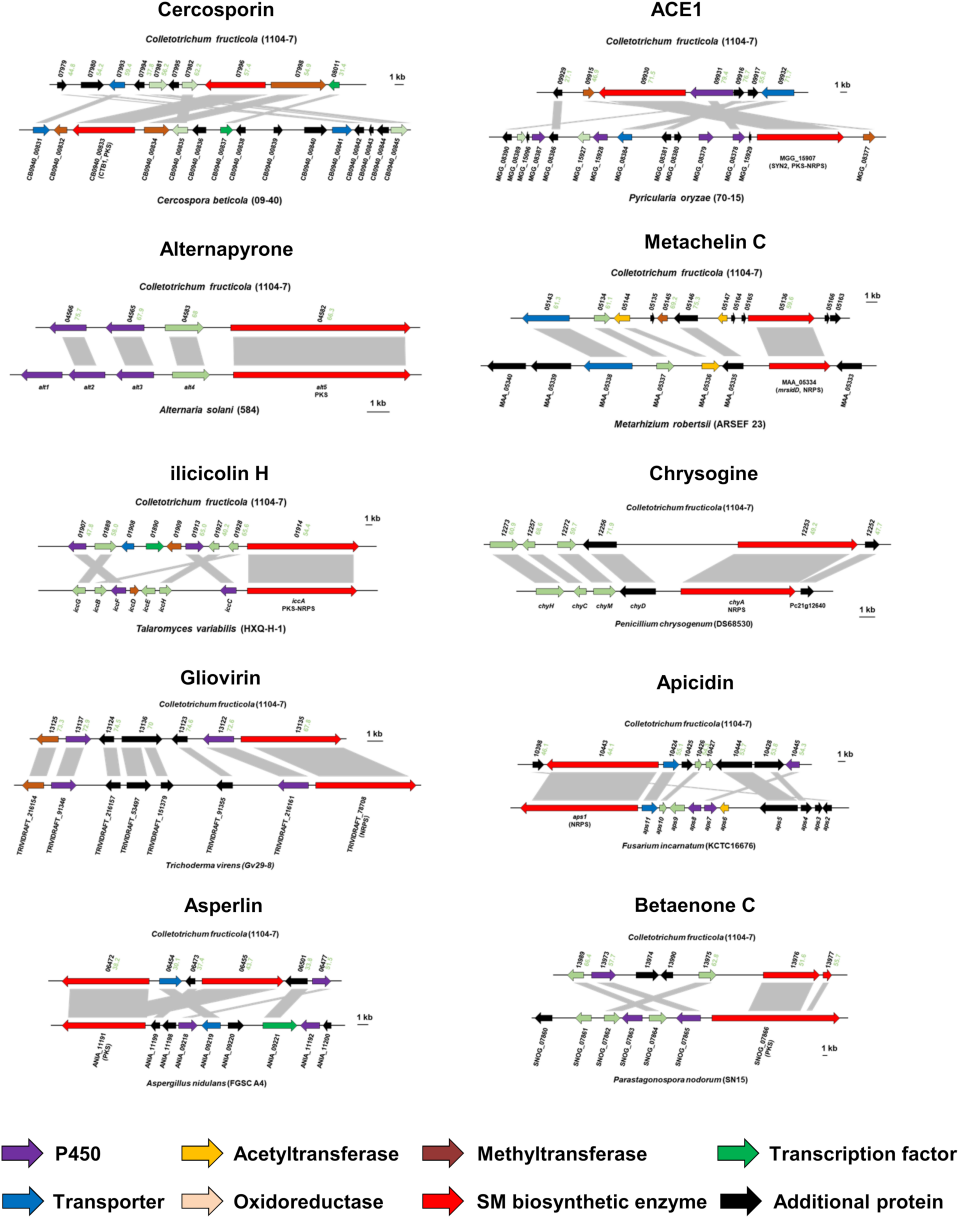
Secondary metabolite (SM) gene clusters of 1104‐7 containing shared homology and synteny with fungal SM clusters producing known metabolites. Grey boxes connect homologous genes identified by BlastP queries. Protein sequence identity information is listed in Table S4.

As hemibiotrophs, *Colletotrichum* species require the dynamic expression of a variety of virulence factors including plant cell wall‐degrading enzymes (PCWDEs), transporters, secondary metabolite (SM) synthetases and secretory effectors (Kleemann et al., 2012; O'Connell et al., 2012). Here, we annotated candidate SM synthetases and candidate effectors within the high‐quality *C. fructicola* 1104‐7 genome. Eighty putative SM biosynthesis enzymes (17 DMATs, 15 NRPSs, 5 NRPS‐PKS hybrids, 37 PKSs, 6 TSs) were predicted by SMIPS analysis and terpene synthase domain (PF03936) search. These genes were dispersed over scaffolds S1 to S10. Ten putative SM enzymes (12.3%) were found within 200 kb regions of the scaffold ends. Among the four compared CGSC species (*C. gloeosporioides*, *C. siamense*, *C. aenigma*, *C. fructicola*), 18 (22.2%) of the predicted SM enzymes displayed presence–absence polymorphism, among which one putative NRPS (Cf1104nano2|13135) was specific to *C. fructicola*. AntiSMASH prediction, homology and synteny analyses revealed 10 SM gene clusters that were strongly syntenic to fungal SM clusters producing known metabolites (Figure 4, Table S4). Among them, cercosporin and betaenone C are phytotoxic, while alternapyrone, ilicicolin H, gliovirin and asperlin have antimicrobial activities (de Jonge et al., 2018; Fujii et al., 2005; Grau et al., 2018; Li et al., 2019; Sherkhane et al., 2017; Singh et al., 2012), metachelin C is a coprogen‐type siderophore functioning in iron assimilation (Krasnoff et al., 2014), and apicidin is a conserved histone deacetylase inhibitor (Jin et al., 2010), ACE1 (a cytochalasan‐related molecule) is presumed to promote appressorium‐mediated penetration in *Magnaporthe grisea* (Collemare et al., 2008). Therefore, SMs produced by *C. fructicola* may have diverse roles in microbial pathogenicity, competition and other processes. It is worth noting that SM gene clusters related to ACE1, alternapyrone and cercosporin have also been found in *C. higginsianum* (Dallery et al., 2017).

**FIGURE 5:**
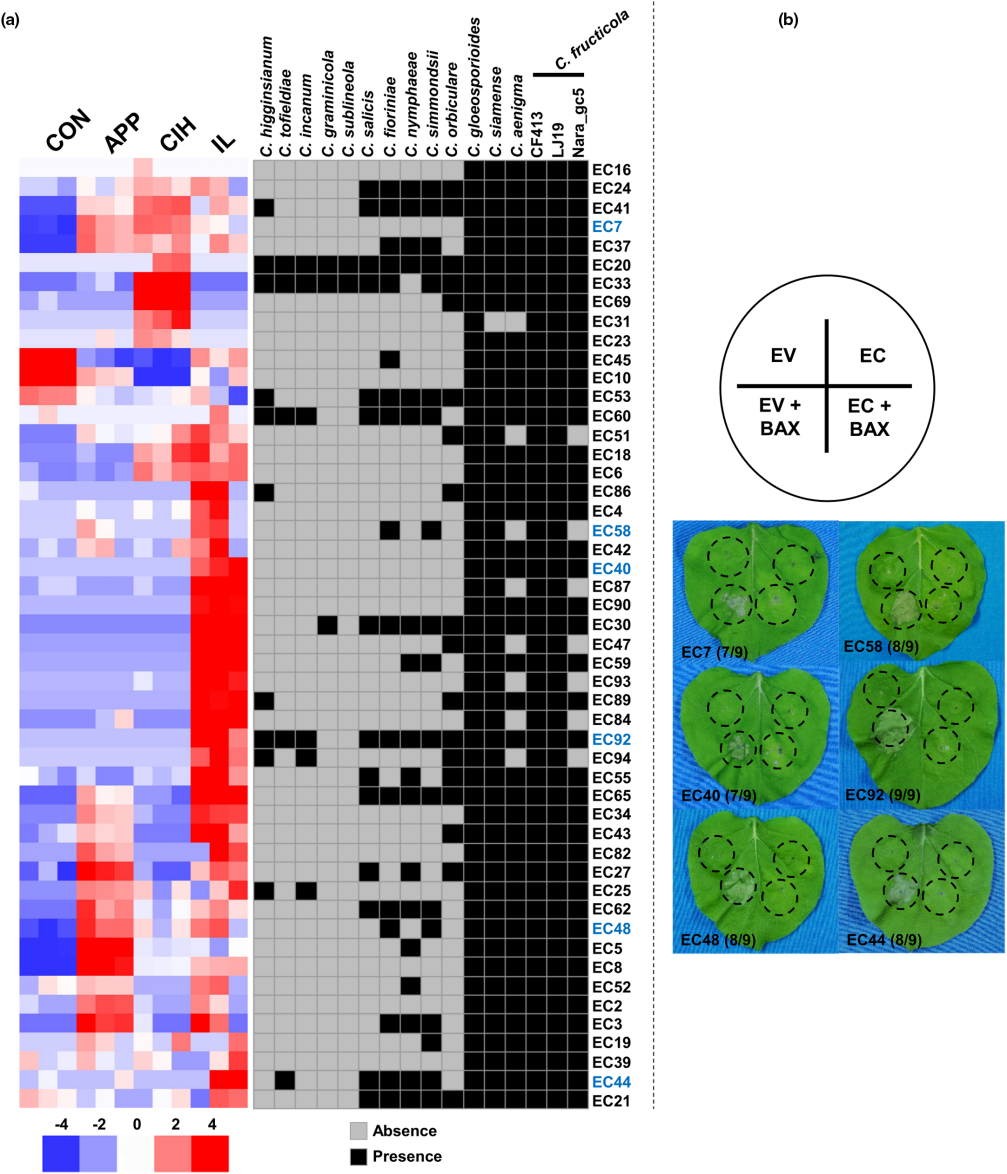
Expression, presence–absence polymorphism and functional characterization of a set of genus‐specific effector candidate (EC) genes within *C. fructicola*. (a) Heatmap showing the expression profiles of ECs (left) and their presence–absence polymorphism patterns among *Colletotrichum* genomes (right). The transcriptomic heatmap is based on an RNA‐seq dataset previously published (Liang, Shang, et al., 2018) and the presence–absence heatmap is based on BlastP queries of *C. fructicola* ECs against other *Colletotrichum* genomes. ECs with cell death‐suppressive activities (shown in panel b) are coloured in blue. (b) ECs with cell death‐suppressive activity based on a *Nicotiana benthamiana* transient co‐expression assay system. Top, the infiltration scheme, leaves were infiltrated with *Agrobacterium tumefaciens* carrying pGR107 empty vector (EV) or EV inserted with the EC gene (EC) alone or in a mixture with *A. tumefaciens* expressing the cell death‐promoting protein BAX (EV + BAX or EC + BAX). Bottom, identified ECs with death‐suppressive activity. For each EC, the number of leaves showing death‐suppressive activity and the total infiltrated leaves are indicated in parentheses.

Our bioinformatics pipeline (see Experimental Procedures) predicted 1490 putative secretory proteins in 1104‐7, 631 of which were classified as small secretory proteins (SSPs) because they contained fewer than 300 amino acids. Based on OrthoFinder clustering and a local BlastP search against 29 ascomycete genomes (11 non‐*Colletotrichum* and 19 *Colletotrichum* ones), 331 SSPs were classified into a *Colletotrichum* genus‐specific group (Figure 5a). Based on a *Nicotiana benthamiana* transient expression system, genus‐specific SSPs were further characterized for their cell death‐suppressive or ‐promoting activities. Of the 50 screened members, six could suppress BAX‐induced cell death (Figure 5b), among which two have been demonstrated to be required for full virulence of *C. fructicola* (Shang et al., 2020, 2024).

### 2.6 Identification of lineage‐specific genes critical for apple GLS pathogenesis

**FIGURE 6:**
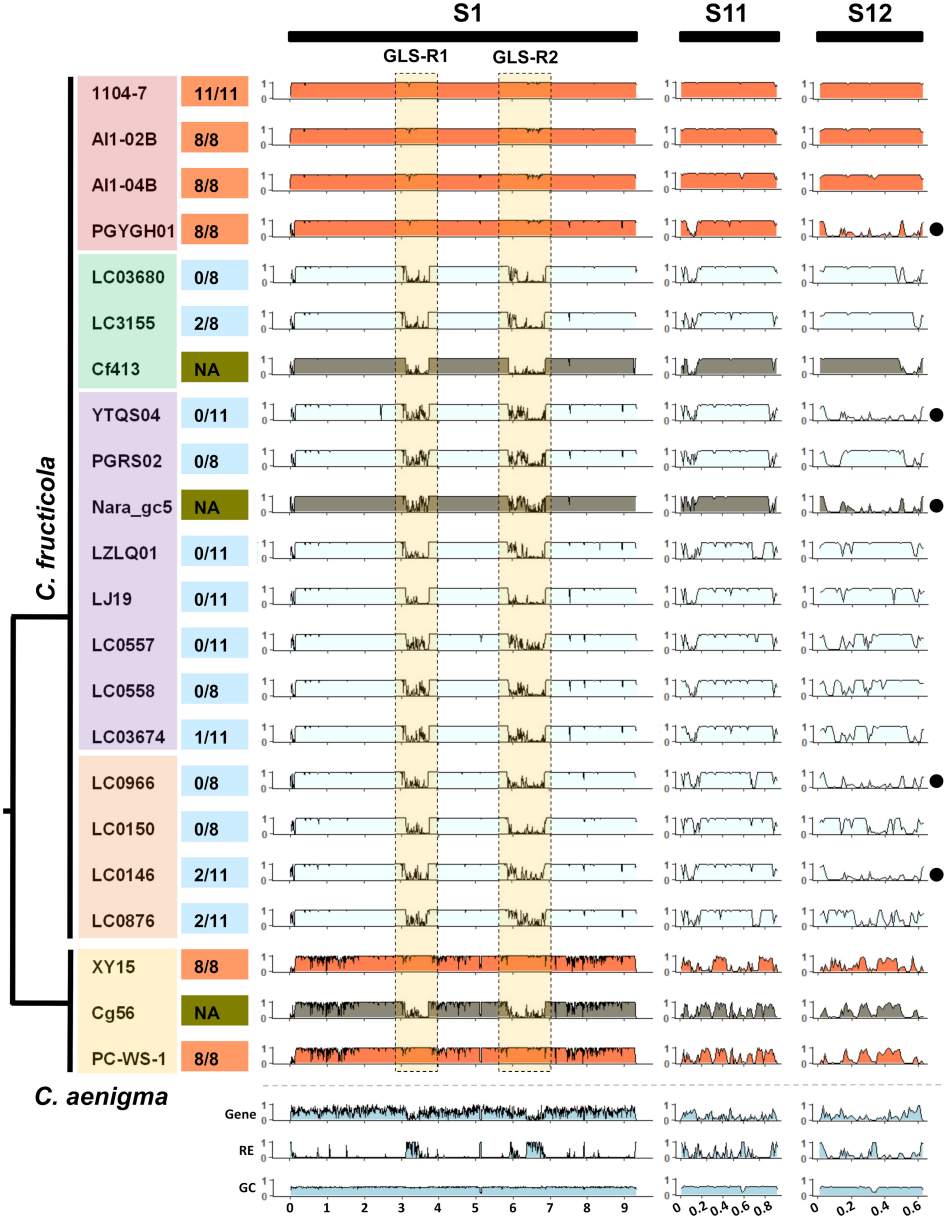
DNA presence–absence polymorphism along scaffold S1, S11 and S12 in 1104‐7 among *Colletotrichum fructicola* and *C. aenigma* isolates. Two Glomerella leaf spot (GLS)‐specific regions (GLS‐R1 and GLS‐R2) on scaffold S1 are indicated by dash boxes and scaffold S11 and S12 are two putative minichromosomes. Relationship of the isolates is based on genome‐wide single‐nucleotide polymorphism (SNP) phylogram in Figure S15, isolates shaded in the same colour belong to the same clade. It is worth noting that such a phylogram‐based approach only provides a rough estimation of intraspecific relationships due to potential assumption violation. Numbers separated by slash on the right of isolates indicate the number of leaves showing GLS symptoms and the total inoculated leaves, NA indicates not assessed. For each isolate, DNA sequence read coverage against 1104‐7 (10 kb sliding window) is presented as histograms. Histograms of gene density (Gene), repeat element density (RE) and GC content (GC) for the 1104‐7 reference scaffolds are presented at the bottom. Note that high reads coverage for GLS‐R1 and GLS‐R2 are specifically observed among GLS‐pathogenic isolates (orange), and low reads coverage along S12 was observed in five *C. fructicola* isolates (filled black circle), indicating intraspecific dispensability of this minichromosome.

*C. fructicola* and *C. aenigma* are known to cause apple GLS disease in China (Chen et al., 2022). *C. fructicola* has a broad host range, causing damage to over 50 plant species. However, reported intraspecific pathogenic differentiation indicates that this fungus may comprise forms with distinct host preferences (Rockenbach et al., 2016). We resequenced 15 *C. fructicola* isolates and two *C. aenigma* isolates from different hosts and geographic origins using Illumina technology (Dataset S1) to analyse possible genomic variation associated with GLS pathogenicity evolution. These 17 isolates, as well as 1104‐7 and LJ19, were subjected to an artificial inoculation assay (Figures 6 and S15), which demonstrated that all four *C. fructicola* isolates obtained from apple GLS lesions consistently exhibited GLS symptoms on Gala apple leaves, whereas the remaining 13 *C. fructicola* isolates from apple fruit or non‐apple hosts did not. The two *C. aenigma* isolates derived from GLS lesions (XY15, PC‐WS‐1) were also pathogenic on Gala apple leaves. A phylogenetic tree built with 21,741 parsimony‐informative single‐nucleotide polymorphism (SNP) sites located within 1326 core fungal genes (single‐copy core genes derived from BUSCO analysis) separated *C. fructicola* isolates into four groups, with all four GLS‐pathogenic isolates belonging to the same group (Figures 6 and S15). *C. aenigma* isolates were well separated from *C. fructicola* isolates (Figures 6 and S15), confirming their species separation.

Genomic regions and genes showing variations among *C. fructicola* and *C. aenigma* isolates were identified by mapping Illumina reads or PacBio reads from different isolates against the 1104‐7 reference genome. Table S5 summarizes the genome resequencing and read mapping statistics for individual isolates. The average reads depths ranged between 18.5 and 205.4. As already mentioned, one minichromosome (scaffold S12) exhibits presence–absence polymorphism among the compared *C. fructicola* isolates (Figures 6 and S16). In addition, subtelomeric regions were highly variable (Figures 6 and S16). At the gene level, 16,689 (88.28%) genes were designated as *C. fructicola* core genes (bases with non‐zero coverage >95% in all compared *C. fructicola* isolates), whereas 896 (4.72%) genes were designated as variable (bases with non‐zero coverage <80% in at least three isolates). Interestingly, two subregions of 1104‐7 genome (scaffold S1, 3.12–3.74 and 5.88–6.87 Mb) were GLS specific, with a considerably greater coverage fractions for reads derived from GLS isolates than those from non‐GLS isolates (Figures 6 and S16). These two regions, totalling 1.61 Mb in length, are referred to as GLS‐R1 (GLS‐Region 1) and GLS‐R2 (GLS‐Region 2). Association of GLS‐R1 and GLS‐R2 with GLS pathogenicity was also observed in *C. aenigma*. The two regions were specifically present in the two apple‐derived GLS isolates (XY15, PC‐WS‐1) but not in the strawberry‐derived isolate Cg56. GLS‐R1 and GLS‐R2's trans‐species association with GLS pathogenicity is consistent with a function in GLS pathogenesis.

**FIGURE 7:**
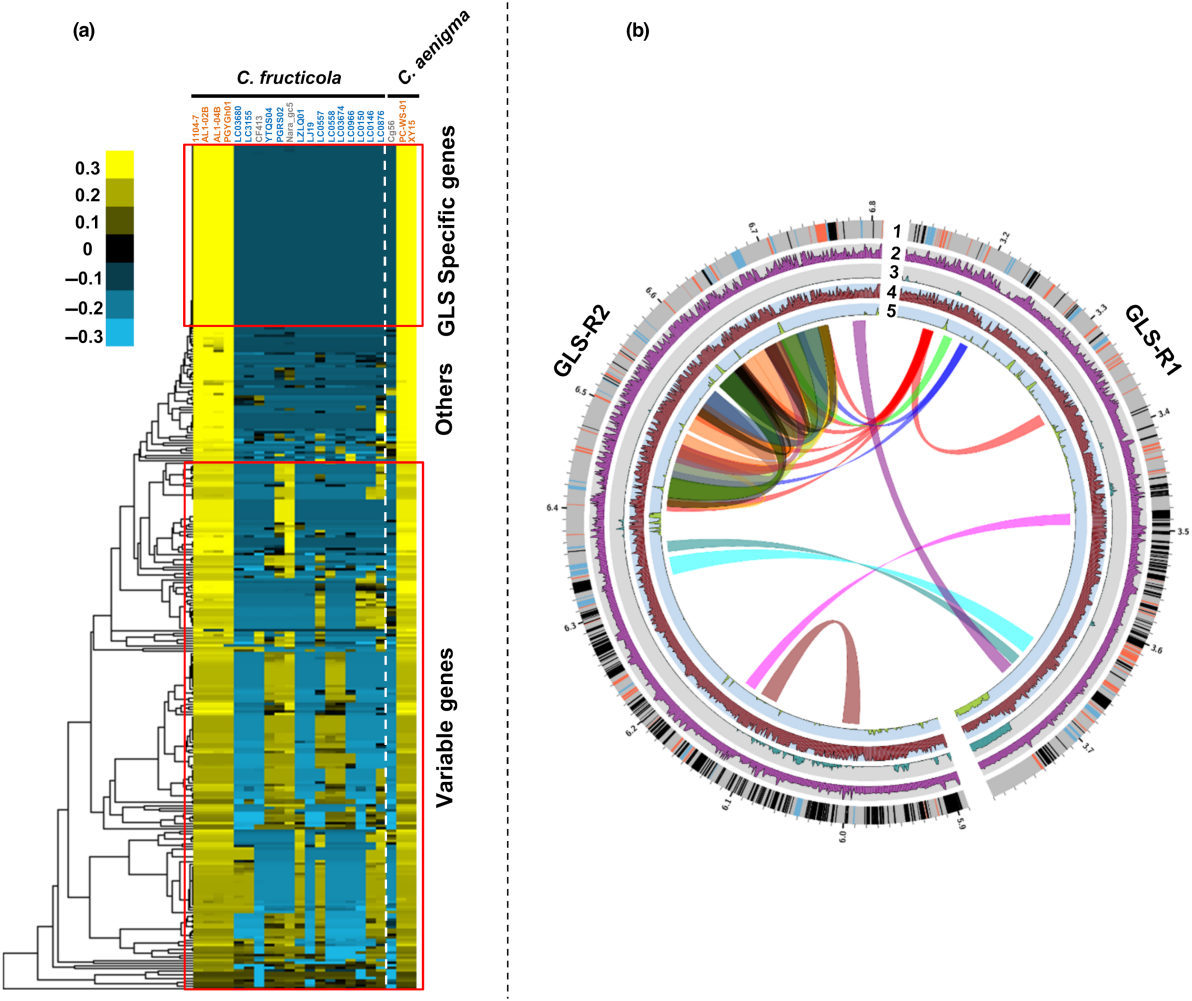
Identification of Glomerella leaf spot (GLS)‐specific genes within the two GLS‐specific subgenomic regions (GLS‐R1 and GLS‐R2). (a) Clustering of 333 predicted protein‐coding genes into GLS‐specific genes, variable genes and others based on variation in the breadth of read coverage (fraction of bases with non‐zero coverage) among 22 *Colletotrichum fructicola* and *C. aenigma* isolates. Higher heatmap scales indicate higher read coverage breadth. Isolate names are coloured based on GLS pathogenicity assay result. Orange: GLS‐pathogenic, light blue: GLS‐nonpathogenic, grey: not assessed but putatively GLS‐nonpathogenic. (b) Circos plot showing the distribution of the three gene types along chromosome. Track 1, gene category information. Orange: GLS‐specific genes, black: variable genes, blue: others. Tracks 2–5, breadth of read coverage for PGYGH01 (*C. fructicola*, GLS‐pathogenic), LC03680 (*C. fructicola*, GLS‐nonpathogenic), XY15 (*C. aenigma*, GLS‐nonpathogenic) and Cg56 (*C. aenigma*, putatively GLS‐nonpathogenic), respectively. Inner links represent major repeat elements considering both unit length and copy number.

The GLS‐R1 and GLS‐R2 regions were highly repetitive (Figure S16). Four hundred and twenty‐four repeat elements were identified within these two regions, covering 0.67 Mb total DNA (41.6%), a ratio that is significantly higher than the genome background (5.67%). A total of 331 protein‐coding genes were predicted within GLS‐R1 and GLS‐R2 (Dataset S2). By performing hierarchical clustering, these genes were classified into GLS‐specific genes (76 in total), variable genes (208 in total) and GLS‐associated genes (49 in total) based on variation in the breadth of coverage among reads derived from 22 *C. fructicola* and *C. aenigma* isolates (Figure 7a). GLS‐specific genes strongly correlated with GLS pathogenicity in reads presence–absence pattern, with bases with non‐zero coverage >95% in all six GLS isolates, but <10% in all other isolates. GLS‐associated genes were generally specific to the six GLS isolates but may be present in an additional one or two non‐GLS isolates, or detected in non‐GLS isolates with low breadth of coverage. The distribution of different gene groups within GLS‐R1 and GLS‐R2 was somewhat compartmentalized. For instance, two regions (3.14–3.45 and 6.31–6.79 Mb) mainly contained GLS‐specific genes (44.7%) and GLS‐associated genes (31.1%) (Figure 7b). Functional enrichment analysis suggested that GLS‐specific genes were enriched with functions related to secondary metabolism (e.g., AMP‐binding enzyme, UDP‐glucoronosyl and UDP‐glucosyl transferase, chorismate binding enzyme) (Tables [Link], [Link], [Link]).

**FIGURE 8:**
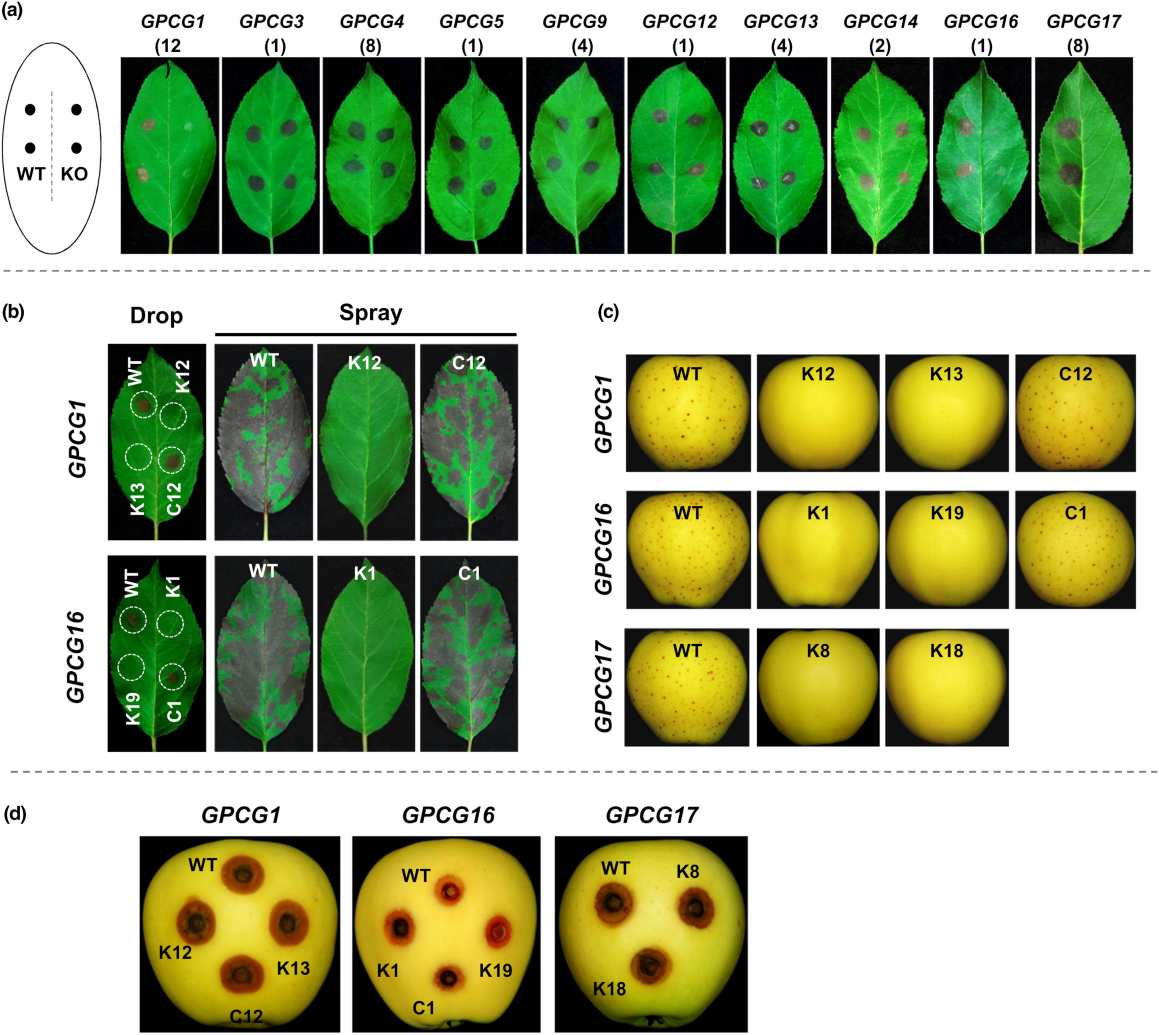
Virulence phenotypes of gene deletion mutants of the 10 Glomerella leaf spot (GLS) pathogenicity candidate genes (*GPCG*s). (a) Lesion symptom appearance on detached Gala apple leaves at 5 days after conidial drop inoculation (10^7^ spores/mL). Wild‐type (WT) and individual gene deletion mutants (KO) were pair inoculated on individual leaflets, each with two replicates. The number within parentheses represents the KO strain designation. (b) Genetic complementation of the *GPCG1* and *GPCG16* KO mutants. Conidial suspensions (10^7^/mL) were either drop inoculated (left) or spray inoculated (right). K, KO strain. C, complementation strain. (c) Lesion appearance on Gala apple fruit at 15 days after conidial spray inoculation (10^7^/mL). (d) Lesion appearance on Gala apple fruit at 5 days after conidial drop inoculation (10^7^/mL) at prewounded sites.

To determine whether the identified GLS‐R1 and GLS‐R2 regions indeed contribute towards GLS pathogenicity, we chose 10 genes within the two regions (GLS pathogenicity candidate genes, *GPCG*s) for gene deletion analysis (Table 1). These genes were selected with a combined consideration of virulence‐related gene function, infection‐specific expressional up‐regulation (based on previous RNA‐seq data) and presence specificity among *C. fructicola* and *C. aenigma* GLS isolates. These 10 GPCGs included eight GLS‐specific genes (*GPCG 1, 4, 5, 12, 13, 14, 16, 17*), one GLS‐associated gene (*GPCG3*, a putative β‐ketoacyl synthase) and one variable gene (*GPCG9*, a putative fungal specific transcription factor). For each gene, two independent deletion mutants were created and used for phenotypic characterization. Figure S17 shows the PCR‐based validation of gene deletion events for each gene. In virulence assay with detached apple leaves, deletion of any of the three *GPCG*s (*1*, *16*, *17*) completely abolished GLS lesion formation, whereas deletion of any of the other seven *GPCG*s (*3, 4, 5, 9, 12, 13, 14*) had no obvious effect (Figure 8a). The GLS virulence defects of *GPCG1*, *GPCG16* and *GPCG17* mutants were consistently observed in both leaf and fruit inoculation assays (Figure 8b,c). The mutant virulence defects for *GPCG1* and *GPCG16* were fully restored by genetic complementation. Genetic complementation was not attempted for *GPCG17* as it encodes a very large protein (NRPS). To determine whether *GPCG1*, *GPCG16* and *GPCG17* contribute towards ABR pathogenicity, inoculation assays were performed with prewounded apple fruits, in which case ABR‐mimicking rot lesion symptoms would be induced. The results demonstrated that deletion of any of the three genes did not affect rot lesion formation (Figure 8d). We concluded that *GPCG1*, *GPCG16* and *GPCG17* are key genes regulating GLS pathogenesis, but dispensable for ABR lesion induction on wounded fruit.

**TABLE 1:** Predicted functions of Glomerella leaf spot (GLS) pathogenicity candidate genes (*GPCGs*) chosen for gene deletion analysis.

GPCG	Gene ID	Position (start)	Position (end)	Protein length (amino acids)	Functional annotation	Note
*GPCG1*	Cf1104nano2|08589	3,255,843	3,257,854	512	Flavin‐binding monooxygenase‐like, PF00743	GLS‐specific gene
*GPCG3*	Cf1104nano2|08602	3,319,943	3,323,359	774	Beta‐ketoacyl synthase, PF00698, PF00109	GLS‐associated gene, detected not only in all 6 GLS isolates, but also in 11 additional isolates with low breadth of coverage (10%–30%)
*GPCG4*	Cf1104nano2|08624	3,429,383	3,429,676	97	Small protein with unknown function, no PFAM domain, no predicted signal peptide	GLS‐specific gene
*GPCG5*	Cf1104nano2|08618	3,434,586	3,435,755	389	NAD(P)‐binding Rossmann‐like, PF13450	GLS‐specific gene
*GPCG9*	Cf1104nano2|09434	6,171,970	6,173,958	635	Fungal‐specific transcription factor domain‐containing protein, PF04082	Variable gene, detected in all 6 GLS isolates and 2 non‐GLS isolates
*GPCG12*	Cf1104nano2|09458	6,223,384	6,224,681	360	Protein with unknown function, no PFAM domain	GLS‐specific gene
*GPCG13*	Cf1104nano2|09446	6,225,534	6,227,370	531	Cytochrome P450, PF00067	GLS‐specific gene
*GPCG14*	Cf1104nano2|09459	6,240,422	6,241,391	229	Histidine phosphatase superfamily, PF00300	GLS‐specific gene
*GPCG16*	Cf1104nano2|09535	6,744,619	6,744,930	103	Small protein with unknown function, no PFAM domain, no predicted signal peptide	GLS‐specific gene
*GPCG17* ^*a*^	Cf1104nano2|09529	6,750,297	6,768,345	4746	Non‐ribosomal peptide synthetase (NRPS), PF00501, PF00668, PF00550	GLS‐specific gene

*Note*: *GPCG17* ^*a*^ represents a manual combination of three neighbouring genes (Cf1104nano2|09529, Cf1104nano2|09530 and Cf1104nano2|09531) that harbour protein domains related to a non‐ribosomal peptide synthetase (NRPS). Manual prediction of the putative coding regions and protein‐encoding sequence is shown in Dataset S3.

We characterized the functions of *GPCG1*, *GPCG16* and *GPCG17* in more detail. Reverse transcription–quantitative PCR (RT‐qPCR) quantification of gene expression showed that the three genes had similar expression patterns, with expression levels being low in conidia and in vitro‐grown vegetative hyphae, mildly induced in in vitro appressoria and during wounded fruit infection, but considerably higher during apple leaf infection (Figure S18). Gene expression of the three genes peaked at 60 h post‐inoculation (hpi) during apple leaf infection, increased by roughly 4000‐, 130‐ and 450‐fold, respectively, compared to conidia. Deletion of *GPCG1*, *GPCG16* or *GPCG17* did not affect fungal colony growth on PDA, perithecial development or conidial germination (Figure S19a,b); more importantly, deletion of these genes did not affect appressorium differentiation or appressorium‐mediated penetration on artificial cellophane membrane (Figure S19c). These results suggest that the three genes play post‐penetration virulence‐specific functions. To better understand at which infection stage the *GPCG1*, *GPCG16* and *GPCG17* genes are functional, we performed histological observations with apple leaf samples inoculated with different isolates (Figure S20). Deletion mutants of the three genes showed normal conidial germination and appressorium differentiation on apple leaf surface but were defective in the development of the primary infectious vesicle and infectious hyphae, suggesting that the mutants are blocked at the early post‐penetration infection phase.

We conducted bioinformatics analysis with *GPCG1*, *GPCG16* and *GPCG17* to infer their potential biochemical functions and evolution (Figure S21a). The *GPCG1* gene is within GLS‐R1 and encodes a putative flavin‐binding monooxygenase. Within GLS‐R2, *GPCG16* encodes a small protein (150 amino acids) with no predicted signal peptide or protein domains and *GPCG17* encodes a putative non‐ribosomal peptide synthetase (NRPS). Interestingly, *GPCG16* and *GPCG17* are adjacent genes separated by 4048 bp. Possibly, *GPCG1*, *GPCG16* and *GPCG17*, together with additional unidentified genes within GLS‐R1 and GLS‐R2, cooperatively control the biosynthesis of a secondary metabolite critical for early GLS pathogenesis. To learn about the evolution of *GPCG1*, *GPCG16* and *GPCG17* genes, we examined the distribution patterns of their homologues in NCBI nr database by performing a BlastP search. In line with the fact that the three genes are GLS‐lineage specific, BlastP queries only identified distant homologues (30%–40% amino acid identities), further phylogenetic analysis confirmed the deep separation of GPCG1, GPCG16 and GPCG17 from their *Colletotrichum* and non‐*Colletotrichum* homologues (Figure S21b).

## 3 DISCUSSION

Since its discovery in the United States in the 1970s, GLS disease has been reported primarily in North and South America, as well as East Asia (Liang et al., 2022; Velho et al., 2015). Despite the disease's recent origins and narrow geographic reach, pathogen species diversity is very high. To date, nine GLS species have been reported globally, and they are members of three phylogenetically distinct species complexes (CGSC, CASC and CBSC) (Liang et al., 2022). The short disease history, intraspecific pathogenic differentiation and polyphyletic distribution of GLS pathogen species indicate a multiple origin scenario of GLS pathogenicity that involves the horizontal transfer of a pathogenicity determinant(s). However, such an evolutionary possibility has not been investigated. In this study, we performed genome comparisons with *C. fructicola* and *C. aenigma*, and identified two accessory genomic regions (GLS‐R1, GLS‐R2) that were specifically conserved among GLS‐pathogenic isolates in both species. Moreover, three genes (*PCG1*, *PCG16*, *PCG17*) within these two regions were found by gene deletion studies to be crucial for GLS pathogenicity. These results highlight the critical involvement of lineage‐specific DNA in GLS pathogenicity evolution.

For filamentous fungal pathogens, various processes may drive the evolution of host adaptation, such as chromosomal rearrangement (de Jonge et al., 2013), presence or absence of dispensable minichromosome (Ma et al., 2010), horizontal gene transfer (McDonald et al., 2019; Zhao et al., 2014), gene gain or loss (Dhillon et al., 2015; Sharma et al., 2014; Zajac et al., 2021) and positive selection of genes (Kobmoo et al., 2018; Sperschneider et al., 2014). At the genome level, the need to strike a balance between the rapid evolution of virulence genes and the maintenance of housekeeping genes has driven the compartmentalization of pathogen genomes, with repeat‐rich, fast‐evolving, accessory genomic regions acting as a cradle for host‐adaptive virulence gene evolution (Croll & McDonald, 2012; Dong et al., 2015; Frantzeskakis et al., 2019). GLS‐R1 and GLS‐R2 are repeat‐rich and gene sparse, phylogenetic analysis of *PCG1*, *PCG16* and *PCG17* genes showed that they are deeply separated from their *Colletotrichum* and non‐*Colletotrichum* homologues. We postulate that GLS‐R1 and GLS‐R2 represent horizontally transferred DNAs from an unidentified source. Both GLS‐R1 and GLS‐R2 localize to scaffold S1, the longest chromosome in 1104‐7. In comparison with other *C. fructicola* genomes (LJ19, CF413, Nara_gc5), the GLS‐R1 and GLS‐R2 insertions are flanked by 6‐bp long direct repeats (CCCTCA and TTTACT, respectively; Figure S22) and no transposon elements were found close to the synteny BPs. Interestingly, two DUF3435 integrase‐like genes within GLS‐specific regions (Cf1104nano2|08705 and Cf1104nano2|09336) located near (576 bp) the right synteny BP of GLS‐R1 and near (450 bp) the left synteny BP of GLS‐R2, respectively (data not shown). These characteristics suggest that there might be active mechanisms facilitating the interspecific transfer of GLS‐R1 and GLS‐R2.

Minichromosome‐mediated genome compartmentalization has been demonstrated to be important for virulence evolution in *Colletotrichum* fungi (Plaumann & Koch, 2020). For instance, intraspecific variation in the number and size of minichromosomes is linked to virulence variation in *C. gloeosporioides* (He et al., 1998; Masel et al., 1990), one of the two minichromosomes in *C. higginsianum* is required for full virulence against *Arabidopsis thaliana* (Plaumann et al., 2018), and in a cross between two strains of *C. lentis*, a potent minichromosome‐associated quantitative trait locus (QTL) accounts for 85% of the virulence variability (Bhadauria et al., 2019). Moreover, a recent comparative genomic study with CGSC species highlights that telomeres and repeat‐rich minichromosomes are enriched with virulence‐related accessory genomic regions (Gan et al., 2021). In this study, we compared chromosome‐level genome assemblies from four CGSC species, *C. gloeosporioides*, *C. siamense*, *C. aenigma* and *C. fructicola*. While our comparisons highlight clear distinctions between core and minichromosomes in evolutionary speed, we did not find direct correlation between GLS pathogenicity differentiation and the presence–absence pattern of a specific minichromosome or minichromosome region. Yet, it is worth noting that core chromosome‐located GLS‐R1 and GLS‐R2 strongly resembles CGSC minichromosomes for being repeat rich, gene sparse and enriched with lineage‐specific genes. In the blast fungus *Magnaporthe oryzae* (syn. *Pyricularia oryzae*), structure rearrangements and segmental duplication of core chromosomes have been indicated to contribute to the emergence of minichromosomes and the reshuffling of virulence‐related genes (e.g., effectors) (Langner et al., 2021; Peng et al., 2019). Further genomic comparison studies with additional chromosome‐scale assemblies of GLS isolates would be helpful for elucidating the evolutionary history of GLS‐R1 and GLS‐R2.

Our genome comparisons also demonstrated the intraspecific presence–absence polymorphism of a minichromosome (1104‐7 scaffold S12) within *C. fructicola*. This chromosome was present in 14 out of 19 *C. fructicola* isolates and a whole genome phylogram (Figure 6) revealed that the five S12‐lacking isolates belonged to three polyphyletic clades. S12 appears to be genetically unstable and experiences frequent losses in nature. In *Verticillium longisporum*, partial or complete deletion of a 20‐kb lineage‐specific DNA region increases virulence, suggesting that lineage‐specific DNA can confer a virulence‐attenuating function (Harting et al., 2021). The functional implications of the observed minichromosome loss event in *C. fructicola* remain to be investigated in the future.

Conidia of gene deletion mutants of *PCG1*, *PCG16* and *PCG17* germinated and differentiated appressoria normally on inoculated apple leaf surface. However, post‐invasive infectious development of these mutants was very constrained. In a separate assay, these mutants' in vitro appressoria were able to penetrate a cellophane artificial membrane with an efficiency comparable to the wild type. We concluded that *PCG1*, *PCG16* and *PCG17* play virulence functions by promoting early infection, perhaps by inhibiting plant defence responses. In line with this hypothesis, *PCG1*, *PCG16* and *PCG17* all had in planta‐specific gene expression patterns. Functionally, *PCG1* encodes a putative flavin‐binding monooxygenase, *PCG17* encodes a putative NRPS and *PCG16* encodes an unknown protein but neighbours *PCG17*. These findings suggest that these three genes belong to a gene group that cooperates to catalyse the biosynthesis of a secondary metabolite important for GLS pathogenicity. *PCG1*, *PCG16* and *PCG17* lacked closely related homologues both inside and outside of the *Colletotrichum* genus, complicating the analysis of their evolutionary origin(s). A genetic mapping study has demonstrated a single recessive locus controlling apple GLS resistance (Liu et al., 2017). The presence of a pathogenicity‐determining secondary metabolite would therefore be in line with an inverse gene‐for‐gene interaction model where host–pathogen recognition mediates virulence and disease compatibility. Such a genetic model is similar to the role of host‐selective toxins (HSTs) reported for necrotrophic fungi (Wolpert et al., 2002), except that the GLS‐associated metabolite functions as a plant defence suppressor. The in planta‐specific expression characteristics of *PCG1*, *PCG16* and *PCG17* has added difficulty to metabolite isolation and characterization. In the future, screening for genetic regulators or environmental conditions that permit derepressed expression of PCG genes under in vitro conditions would be important for dissecting the function of the GLS‐determining metabolite.

In summary, we have generated chromosome‐level genome assemblies for two *C. fructicola* isolates and identified a conserved bipartite genome architecture involving minichromosomes among several CGSC species. By performing genome resequencing and comparison, we identified two GLS‐specific genomic regions and identified three genes within the regions to be critical for GLS pathogenesis. These results shed important insights into adaptative evolution in *Colletotrichum* fungi and lay a foundation for further dissection of the pathogenicity mechanisms of GLS pathogens.

## 4 EXPERIMENTAL PROCEDURES

### 4.1 Fungal isolates

Detailed information of all *C. fructicola* and *C. aenigma* isolates used in this study can be found in Dataset S1. Isolates were cultured on PDA and preserved as 15% glycerol conidial stocks at −80°C in the Fungal Laboratory, College of Plant Protection, Northwest A&F University.

### 4.2 Sequencing, assembly and functional annotation of 1104‐7 and LJ19 genomes

Procedure for genomic DNA extraction and nanopore sequencing of 1104‐7 has been described previously (Liang et al., 2020), the same pipeline has been applied for LJ19 to obtain nanopore reads. The obtained nanopore reads for 1104‐7 and LJ19 were assembled with the software NextDenovo (https://github.com/Nextomics/NextDenovo). In both assembly procedures, the read_cutoff value was set to 1 kb and the minimum seed length (seed_cutoff) values were set based on seq_stat recommendations. The obtained long‐read assemblies were further polished with NextPolish (Hu et al., 2019) using NGS reads of the corresponding isolates as input. The whole genome assemblies have been deposited in GenBank under the accession numbers MVNS00000000 and CP132168–CP132179. The assembly completeness was evaluated by searching for conserved genes in the fungi_odb9 library (1438 total genes), using BUSCO v. 1.2 (Simão et al., 2015).

RECON v. 1.08 (Bao & Eddy, 2002) and RepeatScout v. 1.0.5 (Price et al., 2005) integrated into Repeatmodeler v. 1.0.11 were used for de novo identification of repetitive sequences in the assembled 1104‐7 and LJ19 genomes. LTRharvest (Ellinghaus et al., 2008) from GenomeTools v. 1.5.9 (Gremme et al., 2013) was used to improve the detection of LTR‐retrotransposons. The program RepeatMasker v. 4.0.6 was also employed for repetitive sequence identification with a custom library made up of 5623 fungal transposable elements (TEs) from RepetDB (Ameslem et al., 2019) and 41 manually annotated *C. higginsianum* TEs (Dallery et al., 2017). The outputs from Repeatmodeler, LTR and RepeatMasker were merged and clustered at 80% similarity using the cluster_fast command in USEARCH v. 11 (Edgar, 2010). RepeatMasker was run again using a custom library composed of the obtained non‐redundant consensus sequences generated from USEARCH. Finally, the RepeatMasker output was parsed using the ‘One_code_to_find_them_all’ perl script (Bailly‐Bechet et al., 2014) to join neighbouring TE fragments. The length cut‐off for repeat elements was set to 80 bp. For repeat family annotation, each consensus sequence was classified based on its best BlastX hit against the Repbase peptide database (downloaded on 7 August 2015) and sequences lacking significant hit (*E*‐value cut‐off = 1e−05) were labelled as ‘unknown’. GC content, dinucleotide frequencies, and RIP indices were calculated based on the R package Biostrings v. 2.52.0.

To predict gene structures in the 1104‐7 and LJ19 genomes, a pipeline combining Augustus v. 3.1 (Stanke & Waack, 2003), GeneMark‐ES v. 2.3c (Ter‐Hovhannisyan et al., 2008) and MAKER2 v. 2.31.8 (Holt & Yandell, 2011) predictions was applied as previously described (Liang et al., 2020). Predicted genes were functionally annotated by BlastP search against a local NCBI nr database and by querying against a local Interproscan database. Identification of putative secondary metabolite genes, transcription factors, cytochrome P450s and SSPs was performed as previously described (Liang, Shang, et al., 2018; Liang, Wang, et al., 2018).

### 4.3 Genome comparison and resequencing analyses

Whole‐genome alignments among putative chromosomes of CGSC genome assemblies were performed with MUMer (Kurtz et al., 2004). The alignments were mined for intraspecific isolate‐specific rearrangement events (>10 kb) among the four high‐quality genomes (1104‐7, LJ19, CF413, Nara_gc5). Synteny BPs were further manually examined and annotated by mapping PacBio or nanopore long‐sequence reads against the corresponding reference genome. Visualization of syntenic relationships among genomes and subgenomic regions were aided by Circos (Krzywinski et al., 2009), JCVI python utility libraries (Tang et al., 2008), RIdeogram (Hao et al., 2020), genoPlotR (Guy et al., 2010) R packages and IGV program (Thorvaldsdóttir et al., 2013).

For genome resequencing analysis, genomic DNAs were extracted from mycelia of *C. fructicola* and *C. aenigma* isolates using the cetyltrimethylammonium bromide (CTAB) procedure, and purified genomic DNAs were sequenced with the Illumina HiSeq2500 platform (Novogene, Beijing, China) with a 100 or 150 bp pair‐ended strategy, the mean DNA insertion size for the sequencing libraries was 500 or 350 bp. Illumina sequence reads were mapped to the 1104‐7 reference genome with Bowtie2 (Langmead & Salzberg, 2012), and PacBio reads derived from Nara_gc5 and CF413 were mapped to the 1104‐7 reference genome with the long‐read mapper NGMLR (https://github.com/philres/ngmlr). SNP variants located within 1326 core fungal genes were called with BCFtools, filtered SNPs (based on QUAL, DP and MQ scores) were used for neighbour‐joining phylogenomic tree generation in MEGA 7.0 (Kumar et al., 2016). BEDtools (Quinlan & Hall, 2010) was used for calculating reads coverage of different isolates against the 1104‐7 reference genome.

### 4.4 Gene deletion and phenotypic analyses

A split‐marker‐based homologous recombination strategy was used for generating gene deletion mutants. Procedures for generating fused PCR fragments and protoplast‐mediated transformation were described previously (Liang et al., 2019). For each gene, at least two independent deletion mutants were used for phenotyping. Conidia production was induced by potato dextrose broth shake culture (Wang et al., 2017) and assays for in vitro conidial germination, appressorium differentiation and penetration were performed on cellophane membranes, whereas virulence assays were performed with detached Gala apple leaves or with Gala apple fruits collected from a local orchard. Detailed procedures for these assays were described previously (Liang et al., 2019). PCR primers used for generating gene deletion mutants are listed in Dataset S4.

A *N. benthamiana* agroinfiltration‐based cell‐death inhibition assay was applied to dissect the biological functions of predicted SSPs. Full‐length SSPs were cloned into the transient expression vector pGR107 (Wang et al., 2011), and the obtained vectors were transformed into *Agrobacterium tumefaciens* GV3101. Culturing of *A. tumefaciens* and agroinfiltration of *N. benthamiana* plants were performed as previously described (Shang et al., 2020). The pro‐apoptotic *BAX* gene was used as a positive cell death control. PCR primers used for cloning SSP sequences are listed in Dataset S4.

## CONFLICT OF INTEREST STATEMENT

The authors declare no competing interests.

## Supporting information


**DATASET S1.** Fungal strains used for genome comparisons in this study.

**DATASET S2.** Predicted genes located within GLS‐R1 and GLS‐R2 regions.

**DATASET S3.** DNA and protein sequences of GPCG17 gene based on manual annotation.

**DATASET S4.** Primer sequences used in this study.

**FIGURE S1.** Dot plot showing the genome alignment between two genome assembly versions for *Colletotrichum fructicola* 1104‐7. Matches were identified using nucmer in Mummer, forward matches are in red and reverse matches are in blue, only highly similar matches (DNA identity >99%, DNA length >10 kb) are shown. The *x*‐axis corresponded to NextDenovo‐based assembly reported in this study and the *y*‐axis corresponded to the previous assembly generated based on Canu and Flye software (Liang et al., 2020).

**FIGURE S2.** Repetitive element (RE) content in the *Colletotrichum fructicola* genomes of 1104‐7 and LJ19. (a) Cumulative RE space coverage in 1104‐7 and LJ19 genomes; (b) Boxplot showing the divergence pattern of REs from family consensus sequences; (c) relative RE space coverage variation among 1104‐7 and LJ19 chromosomes; (d) genome‐wide RIP index distribution of 1104‐7 (sampled in 10 kb slide window); (e) Boxplot showing RIP index distribution of selected RE families in 1104‐7, individual REs are shown as jittered points, the red dashed line indicates the average genome RIP index (10 kb slide window).

**FIGURE S3.** Variation in pairwise alignment coverage rate and scaffold length between selected core chromosomes and minichromosome‐like small scaffolds in CGSC. The alignment coverage rates were calculated based on genome Mummer alignment (alignment length ≥10 kb, merged if <1 kb apart).

**FIGURE S4.** Difference in sequence characteristics between core chromosomes and minichromosome‐like small scaffolds in four CGSC species. (a–c) Variations in GC content, relative TE space coverage and relative gene space coverage respectively. Scaffolds from each of the four CGSC species (represented by individual dots) are assigned into ‘Core’ and ‘Mini’ groups based on scaffold length and cross‐species conservation patterns. (d) Variation in the relative frequencies of genus‐specific genes. Genus‐specific genes were identified based on genome‐wide protein family clustering of 29 filamentous ascomycete genomes. Genes specific to individual species (SS), species complex (SCS) and genus (GS) are indicated by black, yellow and green colours respectively. (e, f) Relative frequencies of genes with gene ontology (GO) and PFAM annotations. Statistical analyses were performed with either two‐tailed *t* test (a–c) or two‐tailed Fisher’s exact test (d–f), and the corresponding *p* values are indicated.

**FIGURE S5.** Relative frequencies of genes with different virulence functions between ‘Core’ and ‘Mini’ chromosome groups in four CGSC species. Statistical analyses were performed with two‐tailed Fisher’s exact test, and the corresponding *p* values are indicated.

**FIGURE S6.** The *Colletotrichum fructicola* minichromosomes (S11, S12) evolve more rapidly than the core chromosomes (S1–S10). (a) Chromosome‐wide alignment coverage rate (in percentage) variation of different *C. fructicola* isolates relative to the 1104‐7 reference. (b) Average alignment block length variation (in bp) of different *C. fructicola* isolates relative to the 1104‐7 reference. (c) Structural variation among homologous chromosomes visualized by Genoplot.

**FIGURE S7.** Schematic representation of inversion 1 occurring in LJ19. The inversion has a length of 19.6 kb and the left and right ends are flanked by inverted insertions of a c. 1800 bp transposable element (TE). The two TE copies belong to TIR (TcMar‐Fot1), are full length (containing an intact ORF encoding DDE transposase and two terminal repeats) and are highly similar (98.77% nucleotide identity). Both synteny breakpoints (BPs) are intergenic. (a) Genoplot view of local DNA synteny, red arrowheads in LJ19 indicates TcMar‐Fot1 TEs and the inverted region is highlighted by dashed line box. (b) Schematic representation of the inversion event and IGV browser showing long‐read mapping of different strains against the LJ19 reference genome at the BP sites.

**FIGURE S8.** Schematic representation of inversion 2 occurring in 1104‐7. The inversion has a length of 0.58 Mb and no repeat element was associated with the two breakpoints (BPs). The inversion caused the split of a putative gene encoding MFS transporter (green arrowhead in panel b). (a) Genoplot visualization of macrosynteny; (b) schematic representation of the effect of DNA inversion on gene function; (c) IGV browser showing long reads mapping of different strains against the 1104‐7 reference genome at the BP sites.

**FIGURE S9.** Schematic representation of inversion 3 occurring in LJ19. The inversion has a length of 40.5 kb, the breakpoints (BPs) are complex. Both BPs are intergenic, and the LBP is flanked by LINE/TAD1 TE elements. In addition, the LBP involves two DNA losses (c. 870 bp and c. 1900 bp), one DNA inversion (c. 1800 bp) and one DNA insertion (c. 3900 bp), whereas the RBP involves a DNA insertion (c. 790 bp). (a) Genoplot of local DNA synteny, red and green arrows correspond to transposable elements; (b) schematic representation of DNA rearrangements at BP sites; (c) long‐read mapping of different strains against the LJ19 reference genome at the BP sites.

**FIGURE S10.** Schematic representation of inversion 4 occurring in LJ19. The inversion has a length of 2.94 Mb and the left and right ends are flanked by inverted insertions of a c. 5700 bp transposable element (TE). The TEs belong to LINE/Tad1, and are incomplete (containing a pseudogene encoding non‐LTR reverse transcriptase) and highly similar (98.54% nucleotide identity). Both breakpoints (BPs) are intragenic. (a) Genoplot view of local DNA synteny, red arrowheads indicate TEs. (b) Schematic representation of the inversion event, note the disruption of both gene structures in LJ19 relative to 1104‐7. (c) Long‐read mapping of different strains against the LJ19 reference genome at the BP sites.

**FIGURE S11.** Schematic representation of inversion 5 occurring in Nara_gc5. The inversion has a length of 99.4 kb and the left and right ends are neighboured by inverted insertions of a c. 5530 bp transposable element (TE). The two TE copies belong to Gypsy LTR, are complete (containing direct repeats and intact ORF encoding a reverse transcriptase) and highly similar (99.96% nucleotide identity). Both breakpoints (BPs) are intergenic. (a) Genoplot view of local DNA synteny, red arrowheads indicate TEs. (b) Schematic representation of the inversion event and long‐read mapping of different strains against the Nara_gc5 reference genome at the BP sites.

**FIGURE S12.** Schematic representation of translocation event 1 occurring in Nara_gc5. The LBP and RBP sites in 1104‐7 are both intragenic, therefore the translocation event disrupts both genes. (a) Schematic representation of the corresponding chromosomes in 1104‐7 and Nara_gc5. (b) Long‐read mapping at the synteny breaking points in 1104‐7 and Nara_gc5.

**FIGURE S13.** Schematic representation of translocation event 2 occurring in Nara_gc5. The LBP and RBP sites in 1104‐7 are both intragenic. The translocation involves a chromosome split of 1104‐7 S5 (0.92 and 4.0 Mb respectively) and the fusion of split fragments with lineage‐specific DNAs (1.85 and 0.4 Mb) respectively. (a) Schematic representation of the corresponding chromosomes in 1104‐7 and Nara_gc5. (b) Circos plot showing the lineage specificity of the corresponding chromosomes. Tracks from outside to inside represent chromosomes, DNA coverage ratios of different isolates (1104‐7, LJ19, CF413, Nara_gc5) against reference chromosome in a 10‐kb slide window, the values are calculated based on Mummer alignment and are represented as heatmaps (dense colour indicates low coverage) and links between highly similar DNA regions (length >10 kb, identity >99%) identified by local Blast search. Arrowheads point to two DNA regions specific to the Nara_gc5 isolate. (c) Long reads mapping at the synteny breaking points in 1104‐7 and Nara_gc5.

**FIGURE S14.** Schematic representation of translocation event 3 occurring in Nara_gc5. The translocation involves large DNA insertions (0.52 and 0.46 Mb) at the synteny breakpoints in Nara_gc5. The inserted DNA fragments are lineage specific, the LBP and RBP both occur in intergenic regions. (a) Schematic representation of the corresponding chromosomes in 1104‐7 and Nara_gc5. (b) Circos plot showing the lineage specificity of the corresponding chromosomes, the lineage‐specific DNAs inserted at the breakpoints (BPs) in Nara_gc5 are indicated by green boxes. Tracks from outside to inside represent chromosomes, DNA coverage ratios of different isolates (1104‐7, LJ19, CF413, Nara_gc5) against reference chromosome in a 10‐kb slide window, the values are calculated based on Mummer alignment and are represented as heatmaps (dense colour indicates low coverage) and links between highly similar DNA regions (length >10 kb, identity >99%) identified by local Blast search; (c) long‐read mapping at the synteny breaking points in 1104‐7 and Nara_gc5.

**FIGURE S15.** Isolates selected for genome resequencing and comparative analysis. (a) Neighbour‐joining phylogenetic tree constructed with 21,741 parsimony informative single‐nucleotide polymorphism (SNP) sites located within 1326 single‐copy ascomycete BUSCO genes. Numbers at nodes indicate bootstrap values based on 1000 replicates. (b) Representative photos showing disease development outcomes for indicated isolates upon artificial inoculation. Numbers within parenthesis indicate the number of leaves showing Glomerella leaf spot symptoms and the total inoculated leaves. Conidial suspensions (10^7^ spores/mL) were drop inoculated on the upper surface of healthy and detached apple leaves and the leaves were photographed at 4 days post‐inoculation. More complete information of the isolates (host and geographic origin) is shown in Dataset S1.

**FIGURE S16.** Genome‐wide DNA presence–absence polymorphism among *Colletotrichum fructicola* and *C. aenigma* isolates. Illumina or PacBio reads derived from 19 *C. fructicola* isolates and three *C. aenigma* isolates were mapped against the 1104‐7 reference genome, fraction of 1104‐7 DNA lacking reads coverage was calculated for each 10 kb window and used for heatmap generation. Tracks from outside to inside represent assembled scaffolds, histograms of gene density and repetitive element density and read coverage heatmaps. For heatmap tracks, grey (1–19) represents *C. fructicola*, whereas light blue (20–22) represents *C. aenigma*; note that light colour indicates a high fraction of DNA coverage. Colour of the track number indicates the outcome of Glomerella leaf spot (GLS) pathogenicity assays, brown indicates GLS pathogenic, green indicates non‐pathogenic and black indicates not assessed. Note the two regions on scaffold 1 showing trans‐species conservation among GLS pathogenic isolates (GLS‐R1 and GLS‐R2, highlighted in green). Links inside the tracks indicate repetitive DNA fragments (>10 kb, >90% identity) identified by self‐BlastN search, yellow ribbons link repetitive DNAs within GLS‐specific regions, whereas black ribbons link repetitive DNAs within the rest of the genome. Isolates for the heatmap tracks are as follows: 1, 1104‐7; 2, AL1‐02B; 3, AL1‐04B; 4, PGYGH01; 5, LC03680; 6, LC3155; 7, Cf413; 8, YTQS04; 9, PGRS02; 10, Nara_gc5; 11, LZLQ01; 12, LJ19; 13, LC0557; 14, LC0558; 15, LC03674; 16, LC0966; 17, LC0510; 18, LC0146; 19, LC0876; 20, XY15; 21, Cg56; 22, PC‐WS‐1. Information on isolate origin, phylogenetic position and GLS pathogenicity phenotype are detailed in Dataset S1 and Figure S15.

**FIGURE S17.** Generation and verification of gene deletion mutants for GPCGs putatively regulating Glomerella leaf spot pathogenicity. (a) Schematic representation of the gene deletion strategy based on homologous recombination, primers used for mutant detection are indicated. (b) Partial gene deletion strategy for GPCG17, which is large in size. (c) PCR identification of gene deletion mutants based on three pairs of detection primers. W indicates wild‐type control, N indicates no‐template control, Arabic number indicates designation for gene deletion strain.

**FIGURE S18.** Relative gene expression of *GPCG1*, *GPCG16* and *GPCG17* determined by reverse transcription–quantitative PCR. Fungal tissues included conidia (CON), in vitro‐grown mycelia (MYC, potato dextrose broth shake culture, 4 days), appressoria (APP) and infected apple fruit tissue sampled at 5 days after wounded inoculation (FRU) and infected apple leaves sampled at different time points (IL). Relative expression quantifications were calculated relative to CON. Error bar indicates standard deviation based on three independent technical replicates.

**FIGURE S19.** Gene deletion effects of *GPCG1*, *GPCG16* and *GPCG17* on fungal mycelial growth, perithecial development and in vitro appressorium development. (a) Potato dextrose agar colony morphology at 5 days. (b) Perithecium development of different strains on oatmeal agar at 7 days, scale bar = 500 μm. (c) In vitro development of appressoria and infectious hyphae on cellophane at 24 h post‐inoculation, scale bar = 20 μm. APP, appressorium; CON, conidium; GT, germ tube; IH, infectious hyphae within cellophane.

**FIGURE S20.** Histological visualization of fungal infectious hyphae development. Infected apple leaves were sampled at 96 h post‐inoculation, and subjected to tissue clearing, fixation and FITC‐WGA staining, and observed under fluorescent microscopy. Note the obvious mesophyll cell necrosis and infectious hyphae (IH) differentiation in the WT (1104‐6) infection, but not the mutant infections. Scale bar = 100 μm.

**FIGURE S21.** Functional and evolutionary characteristics of *GPCG1*, *GPCG16* and *GPCG17* genes. (a) Schematic representation of the relative location of *GPCG1*, *GPCG16* and *GPCG17* on chromosome 1 and the domain structures of the corresponding encoded proteins. (b) Maximum‐likelihood phylogenetic trees constructed with GPCGs (red) and their best NCBI Blast hits in *Colletotrichum* (light blue) and non‐*Colletotrichum* (light green) species. For GPCG1 and GPCG16, full‐length proteins were used for tree construction. For GPCG17, sequences corresponding the third condensation domain were used for tree construction. Best amino acid substitution models for individual protein alignments were determined by Prottest, which were LG + I + G, JTT + I + G and JTT + I + G for GPCG1, GPCG16 and GPCG17 respectively. Phylogenetic trees are mid‐rooted and numbers at node indicate bootstrap value based on 1000 replicates, low‐supported values (<70) are in grey.

**FIGURE S22.** Schematic representation of the GLS‐R1 and GLS‐R2 insertion events in 1104‐7. Note the 6‐bp direct repeats in 1104‐7 between the left and right synteny breakpoints for GLS‐R1 and GLS‐R2. For 1104‐7, IGV long‐read mapping outcome for reads derived from four *Colletotrichum fructicola* isolates are shown on the right. Synteny breakpoints are indicated as triangles.

**TABLE S1.** Genome assembly statistics of *Colletotrichum fructicola* 1104‐7 and LJ19.

**TABLE S2.** Presence of telomeric repeats at the scaffold ends.

**TABLE S3.** Annotation of isolate‐specific genomic rearrangement events on core chromosomes of the three *Colletotrichum fructicola* assemblies (CF413, LJ19, Nara_gc5) relative to the 1104‐7 reference.

**TABLE S4.** Clusters of *Colletotrichum fructicola* secondary metabolite (SM) genes showing homology to fungal SM clusters producing known products.

**TABLE S5.** Summary statistics of genome sequencing and reads mapping outcome for *Colletotrichum fructicola* and *C. aenigma* isolates against the 1104‐7 reference genome.

**TABLE S6.** PFAM functional enrichment of the 208 variable genes located within GLS‐R1 and GLS‐R2 regions.

**TABLE S7.** PFAM functional enrichment of the 76 GLS‐specific genes located within GLS‐R1 and GLS‐R2 regions.

**TABLE S8.** PFAM functional enrichment of the 49 GLS‐associated genes located within GLS‐R1 and GLS‐R2 regions.

## Data Availability

NGS and nanopore reads generated in this study are available at the National Genomics Data Center Genome Sequence Archive. Accession numbers are in the Supporting Information of this article (listed in Table S1).
