# Supplementary material for: Genome comparisons reveal accessory genes crucial for the evolution of apple Glomerella leaf spot pathogenicity in Colletotrichum fungi
Source: Mol Plant Pathol. 2024 Apr 15;25(4):e13454. doi: 10.1111/mpp.13454 (PMC11018114; doi:10.1111/mpp.13454)
Supplement: Supplementary file 5 — FIGURE S1. Dot plot showing the genome alignment between two genome assembly versions for Colletotrichum fructicola 1104‐7. Matches were identified using nucmer in Mummer, forward matches are in red and reverse matches are in blue, only highly similar matches (DNA identity >99%, DNA length >10 kb) are shown. The x‐axis corresponded to NextDenovo‐based assembly reported in this study and the y‐axis corresponded to the previous assembly generated based on Canu and Flye software (Liang et al., 2020). [file MPP-25-e13454-s027.docx]

**
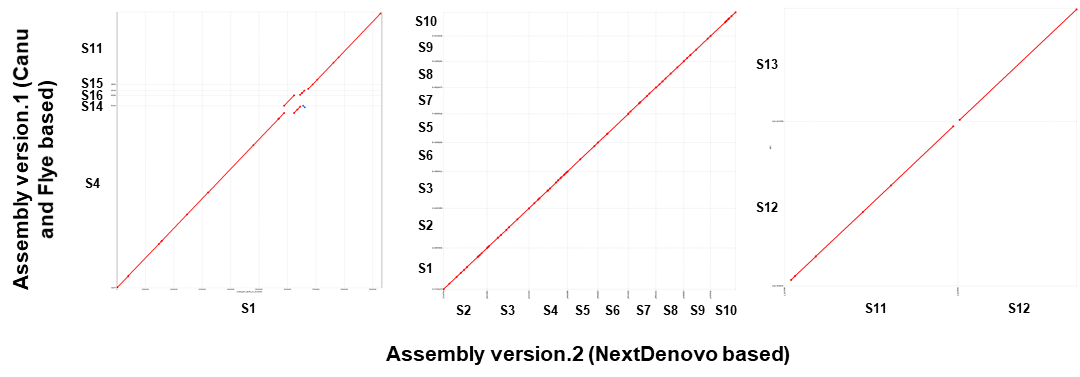
**

**Fig. S1** Dotplot showing the genome alignment between two genome assembly versions for *Colletotrichum fructicola* 1104-7. Matches were identified using nucmer in Mummer, forward matches are in red and reverse matches are in blue, only highly similar matches (DNA identity > 99%, DNA length > 10kb) are shown. The x-axis corresponded to NextDenovo based assembly reported in the current study and the y-axis corresponded to the previous assembly generated based on Canu and Flye software (Liang et al., 2020).

**Liang X, Cao M, Li S, Kong Y, Rollins JA, Zhang R, Sun G. 2020.** Highly contiguous genome resource of *Colletotrichum fructicola* generated using long-read sequencing. Molecular Plant-Microbe Interactions. 33: 790-793.
