# Supplementary material for: Genome comparisons reveal accessory genes crucial for the evolution of apple Glomerella leaf spot pathogenicity in Colletotrichum fungi
Source: Mol Plant Pathol. 2024 Apr 15;25(4):e13454. doi: 10.1111/mpp.13454 (PMC11018114; doi:10.1111/mpp.13454)
Supplement: Supplementary file 6 — FIGURE S2. Repetitive element (RE) content in the Colletotrichum fructicola genomes of 1104‐7 and LJ19. (a) Cumulative RE space coverage in 1104‐7 and LJ19 genomes; (b) Boxplot showing the divergence pattern of REs from family consensus sequences; (c) relative RE space coverage variation among 1104‐7 and LJ19 chromosomes; (d) genome‐wide RIP index distribution of 1104‐7 (sampled in 10 kb slide window); (e) Boxplot showing RIP index distribution of selected RE families in 1104‐7, individual REs are shown as jittered points, the red dashed line indicates the average genome RIP index (10 kb slide window). [file MPP-25-e13454-s009.docx]

**
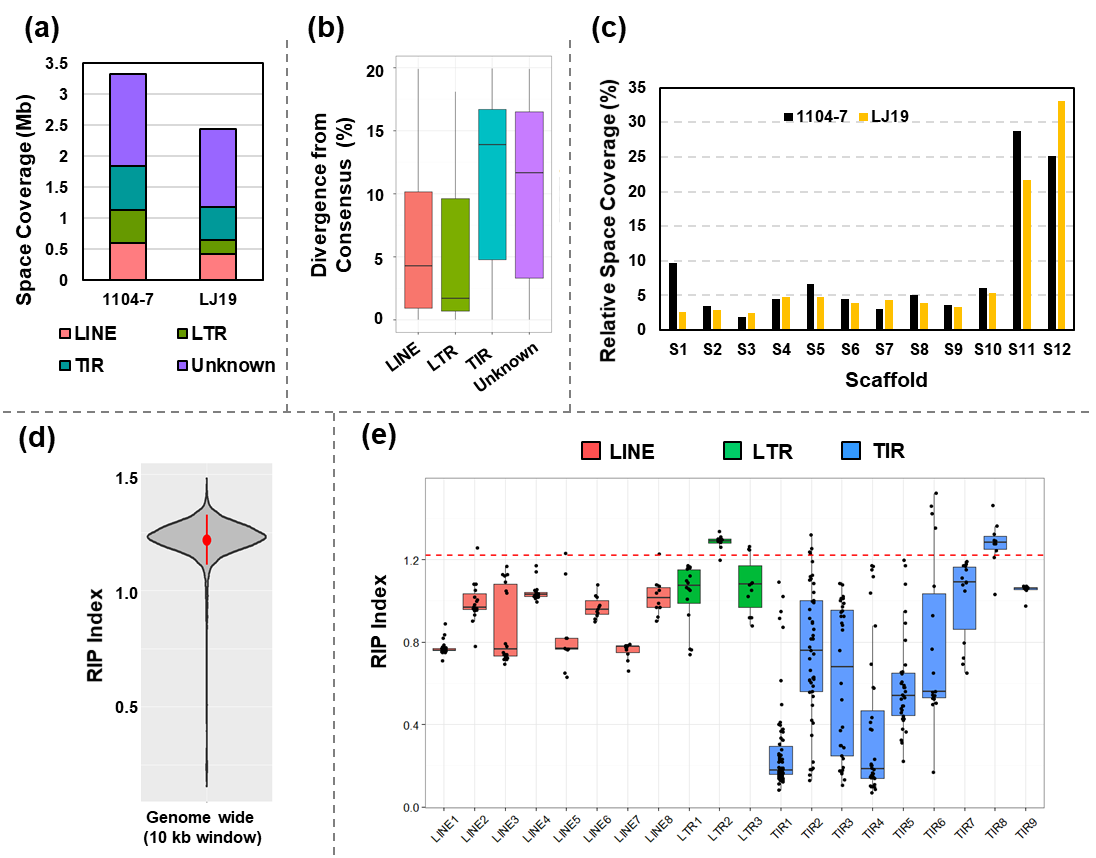
**

**Fig. S2** Repetitive element (RE) content in the *C. fructicola* genomes of 1104-7 and LJ19. (a) Cumulative RE space coverages in 1104-7 and LJ19 genomes; (b) Boxplot showing the divergence pattern of REs from family consensus sequences; (c) Relative RE space coverage variation among 1104-7 and LJ19 chromosomes; (d) Genome wide RIP index distribution of 1104-7 (sampled in 10 kb slide window); (e) Boxplot showing RIP index distribution of selected RE families in 1104-7, individual REs are shown as jittered points, the red dashed line indicates the average genome RIP index (10 kb slide window).
