# Supplementary material for: Genome comparisons reveal accessory genes crucial for the evolution of apple Glomerella leaf spot pathogenicity in Colletotrichum fungi
Source: Mol Plant Pathol. 2024 Apr 15;25(4):e13454. doi: 10.1111/mpp.13454 (PMC11018114; doi:10.1111/mpp.13454)
Supplement: Supplementary file 7 — FIGURE S3. Variation in pairwise alignment coverage rate and scaffold length between selected core chromosomes and minichromosome‐like small scaffolds in CGSC. The alignment coverage rates were calculated based on genome Mummer alignment (alignment length ≥10 kb, merged if <1 kb apart). [file MPP-25-e13454-s006.docx]

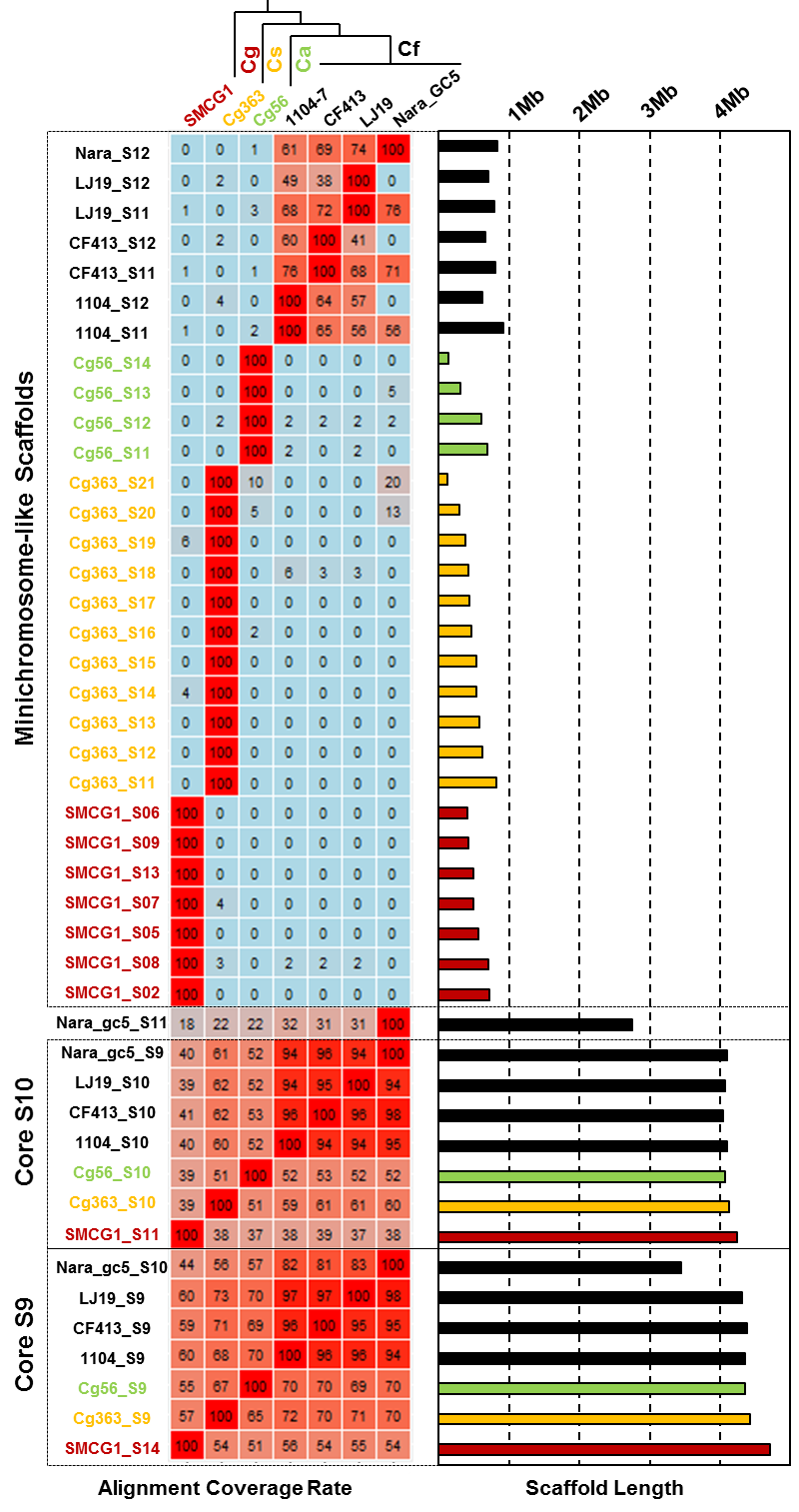


**Fig. S3** Variation in pairwise alignment coverage rate and scaffold length between selected core chromosomes and minichromosome-like small scaffolds in CGSC. The alignment coverage rates were calculated based on genome Mummer alignment (alignment length >=10kb, merged if less than 1kb apart).
