# Supplementary material for: Genome comparisons reveal accessory genes crucial for the evolution of apple Glomerella leaf spot pathogenicity in Colletotrichum fungi
Source: Mol Plant Pathol. 2024 Apr 15;25(4):e13454. doi: 10.1111/mpp.13454 (PMC11018114; doi:10.1111/mpp.13454)
Supplement: Supplementary file 8 — FIGURE S4. Difference in sequence characteristics between core chromosomes and minichromosome‐like small scaffolds in four CGSC species. (a–c) Variations in GC content, relative TE space coverage and relative gene space coverage respectively. Scaffolds from each of the four CGSC species (represented by individual dots) are assigned into ‘Core’ and ‘Mini’ groups based on scaffold length and cross‐species conservation patterns. (d) Variation in the relative frequencies of genus‐specific genes. Genus‐specific genes were identified based on genome‐wide protein family clustering of 29 filamentous ascomycete genomes. Genes specific to individual species (SS), species complex (SCS) and genus (GS) are indicated by black, yellow and green colours respectively. (e, f) Relative frequencies of genes with gene ontology (GO) and PFAM annotations. Statistical analyses were performed with either two‐tailed t test (a–c) or two‐tailed Fisher’s exact test (d–f), and the corresponding p values are indicated. [file MPP-25-e13454-s021.docx]

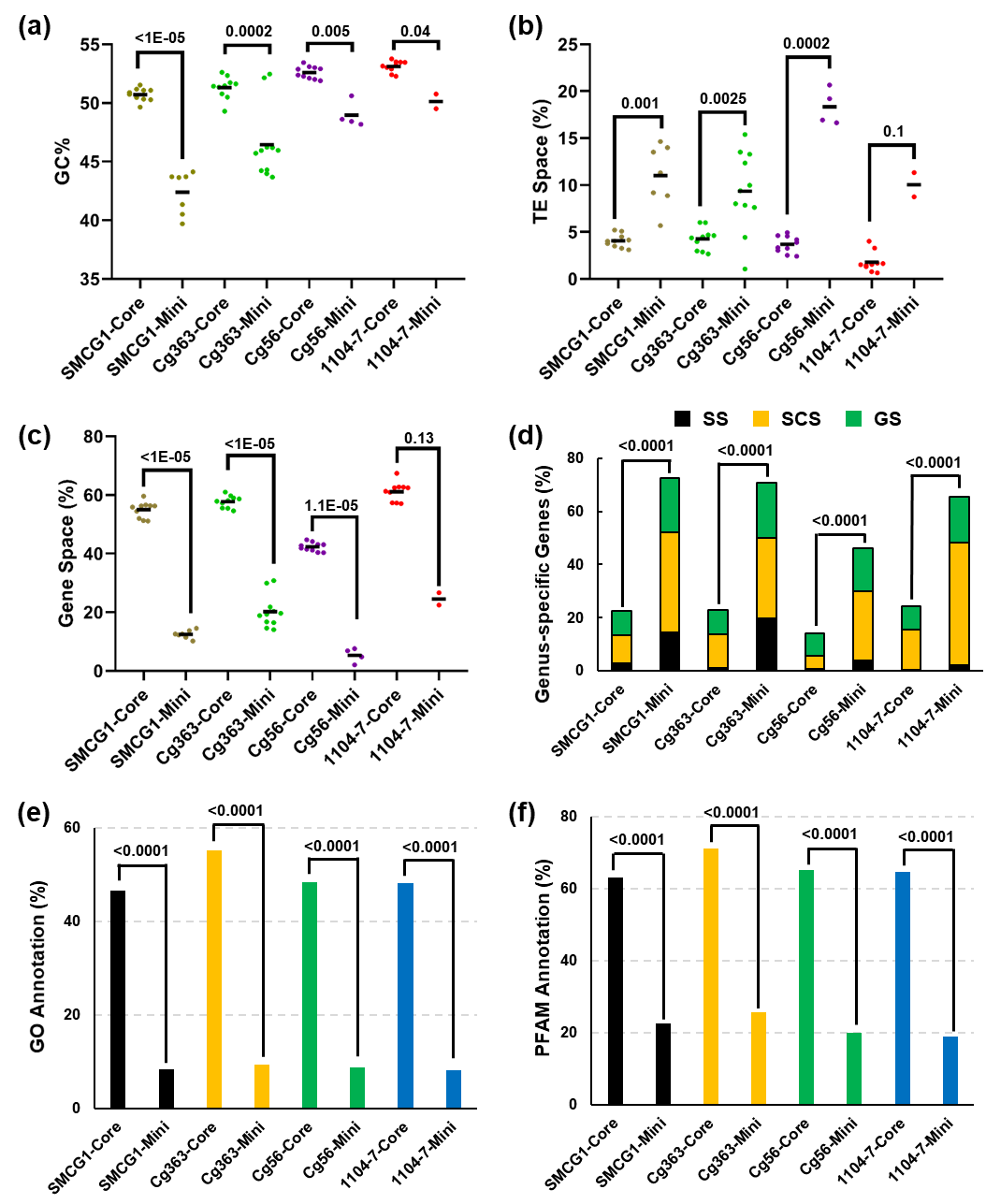


**Fig. S4** Difference in sequence characteristics between core chromosomes and minichromosome-like small scaffolds in four CGSC species. (a-c) Variations in GC content, relative TE space coverage and relative gene space coverage, respectively. Scaffolds from each of the four CGSC species (represented by individual dots) are assigned into ‘Core’ and ‘Mini’ groups based on scaffold length and cross-species conservation patterns. (d) Variation in the relative frequencies of genus-specific genes. Genus-specific genes were identified based on genome-wide protein family clustering of 29 filamentous ascomycete genomes. Genes specific to individual species (SS), species complex (SCS) and genus (GS) are indicated by black, yellow and green colors respectively. (e and f) Relative frequencies of genes with gene ontology (GO) and PFAM annotations. Statistical analyses were performed with either two-tailed T-test (a-c) or two-tailed Fisher’s exact test (d-f), and the corresponding p-values are indicated.
