# Supplementary material for: Genome comparisons reveal accessory genes crucial for the evolution of apple Glomerella leaf spot pathogenicity in Colletotrichum fungi
Source: Mol Plant Pathol. 2024 Apr 15;25(4):e13454. doi: 10.1111/mpp.13454 (PMC11018114; doi:10.1111/mpp.13454)
Supplement: Supplementary file 9 — FIGURE S5. Relative frequencies of genes with different virulence functions between ‘Core’ and ‘Mini’ chromosome groups in four CGSC species. Statistical analyses were performed with two‐tailed Fisher’s exact test, and the corresponding p values are indicated. [file MPP-25-e13454-s026.docx]

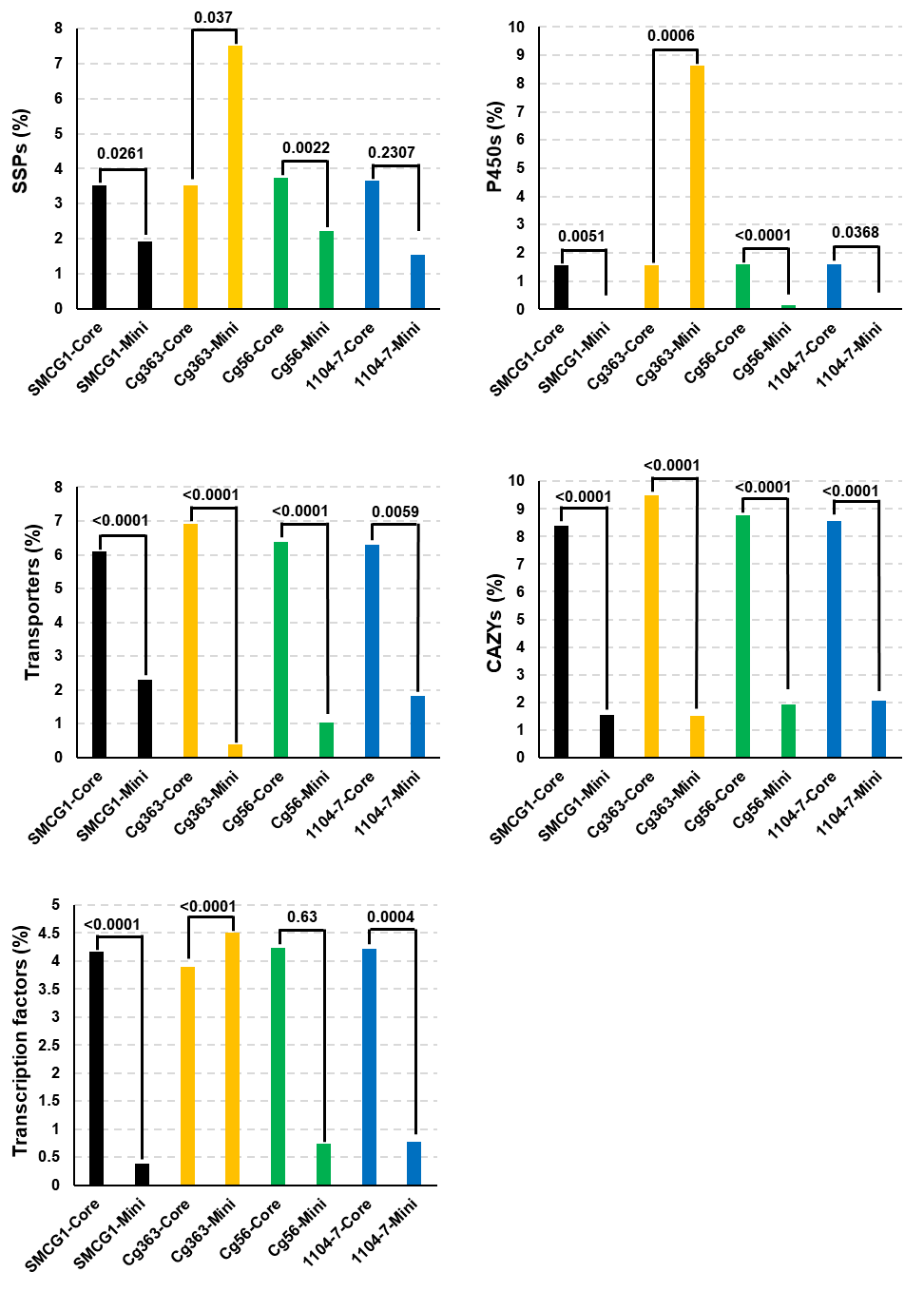


**Fig. S5** Relative frequencies of genes with different virulence functions between ‘Core’ and ‘Mini’ chromosome groups in four CGSC species. Statistical analyses were performed with two-tailed Fisher’s exact test, and the corresponding p-values are indicated.
