# Supplementary material for: Genome comparisons reveal accessory genes crucial for the evolution of apple Glomerella leaf spot pathogenicity in Colletotrichum fungi
Source: Mol Plant Pathol. 2024 Apr 15;25(4):e13454. doi: 10.1111/mpp.13454 (PMC11018114; doi:10.1111/mpp.13454)
Supplement: Supplementary file 10 — FIGURE S6. The Colletotrichum fructicola minichromosomes (S11, S12) evolve more rapidly than the core chromosomes (S1–S10). (a) Chromosome‐wide alignment coverage rate (in percentage) variation of different C. fructicola isolates relative to the 1104‐7 reference. (b) Average alignment block length variation (in bp) of different C. fructicola isolates relative to the 1104‐7 reference. (c) Structural variation among homologous chromosomes visualized by Genoplot. [file MPP-25-e13454-s033.docx]

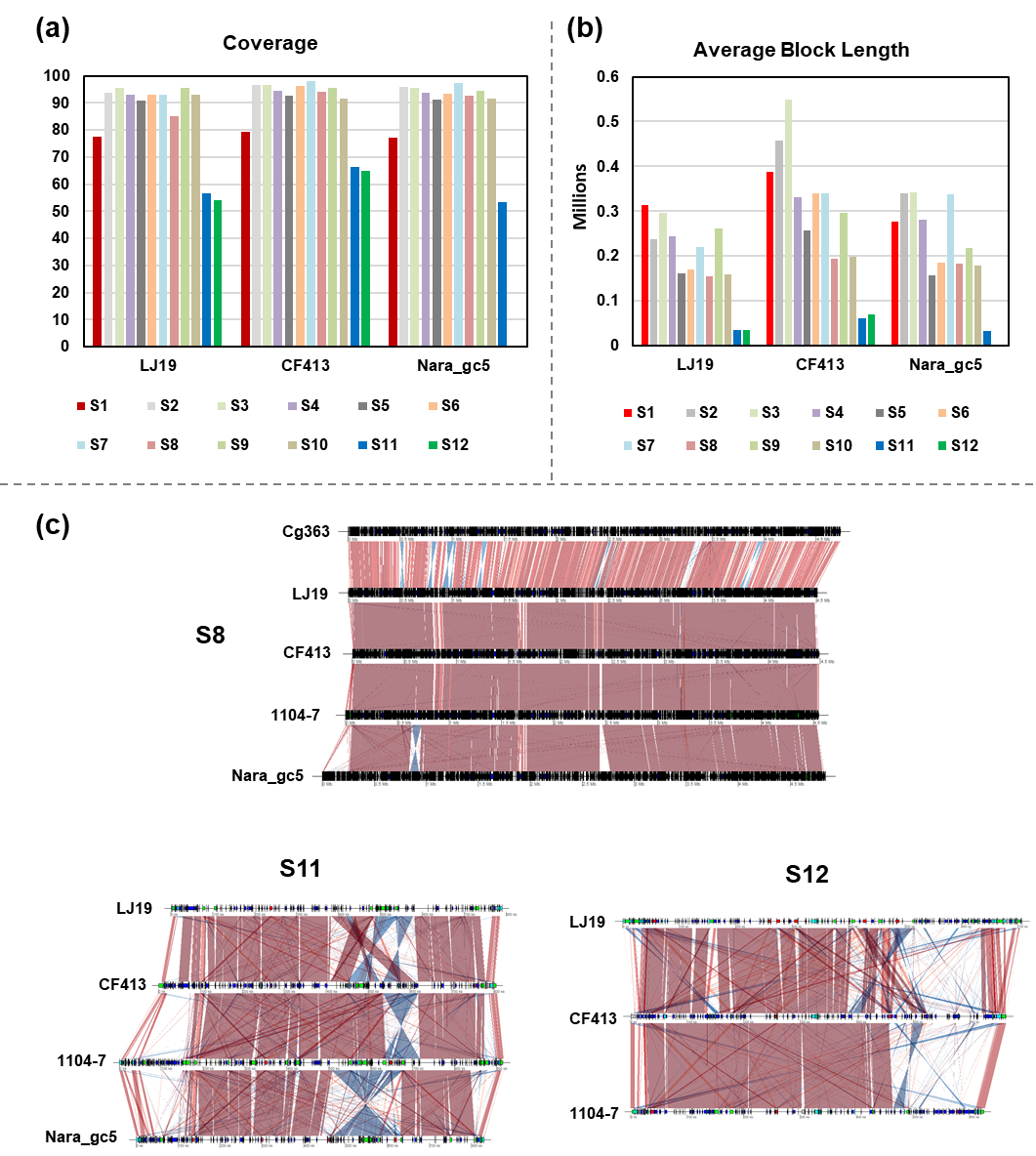


**Fig. S6** The *C. fructicola* minichromosomes (S11, S12) evolve more rapidly than the core chromosomes (S1-S10). (a) Chromosome wide alignment coverage rate (in percentage) variation of different *C. fructicola* isolates relative to the 1104-7 reference. (b) Average alignment block length variation (in bp) of different *C. fructicola* isolates relative to the 1104-7 reference. (c) Structural variation among homologous chromosomes visualized by Genoplot.
