# Supplementary material for: Genome comparisons reveal accessory genes crucial for the evolution of apple Glomerella leaf spot pathogenicity in Colletotrichum fungi
Source: Mol Plant Pathol. 2024 Apr 15;25(4):e13454. doi: 10.1111/mpp.13454 (PMC11018114; doi:10.1111/mpp.13454)
Supplement: Supplementary file 11 — FIGURE S7. Schematic representation of inversion 1 occurring in LJ19. The inversion has a length of 19.6 kb and the left and right ends are flanked by inverted insertions of a c. 1800 bp transposable element (TE). The two TE copies belong to TIR (TcMar‐Fot1), are full length (containing an intact ORF encoding DDE transposase and two terminal repeats) and are highly similar (98.77% nucleotide identity). Both synteny breakpoints (BPs) are intergenic. (a) Genoplot view of local DNA synteny, red arrowheads in LJ19 indicates TcMar‐Fot1 TEs and the inverted region is highlighted by dashed line box. (b) Schematic representation of the inversion event and IGV browser showing long‐read mapping of different strains against the LJ19 reference genome at the BP sites. [file MPP-25-e13454-s011.docx]

**
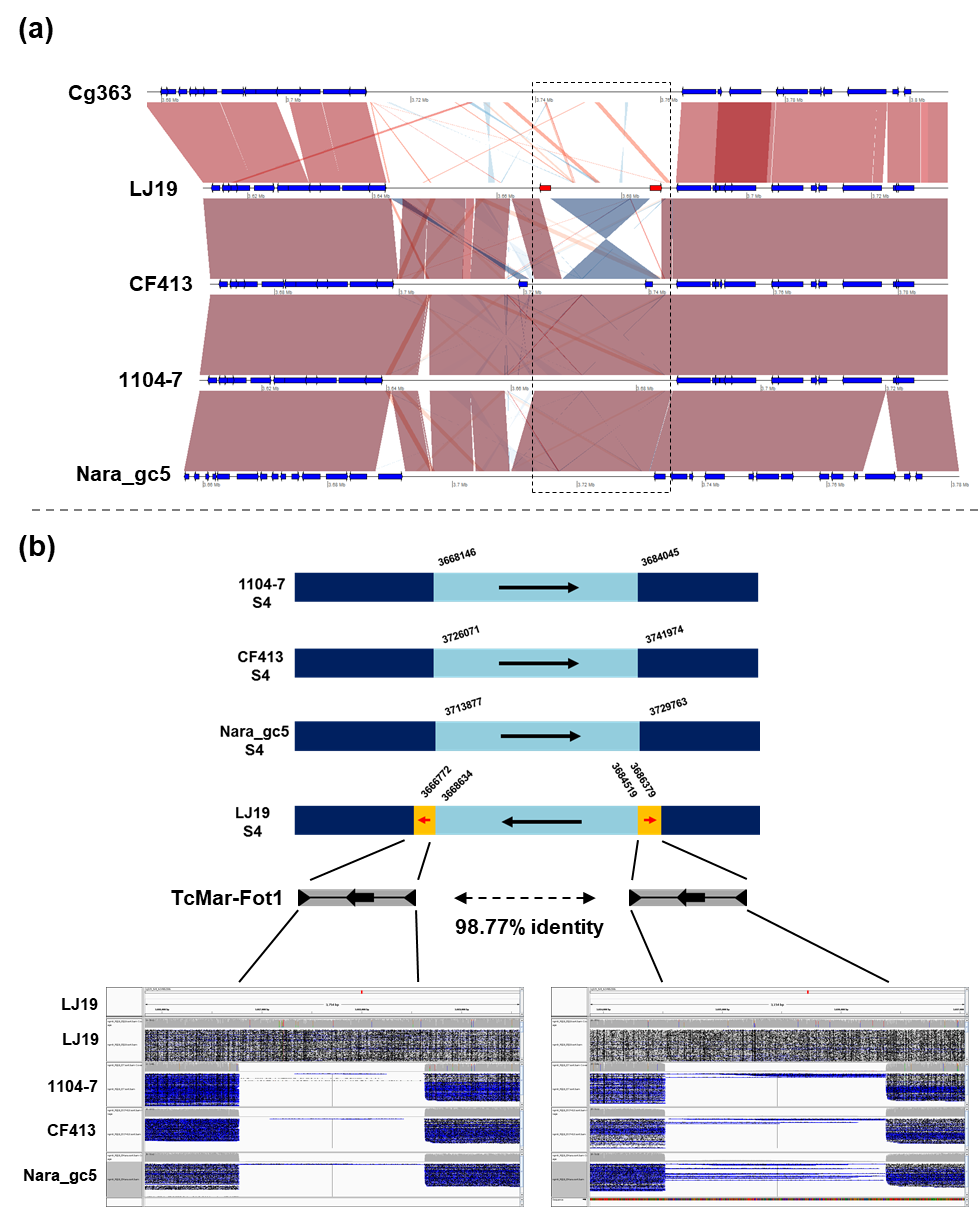
**

**Fig. S7** Schematic representation of inversion 1 occurring in LJ19. The inversion has a length of 19.6 kb and the left and right ends are flanked by inverted insertions of a ~1800 bp transposable element (TE). The two TE copies belong to TIR (TcMar-Fot1), are full length (containing an intact ORF encoding DDE transposase and two terminal repeats) and are highly similar (98.77% nucleotide identity). Both synteny break points (BPs) are intergenic. (a) Genoplot view of local DNA synteny, red arrowheads in LJ19 indicates TcMar-Fot1 TEs and the inverted region is highlighted by dashed line box. (b) Schematic representation of the inversion event and IGV browser showing long read mapping of different strains against the LJ19 reference genome at the BP sites.
