# Supplementary material for: Genome comparisons reveal accessory genes crucial for the evolution of apple Glomerella leaf spot pathogenicity in Colletotrichum fungi
Source: Mol Plant Pathol. 2024 Apr 15;25(4):e13454. doi: 10.1111/mpp.13454 (PMC11018114; doi:10.1111/mpp.13454)
Supplement: Supplementary file 12 — FIGURE S8. Schematic representation of inversion 2 occurring in 1104‐7. The inversion has a length of 0.58 Mb and no repeat element was associated with the two breakpoints (BPs). The inversion caused the split of a putative gene encoding MFS transporter (green arrowhead in panel b). (a) Genoplot visualization of macrosynteny; (b) schematic representation of the effect of DNA inversion on gene function; (c) IGV browser showing long reads mapping of different strains against the 1104‐7 reference genome at the BP sites. [file MPP-25-e13454-s022.docx]

**
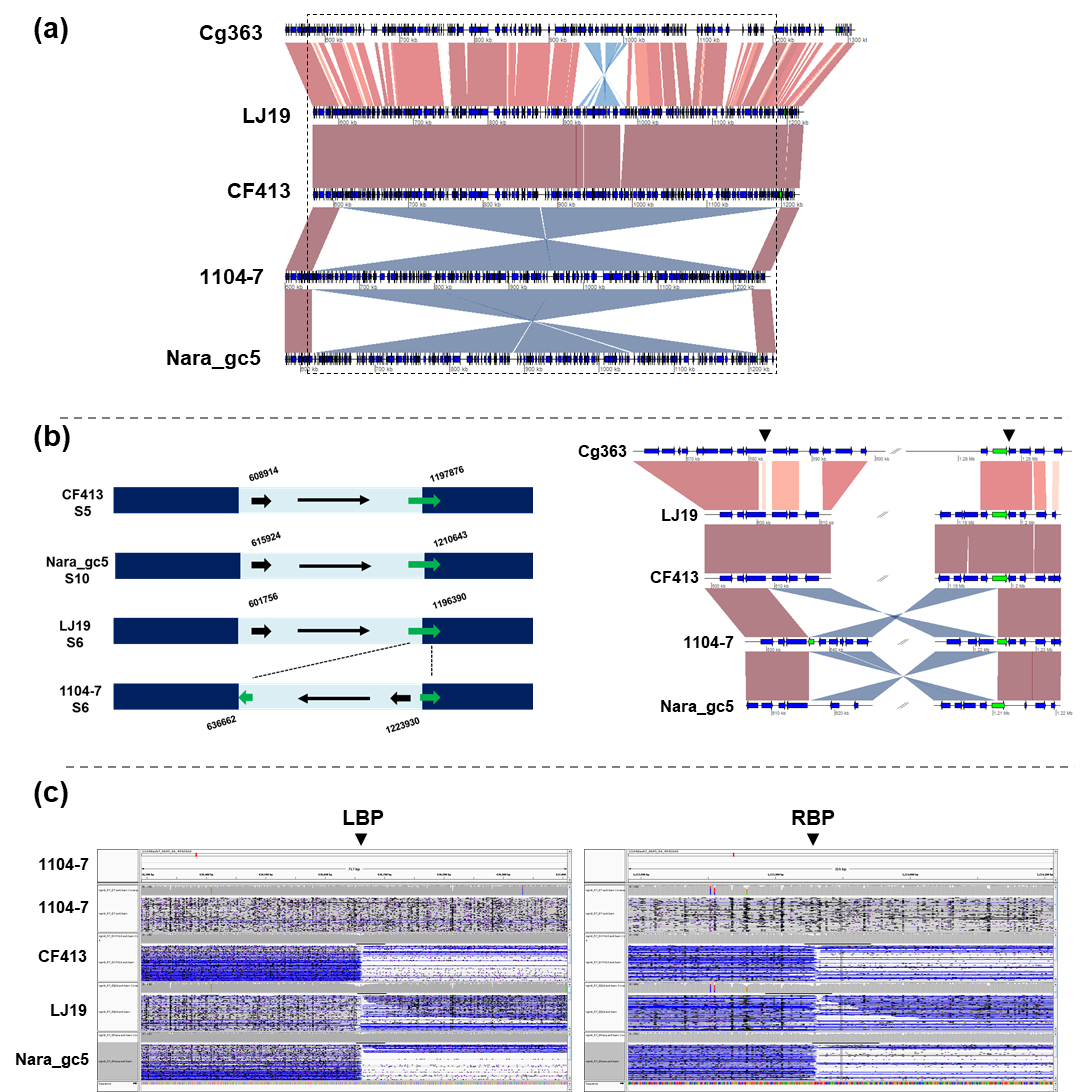
**

**Fig. S8** Schematic representation of inversion 2 occurring in 1104-7. The inversion has a length of 0.58 Mb and no repeat element was associated with the two BPs. The inversion caused the split of a putative gene encoding MFS transporter (green arrowhead in panel b). (a) Genoplot visualization of macrosynteny; (b) Schematic representation of the effect of DNA inversion on gene function; (c) IGV browser showing long reads mapping of different strains against the 1104-7 reference genome at the BP sites.
