# Supplementary material for: Genome comparisons reveal accessory genes crucial for the evolution of apple Glomerella leaf spot pathogenicity in Colletotrichum fungi
Source: Mol Plant Pathol. 2024 Apr 15;25(4):e13454. doi: 10.1111/mpp.13454 (PMC11018114; doi:10.1111/mpp.13454)
Supplement: Supplementary file 13 — FIGURE S9. Schematic representation of inversion 3 occurring in LJ19. The inversion has a length of 40.5 kb, the breakpoints (BPs) are complex. Both BPs are intergenic, and the LBP is flanked by LINE/TAD1 TE elements. In addition, the LBP involves two DNA losses (c. 870 bp and c. 1900 bp), one DNA inversion (c. 1800 bp) and one DNA insertion (c. 3900 bp), whereas the RBP involves a DNA insertion (c. 790 bp). (a) Genoplot of local DNA synteny, red and green arrows correspond to transposable elements; (b) schematic representation of DNA rearrangements at BP sites; (c) long‐read mapping of different strains against the LJ19 reference genome at the BP sites. [file MPP-25-e13454-s025.docx]

**
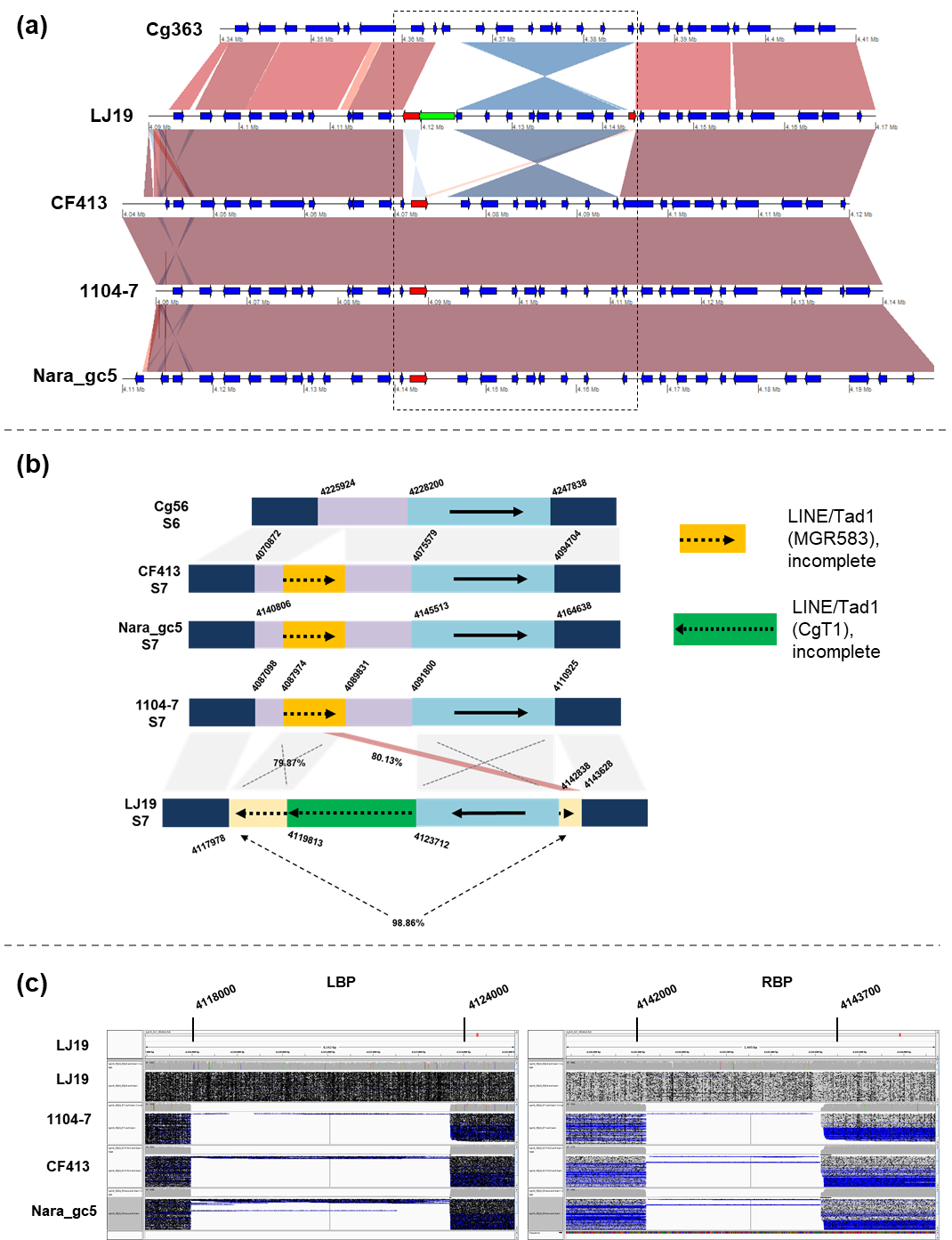
**

**Fig. S9** Schematic representation of inversion 3 occurring in LJ19. The inversion has a length of 40.5 kb, the BP break points are complex. Both BPs are intergenic, and the LBP is flanked by LINE/TAD1 TE elements. In addition, the LBP involves two DNA losses (~870 bp and ~1,900 bp), one DNA inversion (~1,800 bp), and one DNA insertion (~3,900 bp) whereas the RBP involves a DNA insertion (~ 790 bp). (a) Genoplot of local DNA synteny, red and green arrows correspond to TE elements; (b) Schematic representation of DNA rearrangements at BP sites; (c) Long read mapping of different strains against the LJ19 reference genome at the BP sites.
