# Supplementary material for: Genome comparisons reveal accessory genes crucial for the evolution of apple Glomerella leaf spot pathogenicity in Colletotrichum fungi
Source: Mol Plant Pathol. 2024 Apr 15;25(4):e13454. doi: 10.1111/mpp.13454 (PMC11018114; doi:10.1111/mpp.13454)
Supplement: Supplementary file 14 — FIGURE S10. Schematic representation of inversion 4 occurring in LJ19. The inversion has a length of 2.94 Mb and the left and right ends are flanked by inverted insertions of a c. 5700 bp transposable element (TE). The TEs belong to LINE/Tad1, and are incomplete (containing a pseudogene encoding non‐LTR reverse transcriptase) and highly similar (98.54% nucleotide identity). Both breakpoints (BPs) are intragenic. (a) Genoplot view of local DNA synteny, red arrowheads indicate TEs. (b) Schematic representation of the inversion event, note the disruption of both gene structures in LJ19 relative to 1104‐7. (c) Long‐read mapping of different strains against the LJ19 reference genome at the BP sites. [file MPP-25-e13454-s012.docx]

**
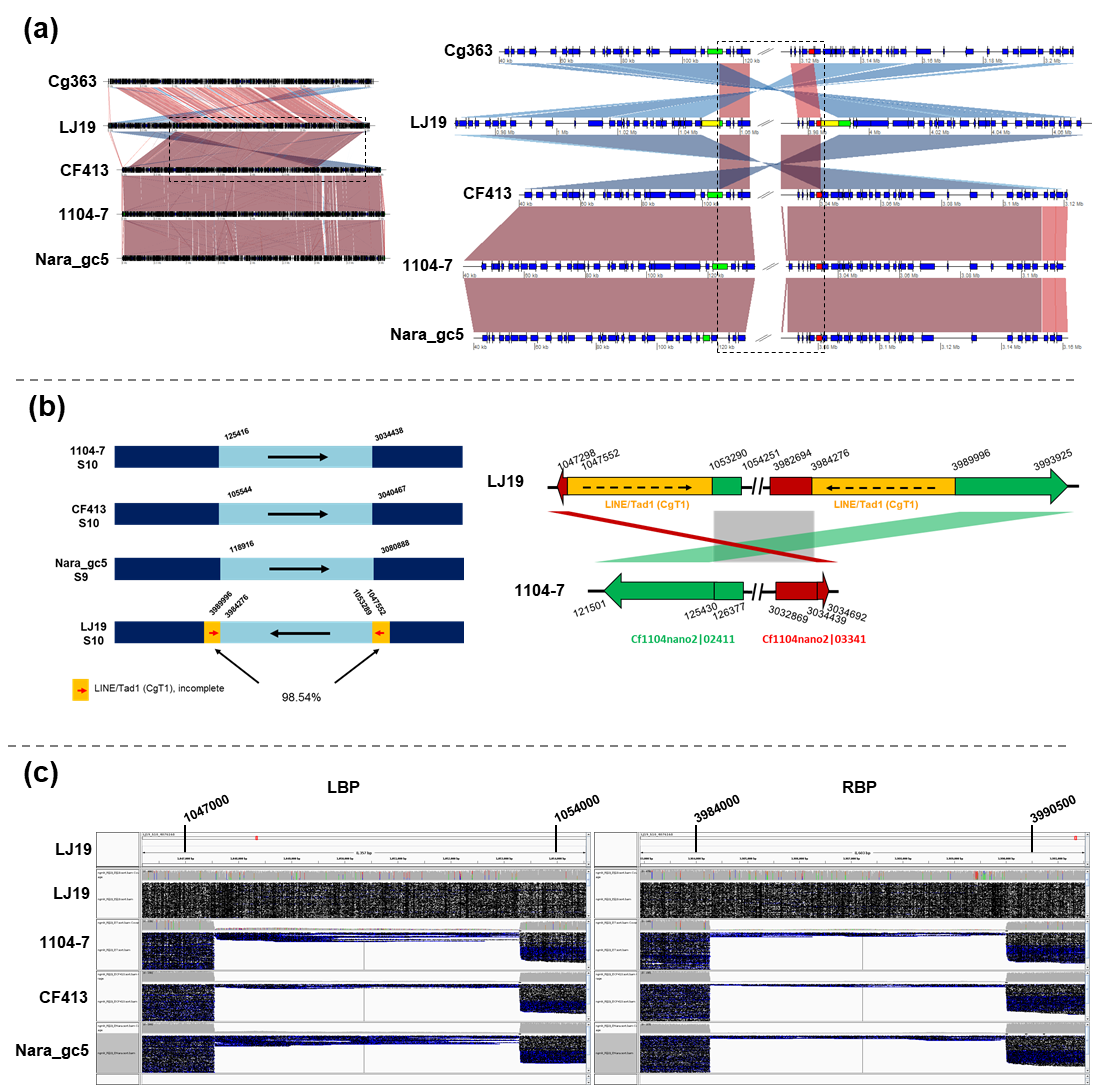
**

**Fig. S10** Schematic representation of inversion 4 occurring in LJ19. The inversion has a length of 2.94 Mb and the left and right ends are flanked by inverted insertions of a ~ 5,700 bp TE. The TEs belong to LINE/Tad1, and are incomplete (containing a pseudogene encoding non-LTR reverse transcriptase) and highly similar (98.54% nucleotide identity). Both BPs are intragenic. (a) Genoplot view of local DNA synteny, red arrowheads indicate TE elements. (b) Schematic representation of the inversion event, note the disruption of both gene structures in LJ19 relative to 1104-7; (c) Long read mapping of different strains against the LJ19 reference genome at the BP sites.
