# Supplementary material for: Genome comparisons reveal accessory genes crucial for the evolution of apple Glomerella leaf spot pathogenicity in Colletotrichum fungi
Source: Mol Plant Pathol. 2024 Apr 15;25(4):e13454. doi: 10.1111/mpp.13454 (PMC11018114; doi:10.1111/mpp.13454)
Supplement: Supplementary file 15 — FIGURE S11. Schematic representation of inversion 5 occurring in Nara_gc5. The inversion has a length of 99.4 kb and the left and right ends are neighboured by inverted insertions of a c. 5530 bp transposable element (TE). The two TE copies belong to Gypsy LTR, are complete (containing direct repeats and intact ORF encoding a reverse transcriptase) and highly similar (99.96% nucleotide identity). Both breakpoints (BPs) are intergenic. (a) Genoplot view of local DNA synteny, red arrowheads indicate TEs. (b) Schematic representation of the inversion event and long‐read mapping of different strains against the Nara_gc5 reference genome at the BP sites. [file MPP-25-e13454-s004.docx]

**
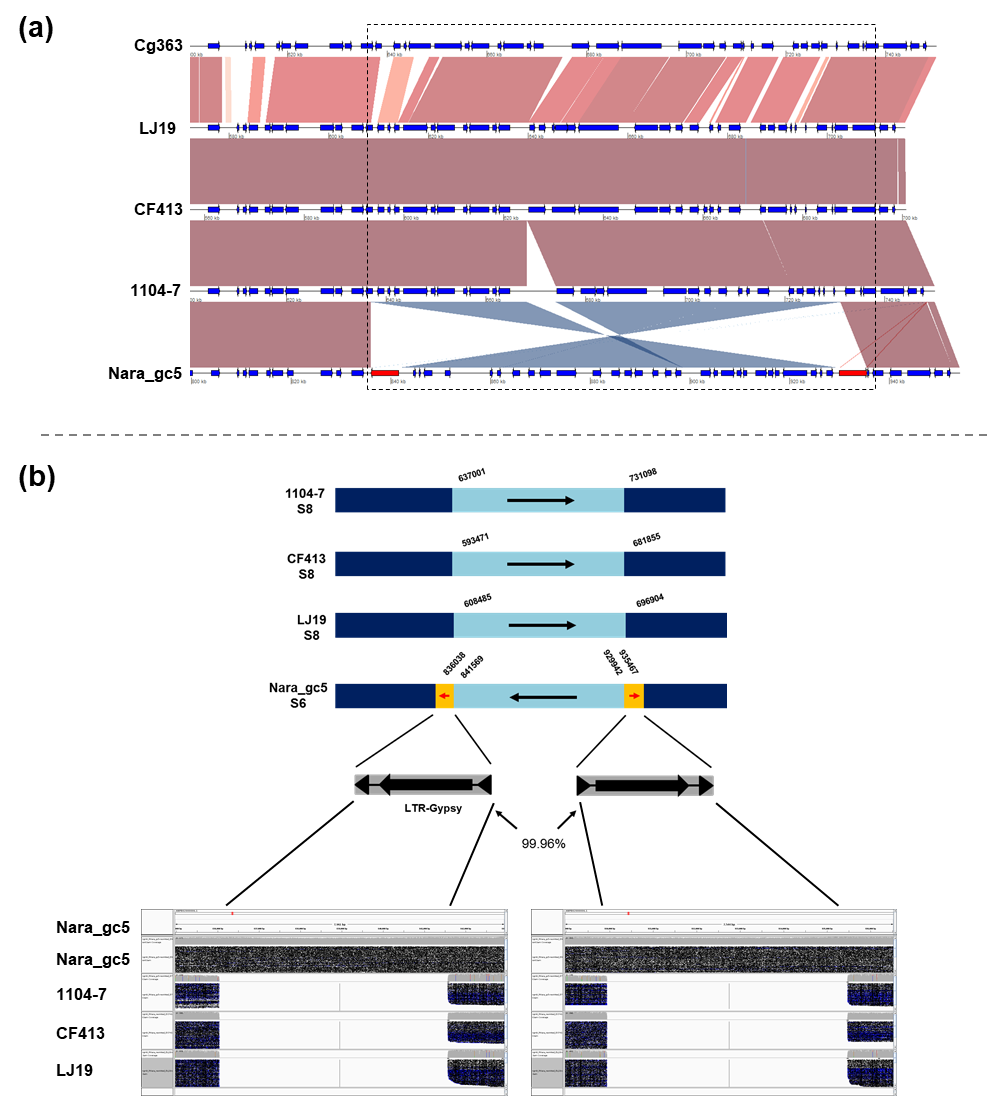
**

**Fig. S11** Schematic representation of inversion 5 occurring in Nara_gc5. The inversion has a length of 99.4 kb and the left and right ends are neighbored by inverted insertions of a ~ 5,530 bp TE. The two TE copies belong to Gypsy LTR, are complete (containing direct repeats and intact ORF encoding a reverse transcriptase) and highly similar (99.96% nucleotide identity). Both BPs are intergenic. (a) Genoplot view of local DNA synteny, red arrowheads indicate TE elements. (b) Schematic representation of the inversion event and long read mapping of different strains against the Nara_gc5 reference genome at the BP sites.
