# Supplementary material for: Genome comparisons reveal accessory genes crucial for the evolution of apple Glomerella leaf spot pathogenicity in Colletotrichum fungi
Source: Mol Plant Pathol. 2024 Apr 15;25(4):e13454. doi: 10.1111/mpp.13454 (PMC11018114; doi:10.1111/mpp.13454)
Supplement: Supplementary file 16 — FIGURE S12. Schematic representation of translocation event 1 occurring in Nara_gc5. The LBP and RBP sites in 1104‐7 are both intragenic, therefore the translocation event disrupts both genes. (a) Schematic representation of the corresponding chromosomes in 1104‐7 and Nara_gc5. (b) Long‐read mapping at the synteny breaking points in 1104‐7 and Nara_gc5. [file MPP-25-e13454-s032.docx]

**
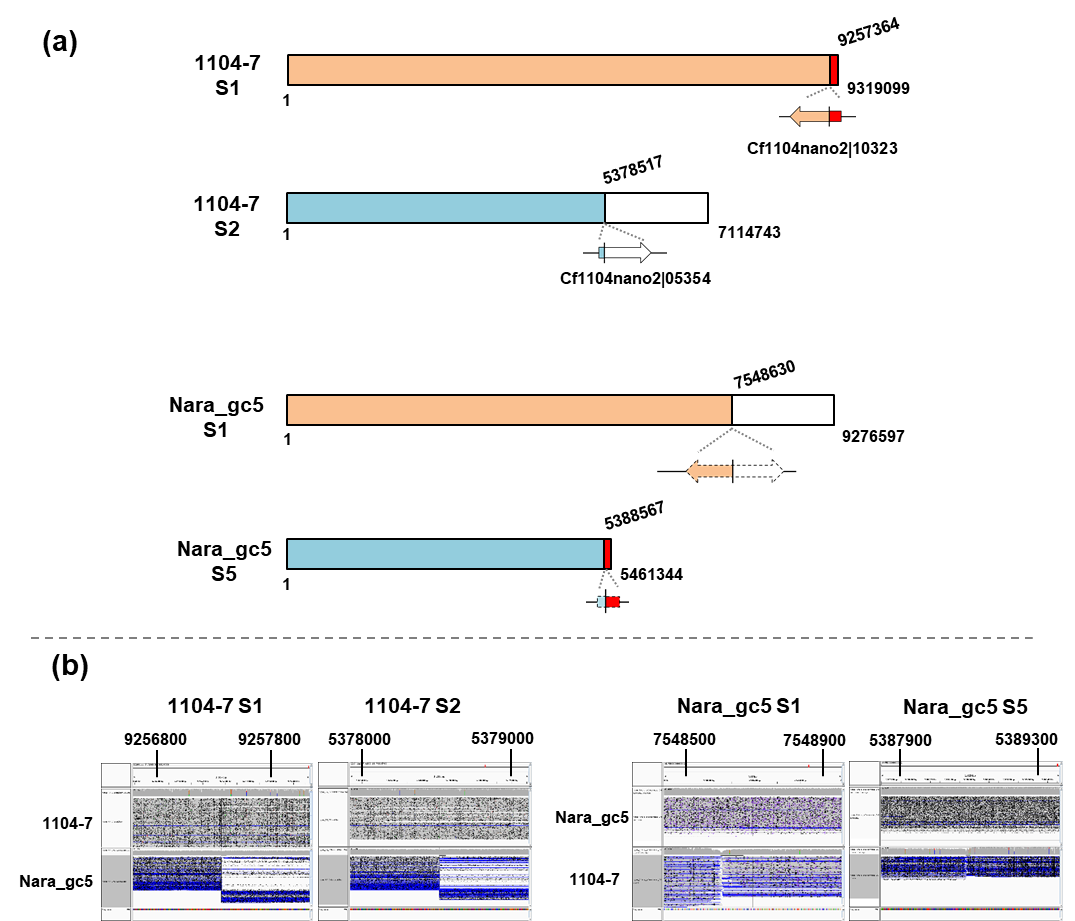
**

**Fig. S12** Schematic representation of translocation event 1 occurring in Nara_gc5. The LBP and RBP sites in 1104-7 are both intragenic, therefore the translocation event disrupts both genes. (a) Schematic representation of the corresponding chromosomes in 1104-7 and Nara_gc5; (b) Long read mapping at the synteny breaking points in 1104-7 and Nara_gc5.
