# Supplementary material for: Genome comparisons reveal accessory genes crucial for the evolution of apple Glomerella leaf spot pathogenicity in Colletotrichum fungi
Source: Mol Plant Pathol. 2024 Apr 15;25(4):e13454. doi: 10.1111/mpp.13454 (PMC11018114; doi:10.1111/mpp.13454)
Supplement: Supplementary file 17 — FIGURE S13. Schematic representation of translocation event 2 occurring in Nara_gc5. The LBP and RBP sites in 1104‐7 are both intragenic. The translocation involves a chromosome split of 1104‐7 S5 (0.92 and 4.0 Mb respectively) and the fusion of split fragments with lineage‐specific DNAs (1.85 and 0.4 Mb) respectively. (a) Schematic representation of the corresponding chromosomes in 1104‐7 and Nara_gc5. (b) Circos plot showing the lineage specificity of the corresponding chromosomes. Tracks from outside to inside represent chromosomes, DNA coverage ratios of different isolates (1104‐7, LJ19, CF413, Nara_gc5) against reference chromosome in a 10‐kb slide window, the values are calculated based on Mummer alignment and are represented as heatmaps (dense colour indicates low coverage) and links between highly similar DNA regions (length >10 kb, identity >99%) identified by local Blast search. Arrowheads point to two DNA regions specific to the Nara_gc5 isolate. (c) Long reads mapping at the synteny breaking points in 1104‐7 and Nara_gc5. [file MPP-25-e13454-s020.docx]

**
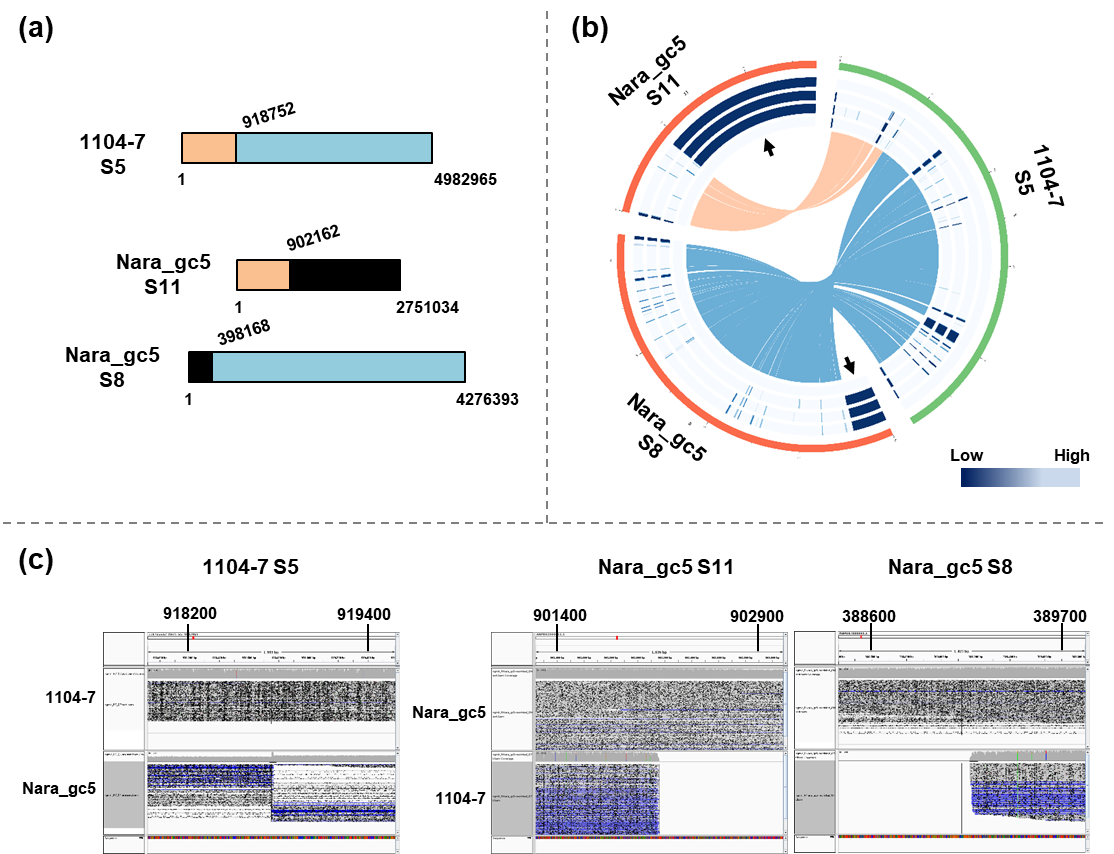
**

**Fig. S13** Schematic representation of translocation event 2 occurring in Nara_gc5. The LBP and RBP sites in 1104-7 are both intragenic. The translocation involves a chromosome split of 1104-7 S5 (0.92 Mb and 4.0 Mb respectively) and the fusion of split fragments with lineage-specific DNAs (1.85 Mb and 0.4 Mb) respectively. (a) Schematic representation of the corresponding chromosomes in 1104-7 and Nara_gc5; (b) Circos plot showing the lineage specificity of the corresponding chromosomes. Tracks from outside to inside represent chromosomes, DNA coverage ratios of different isolates (1104-7, LJ19, CF413, Nara_gc5) against reference chromosome in a 10 kb slide window, the values are calculated based on Mummer alignment and are represented as heatmaps (dense color indicates low coverage), and links between highly similar DNA regions (length > 10 kb, identity > 99%) identified by local Blast search. Arrowheads point to two DNA regions specific to the Nara_gc5 isolate; (c) Long reads mapping at the synteny breaking points in 1104-7 and Nara_gc5.
