# Supplementary material for: Genome comparisons reveal accessory genes crucial for the evolution of apple Glomerella leaf spot pathogenicity in Colletotrichum fungi
Source: Mol Plant Pathol. 2024 Apr 15;25(4):e13454. doi: 10.1111/mpp.13454 (PMC11018114; doi:10.1111/mpp.13454)
Supplement: Supplementary file 18 — FIGURE S14. Schematic representation of translocation event 3 occurring in Nara_gc5. The translocation involves large DNA insertions (0.52 and 0.46 Mb) at the synteny breakpoints in Nara_gc5. The inserted DNA fragments are lineage specific, the LBP and RBP both occur in intergenic regions. (a) Schematic representation of the corresponding chromosomes in 1104‐7 and Nara_gc5. (b) Circos plot showing the lineage specificity of the corresponding chromosomes, the lineage‐specific DNAs inserted at the breakpoints (BPs) in Nara_gc5 are indicated by green boxes. Tracks from outside to inside represent chromosomes, DNA coverage ratios of different isolates (1104‐7, LJ19, CF413, Nara_gc5) against reference chromosome in a 10‐kb slide window, the values are calculated based on Mummer alignment and are represented as heatmaps (dense colour indicates low coverage) and links between highly similar DNA regions (length >10 kb, identity >99%) identified by local Blast search; (c) long‐read mapping at the synteny breaking points in 1104‐7 and Nara_gc5. [file MPP-25-e13454-s016.docx]

**
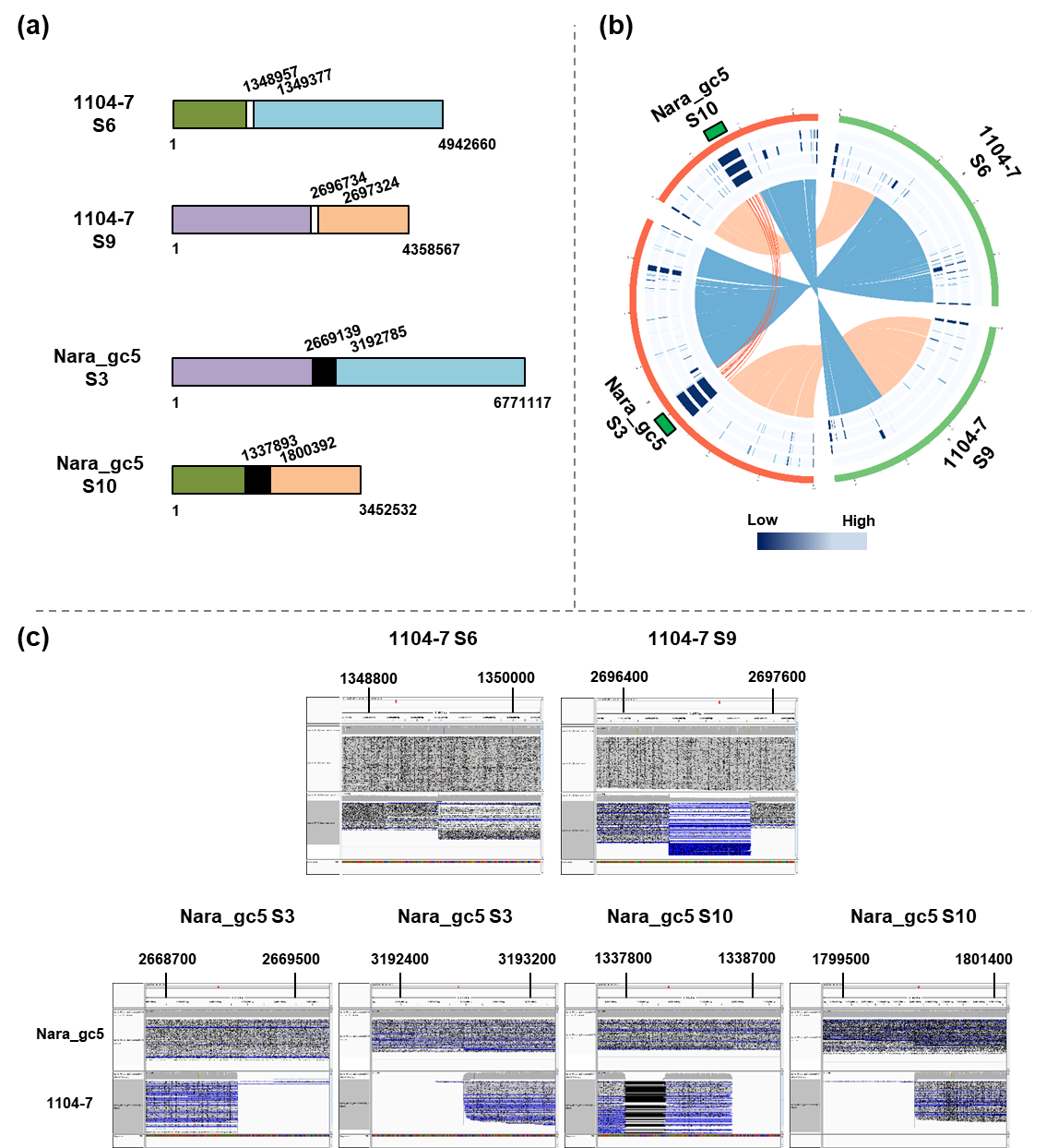
**

**Fig. S14** Schematic representation of translocation event 3 occurring in Nara_gc5. The translocation involves large DNA insertions (0.52 Mb and 0.46 Mb) at the synteny break points in Nara_gc5. The inserted DNA fragments are lineage-specific, the LBP and RBP both occur in intergenic regions. (a) Schematic representation of the corresponding chromosomes in 1104-7 and Nara_gc5; (b) Circos plot showing the lineage specificity of the corresponding chromosomes, the lineage-specific DNAs inserted at the BP points in Nara_gc5 are indicated by green boxes. Tracks from outside to inside represent chromosomes, DNA coverage ratios of different isolates (1104-7, LJ19, CF413, Nara_gc5) against reference chromosome in a 10 kb slide window, the values are calculated based on Mummer alignment and are represented as heatmaps (dense color indicates low coverage), and links between highly similar DNA regions (length > 10 kb, identity > 99%) identified by local Blast search; (c) Long read mapping at the synteny breaking points in 1104-7 and Nara_gc5.
