# Supplementary material for: Genome comparisons reveal accessory genes crucial for the evolution of apple Glomerella leaf spot pathogenicity in Colletotrichum fungi
Source: Mol Plant Pathol. 2024 Apr 15;25(4):e13454. doi: 10.1111/mpp.13454 (PMC11018114; doi:10.1111/mpp.13454)
Supplement: Supplementary file 19 — FIGURE S15. Isolates selected for genome resequencing and comparative analysis. (a) Neighbour‐joining phylogenetic tree constructed with 21,741 parsimony informative single‐nucleotide polymorphism (SNP) sites located within 1326 single‐copy ascomycete BUSCO genes. Numbers at nodes indicate bootstrap values based on 1000 replicates. (b) Representative photos showing disease development outcomes for indicated isolates upon artificial inoculation. Numbers within parenthesis indicate the number of leaves showing Glomerella leaf spot symptoms and the total inoculated leaves. Conidial suspensions (107 spores/mL) were drop inoculated on the upper surface of healthy and detached apple leaves and the leaves were photographed at 4 days post‐inoculation. More complete information of the isolates (host and geographic origin) is shown in Dataset S1. [file MPP-25-e13454-s002.docx]

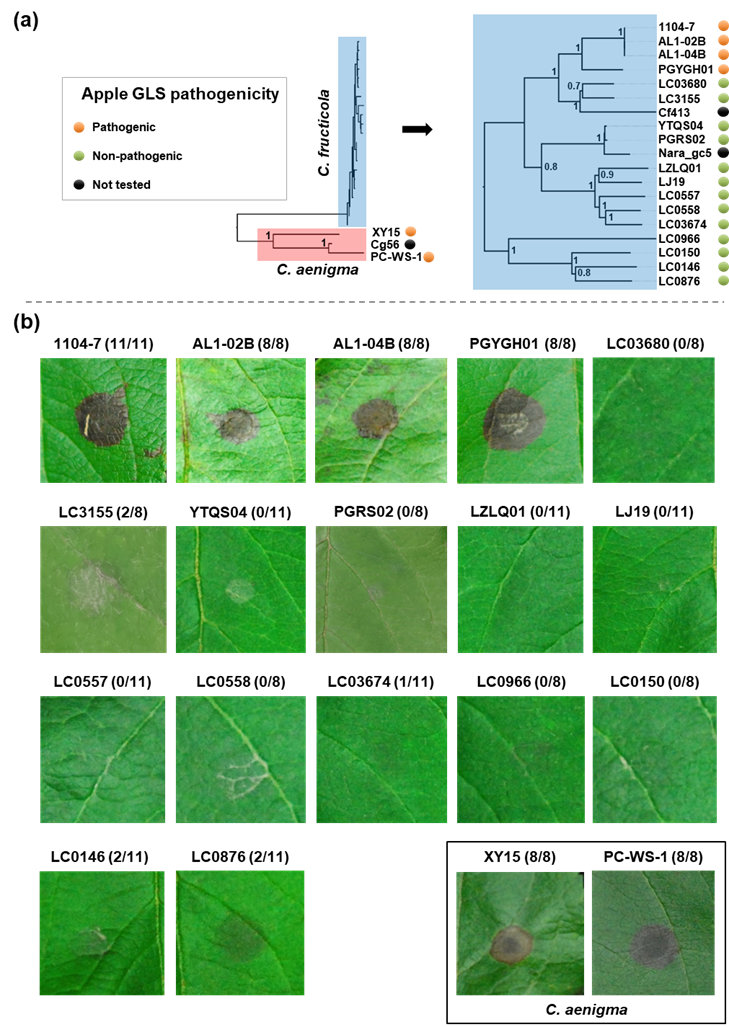


**Fig. S15** Isolates selected for genome resequencing and comparative analysis. (a) Neighbor-joining phylogenetic tree constructed with 21,741 parsimony informative SNP sites located within 1,326 single copy ascomycete BUSCO genes. Numbers at nodes indicate bootstrap values based on 1,000 replicates. (b) Representative photos showing disease development outcomes for indicated isolates upon artificial inoculation. Numbers within parenthesis indicate the number of leaves showing GLS symptom and the total inoculated leaves. Conidial suspensions (1 × 10^7^/mL) were drop-inoculated on the upper surface of healthy and detached apple leaves and the leaves were photographed at 4 dpi. More complete information of the isolates (host and geographic origin) is shown in Dataset S1.
