# Supplementary material for: Genome comparisons reveal accessory genes crucial for the evolution of apple Glomerella leaf spot pathogenicity in Colletotrichum fungi
Source: Mol Plant Pathol. 2024 Apr 15;25(4):e13454. doi: 10.1111/mpp.13454 (PMC11018114; doi:10.1111/mpp.13454)
Supplement: Supplementary file 20 — FIGURE S16. Genome‐wide DNA presence–absence polymorphism among Colletotrichum fructicola and C. aenigma isolates. Illumina or PacBio reads derived from 19 C. fructicola isolates and three C. aenigma isolates were mapped against the 1104‐7 reference genome, fraction of 1104‐7 DNA lacking reads coverage was calculated for each 10 kb window and used for heatmap generation. Tracks from outside to inside represent assembled scaffolds, histograms of gene density and repetitive element density and read coverage heatmaps. For heatmap tracks, grey (1–19) represents C. fructicola, whereas light blue (20–22) represents C. aenigma; note that light colour indicates a high fraction of DNA coverage. Colour of the track number indicates the outcome of Glomerella leaf spot (GLS) pathogenicity assays, brown indicates GLS pathogenic, green indicates non‐pathogenic and black indicates not assessed. Note the two regions on scaffold 1 showing trans‐species conservation among GLS pathogenic isolates (GLS‐R1 and GLS‐R2, highlighted in green). Links inside the tracks indicate repetitive DNA fragments (>10 kb, >90% identity) identified by self‐BlastN search, yellow ribbons link repetitive DNAs within GLS‐specific regions, whereas black ribbons link repetitive DNAs within the rest of the genome. Isolates for the heatmap tracks are as follows: 1, 1104‐7; 2, AL1‐02B; 3, AL1‐04B; 4, PGYGH01; 5, LC03680; 6, LC3155; 7, Cf413; 8, YTQS04; 9, PGRS02; 10, Nara_gc5; 11, LZLQ01; 12, LJ19; 13, LC0557; 14, LC0558; 15, LC03674; 16, LC0966; 17, LC0510; 18, LC0146; 19, LC0876; 20, XY15; 21, Cg56; 22, PC‐WS‐1. Information on isolate origin, phylogenetic position and GLS pathogenicity phenotype are detailed in Dataset S1 and Figure S15. [file MPP-25-e13454-s005.docx]

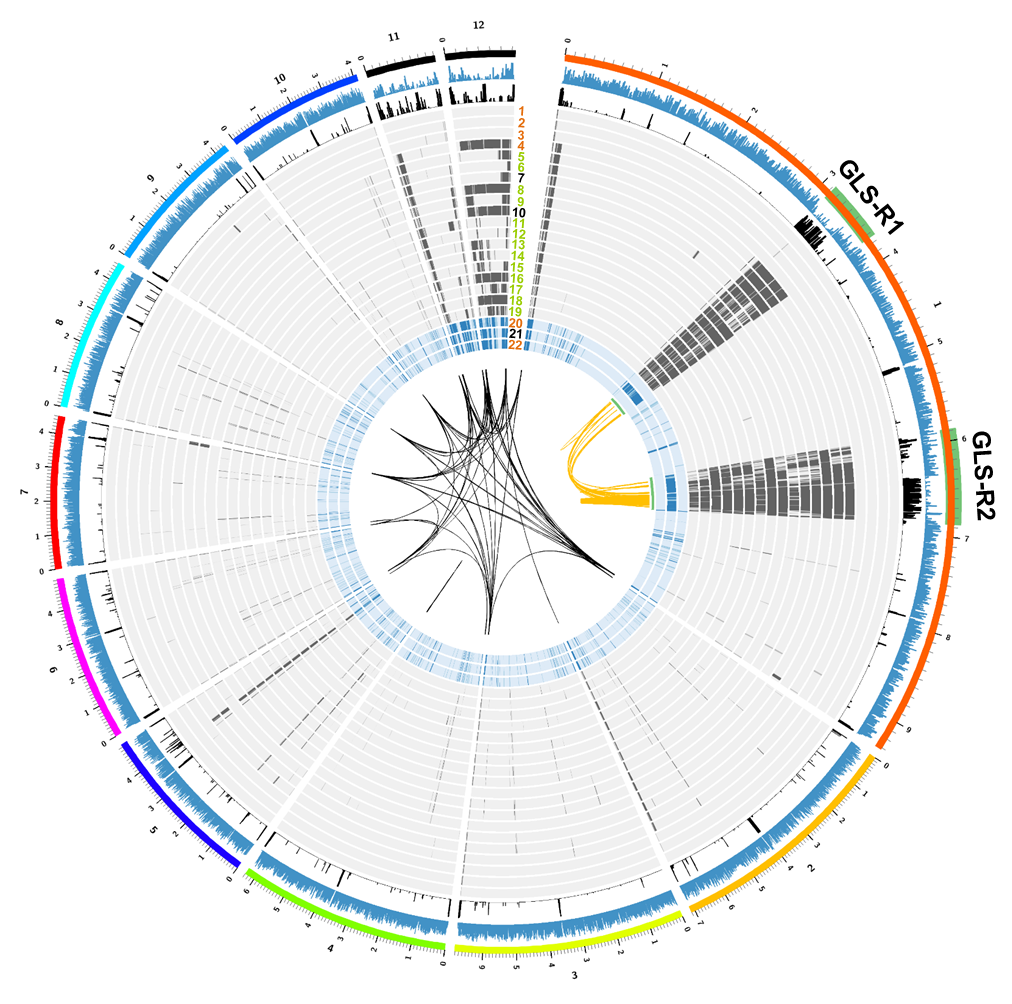


**Fig. S16** Genome wide DNA presence-absence polymorphism among *C. fructicola* and *C. aenigma* isolates. Illumina or PacBio reads derived from 19 *C. fructicola* isolates and 3 *C. aenigma* isolates were mapped against the 1104-7 reference genome, fraction of 1104-7 DNA lacking reads coverage was calculated for each 10 kb window and used for heatmap generation. Tracks from outside to inside represent assembled scaffolds, histograms of gene density and repetitive element density, and read coverage heatmaps. For heatmap tracks, grey (1 to 19) represents *C. fructicola* whereas light blue (20 to 22) represents *C. aenigma*, note that light color indicates a high fraction of DNA coverage. Color of the track number indicates the outcome of GLS pathogenicity assays, brown indicates GLS pathogenic, green indicates non-pathogenic, and black indicates not assessed. Note the two regions on scaffold 1 showing trans-species conservation among GLS pathogenic isolates (GLS-R1 and GLS-R2, highlighted in green). Links inside the tracks indicate repetitive DNA fragments (> 10kb, > 90% identity) identified by self-BlastN search, yellow ribbons link repetitive DNAs within GLS-specific regions whereas black ribbons link repetitive DNAs within the rest of the genome. Isolates for the heatmap tracks are as follows: 1, 1104-7; 2, AL1-02B; 3, AL1-04B; 4, PGYGH01; 5, LC03680; 6, LC3155; 7, Cf413; 8, YTQS04; 9, PGRS02; 10, Nara_gc5; 11, LZLQ01; 12, LJ19; 13, LC0557; 14, LC0558; 15, LC03674; 16, LC0966; 17, LC0510; 18, LC0146; 19, LC0876; 20, XY15; 21, Cg56; 22, PC-WS-1. Information on isolate origin, phylogenetic position, and GLS pathogenicity phenotype are detailed in Dataset S1 and Figure S15.
