# Supplementary material for: Genome comparisons reveal accessory genes crucial for the evolution of apple Glomerella leaf spot pathogenicity in Colletotrichum fungi
Source: Mol Plant Pathol. 2024 Apr 15;25(4):e13454. doi: 10.1111/mpp.13454 (PMC11018114; doi:10.1111/mpp.13454)
Supplement: Supplementary file 21 — FIGURE S17. Generation and verification of gene deletion mutants for GPCGs putatively regulating Glomerella leaf spot pathogenicity. (a) Schematic representation of the gene deletion strategy based on homologous recombination, primers used for mutant detection are indicated. (b) Partial gene deletion strategy for GPCG17, which is large in size. (c) PCR identification of gene deletion mutants based on three pairs of detection primers. W indicates wild‐type control, N indicates no‐template control, Arabic number indicates designation for gene deletion strain. [file MPP-25-e13454-s013.docx]

**
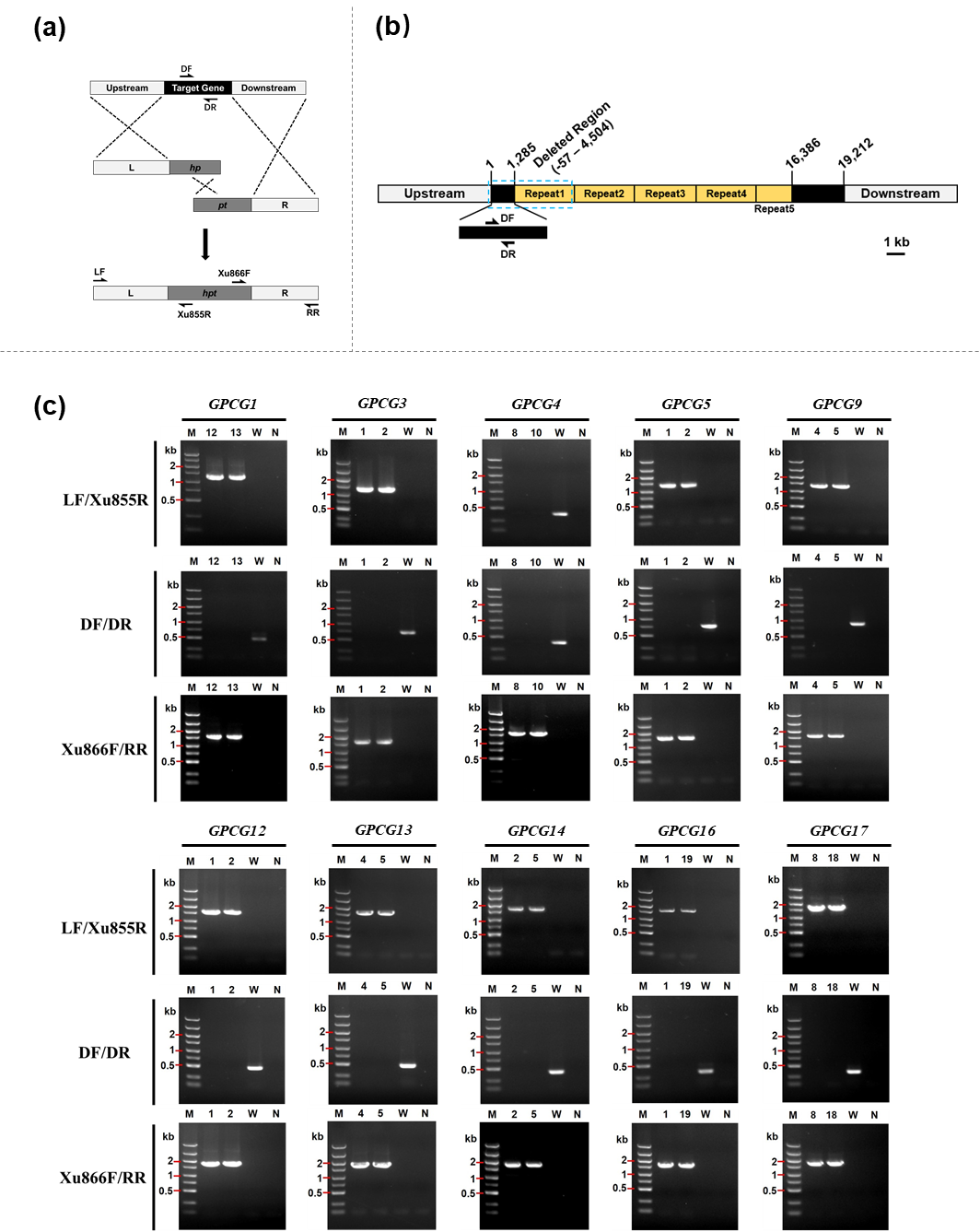
**

**Fig. S17** Generation and verification of gene deletion mutants for GPCGs putatively regulating GLS pathogenicity. (a) Schematic representation of the gene deletion strategy based on homologous recombination, primers used for mutant detection are indicated; (b) Partial gene deletion strategy for GPCG17 which is large in size; (c) PCR identification of gene deletion mutants based on three pairs of detection primers. W indicates Wild Type control and N indicates none-template control, arabic number indicates designation for gene deletion strain.
