# Supplementary material for: Genome comparisons reveal accessory genes crucial for the evolution of apple Glomerella leaf spot pathogenicity in Colletotrichum fungi
Source: Mol Plant Pathol. 2024 Apr 15;25(4):e13454. doi: 10.1111/mpp.13454 (PMC11018114; doi:10.1111/mpp.13454)
Supplement: Supplementary file 22 — FIGURE S18. Relative gene expression of GPCG1, GPCG16 and GPCG17 determined by reverse transcription–quantitative PCR. Fungal tissues included conidia (CON), in vitro‐grown mycelia (MYC, potato dextrose broth shake culture, 4 days), appressoria (APP) and infected apple fruit tissue sampled at 5 days after wounded inoculation (FRU) and infected apple leaves sampled at different time points (IL). Relative expression quantifications were calculated relative to CON. Error bar indicates standard deviation based on three independent technical replicates. [file MPP-25-e13454-s024.docx]

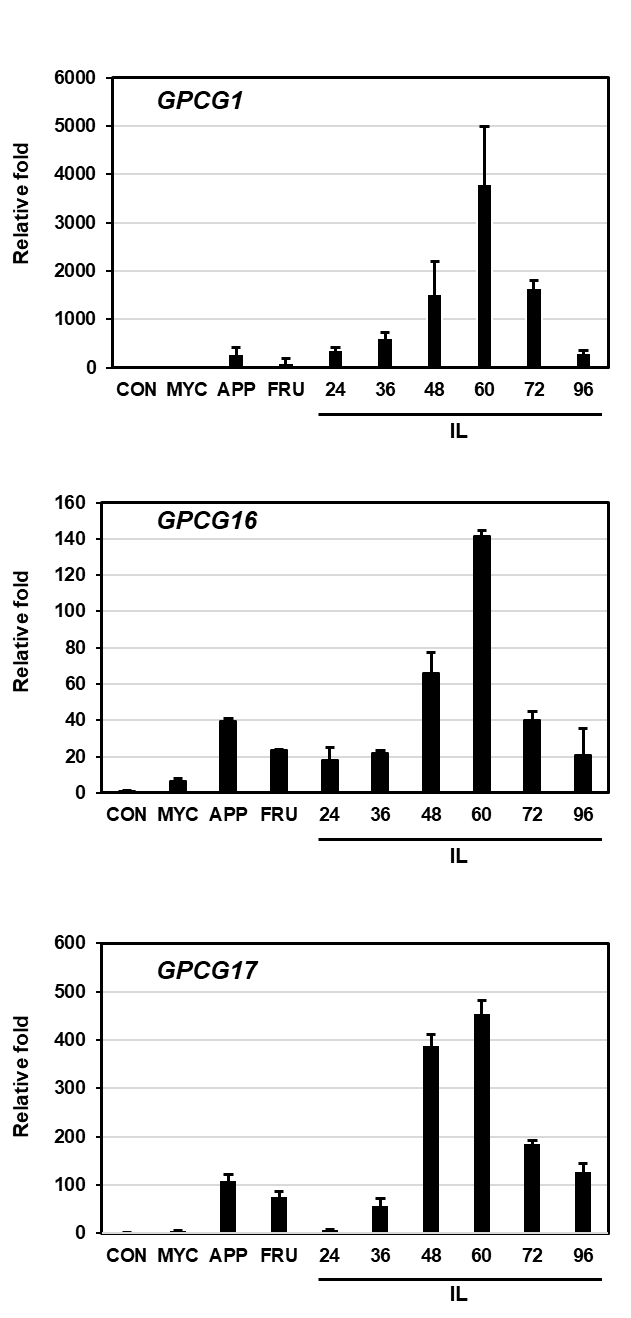


**Fig. S18** Relative gene expression of *GPCG1*, *GPCG16* and *GPCG17* determined by qRT-PCR. Fungal tissues included conidia (CON), *in vitro*-grown mycelia (MYC, PDB shake culture, 4 d), appressoria (APP), and infected apple fruit tissue sampled at five days post wounded inoculation (FRU), and infected apple leaves sampled at different time points (IL). Relative expression quantifications were calculated relative to CON. Error bar indicates standard deviation based on three independent technical replicates.
