# Supplementary material for: Genome comparisons reveal accessory genes crucial for the evolution of apple Glomerella leaf spot pathogenicity in Colletotrichum fungi
Source: Mol Plant Pathol. 2024 Apr 15;25(4):e13454. doi: 10.1111/mpp.13454 (PMC11018114; doi:10.1111/mpp.13454)
Supplement: Supplementary file 23 — FIGURE S19. Gene deletion effects of GPCG1, GPCG16 and GPCG17 on fungal mycelial growth, perithecial development and in vitro appressorium development. (a) Potato dextrose agar colony morphology at 5 days. (b) Perithecium development of different strains on oatmeal agar at 7 days, scale bar = 500 μm. (c) In vitro development of appressoria and infectious hyphae on cellophane at 24 h post‐inoculation, scale bar = 20 μm. APP, appressorium; CON, conidium; GT, germ tube; IH, infectious hyphae within cellophane. [file MPP-25-e13454-s019.docx]

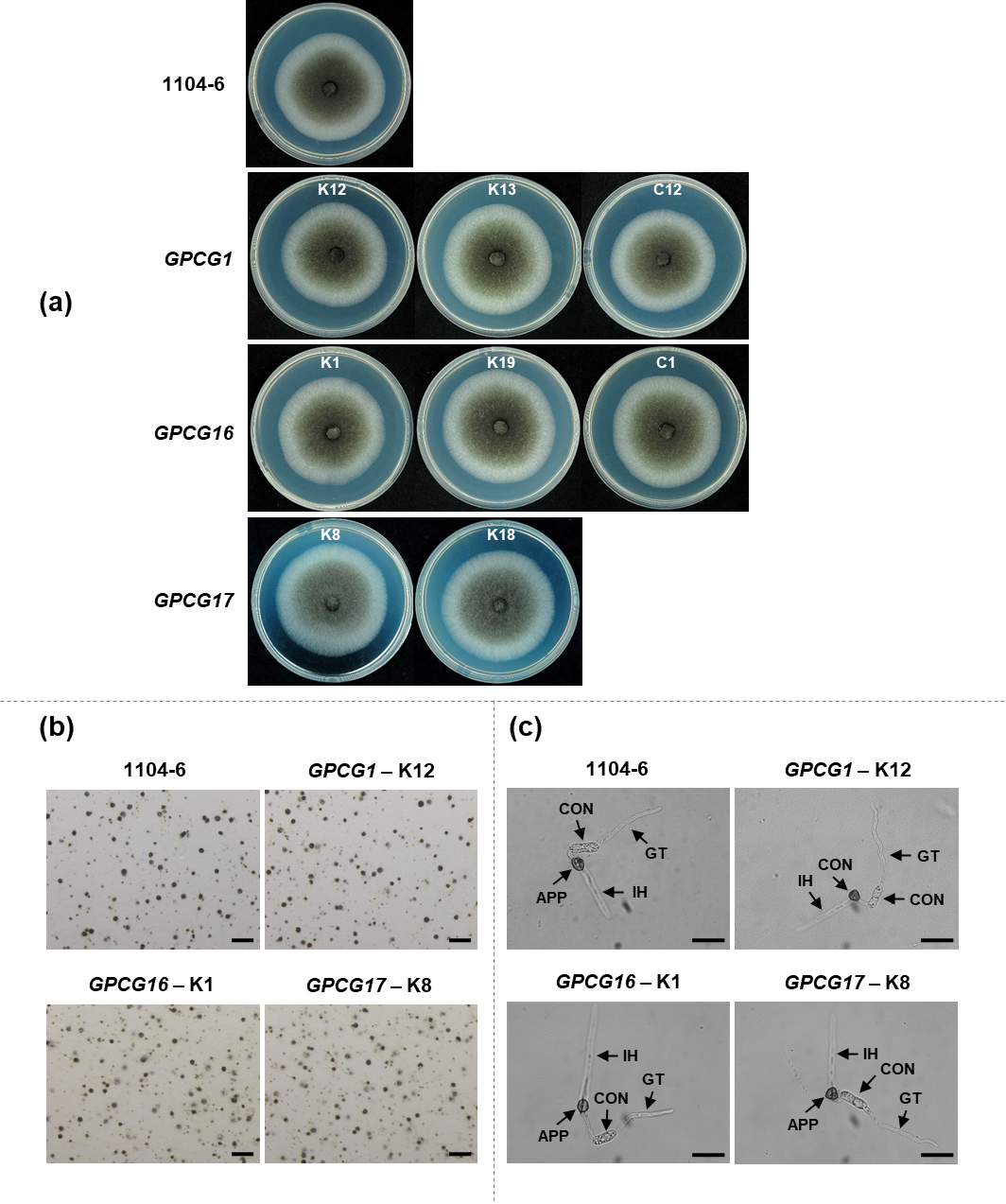


**Fig. S19** Gene deletion effects of *GPCG1*, *GPCG16* and *GPCG17* on fungal mycelial growth, perithecial development and in *vitro* appressorium development. (a) PDA colony morphology at 5 d; (b) Perithecium development of different strains on oatmeal agar at 7 d, scale bar = 500 μm; (c) In *vitro* development of appressoria and infectious hyphae on cellophane at 24 hpi, scale bar = 20 μm. CON: conidium; GT: germ tube; APP: appressorium; IH: infectious hyphae within cellophane.
