# Supplementary material for: Genome comparisons reveal accessory genes crucial for the evolution of apple Glomerella leaf spot pathogenicity in Colletotrichum fungi
Source: Mol Plant Pathol. 2024 Apr 15;25(4):e13454. doi: 10.1111/mpp.13454 (PMC11018114; doi:10.1111/mpp.13454)
Supplement: Supplementary file 24 — FIGURE S20. Histological visualization of fungal infectious hyphae development. Infected apple leaves were sampled at 96 h post‐inoculation, and subjected to tissue clearing, fixation and FITC‐WGA staining, and observed under fluorescent microscopy. Note the obvious mesophyll cell necrosis and infectious hyphae (IH) differentiation in the WT (1104‐6) infection, but not the mutant infections. Scale bar = 100 μm. [file MPP-25-e13454-s023.docx]

**
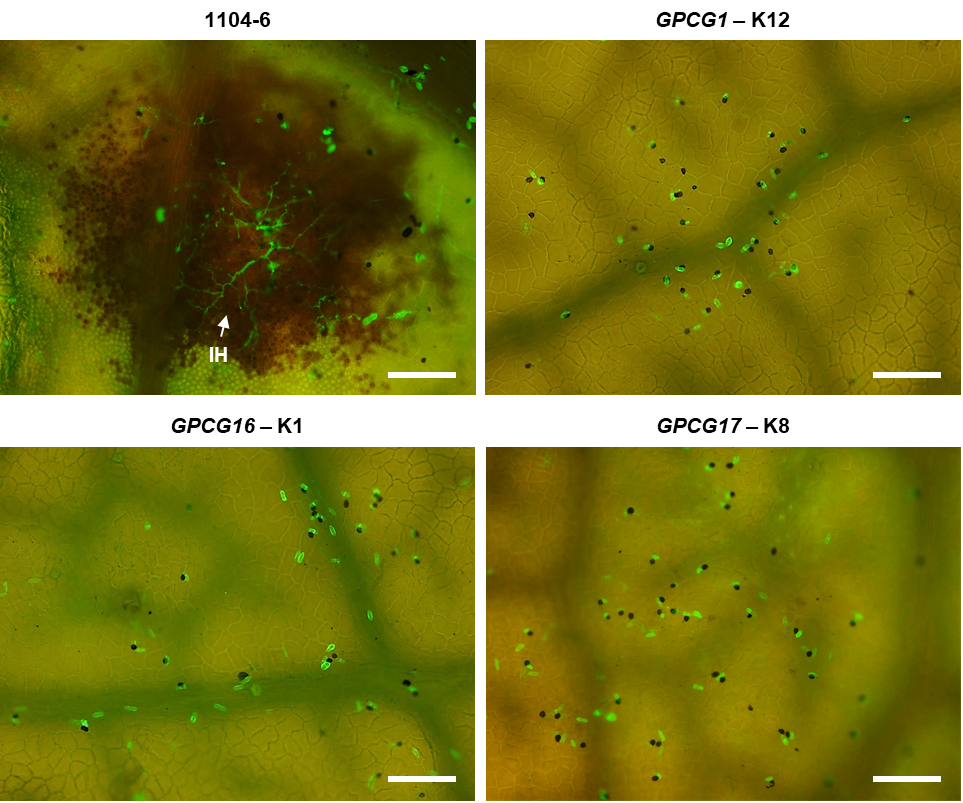
**

**Fig. S20** Histological visualization of fungal infectious hyphae development. Infected apple leaves were sampled at 96 hpi, and subjected to tissue clearing, fixation, and FITC-WGA staining, and observed under fluorescent microscopy. Note the obvious mesophyll cell necrosis and infectious hyphae (IH) differentiation in the WT (1104-6) infection, but not the mutant infections. Scale bar = 100 μm.
