# Supplementary material for: Genome comparisons reveal accessory genes crucial for the evolution of apple Glomerella leaf spot pathogenicity in Colletotrichum fungi
Source: Mol Plant Pathol. 2024 Apr 15;25(4):e13454. doi: 10.1111/mpp.13454 (PMC11018114; doi:10.1111/mpp.13454)
Supplement: Supplementary file 25 — FIGURE S21. Functional and evolutionary characteristics of GPCG1, GPCG16 and GPCG17 genes. (a) Schematic representation of the relative location of GPCG1, GPCG16 and GPCG17 on chromosome 1 and the domain structures of the corresponding encoded proteins. (b) Maximum‐likelihood phylogenetic trees constructed with GPCGs (red) and their best NCBI Blast hits in Colletotrichum (light blue) and non‐Colletotrichum (light green) species. For GPCG1 and GPCG16, full‐length proteins were used for tree construction. For GPCG17, sequences corresponding the third condensation domain were used for tree construction. Best amino acid substitution models for individual protein alignments were determined by Prottest, which were LG + I + G, JTT + I + G and JTT + I + G for GPCG1, GPCG16 and GPCG17 respectively. Phylogenetic trees are mid‐rooted and numbers at node indicate bootstrap value based on 1000 replicates, low‐supported values (<70) are in grey. [file MPP-25-e13454-s031.docx]

**
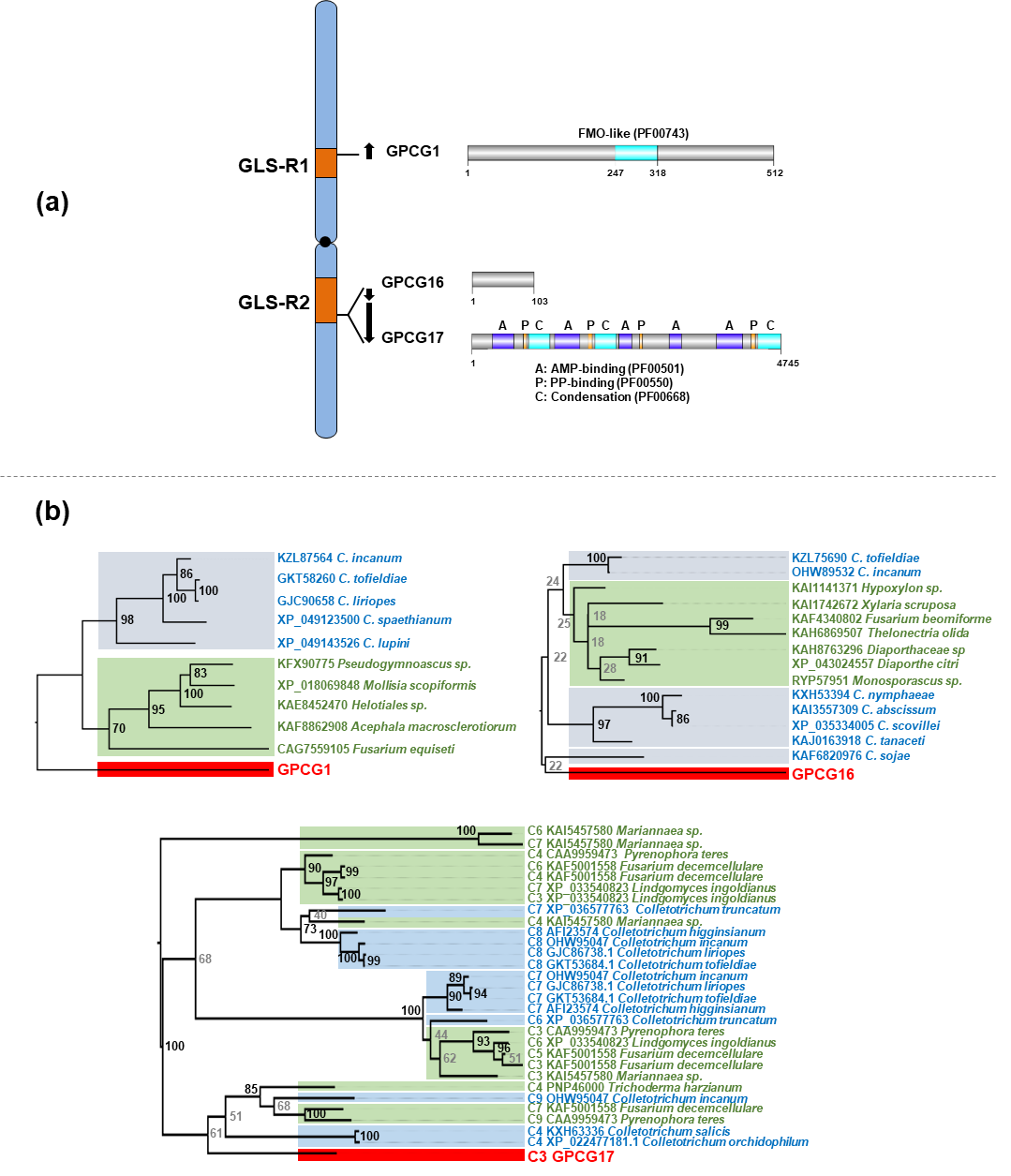
**

**Fig. S21** Functional and evolutionary characteristics of *GPCG1*, *GPCG16* and *GPCG17* genes. (a) Schematic representation of the relative location of *GPCG1*, *GPCG16* and *GPCG17* on chromosome 1 and the domain structures of the corresponding encoded proteins. (b) Maximum likelihood phylogenetic trees constructed with GPCGs (red) and their best NCBI BLAST hits in *Colletotrichum* (light blue) and non-*Colletotrichum* (light green) species. For GPCG1 and GPCG16, full-length proteins were used for tree construction. For GPCG17, sequences corresponding the third condensation domain were used for tree construction. Best amino acid substitution models for individual protein alignments were determined by Prottest, which were ‘LG+I+G’, ‘JTT+I+G’, and ‘JTT+I+G’ for GPCG1, GPCG16 and GPCG17 respectively. Phylogenetic trees are mid-rooted and numbers at node indicate bootstrap value based on 1,000 replicates, low-supported values (< 70) are in grey.
