# Supplementary material for: Genome comparisons reveal accessory genes crucial for the evolution of apple Glomerella leaf spot pathogenicity in Colletotrichum fungi
Source: Mol Plant Pathol. 2024 Apr 15;25(4):e13454. doi: 10.1111/mpp.13454 (PMC11018114; doi:10.1111/mpp.13454)
Supplement: Supplementary file 26 — FIGURE S22. Schematic representation of the GLS‐R1 and GLS‐R2 insertion events in 1104‐7. Note the 6‐bp direct repeats in 1104‐7 between the left and right synteny breakpoints for GLS‐R1 and GLS‐R2. For 1104‐7, IGV long‐read mapping outcome for reads derived from four Colletotrichum fructicola isolates are shown on the right. Synteny breakpoints are indicated as triangles. [file MPP-25-e13454-s003.docx]

**
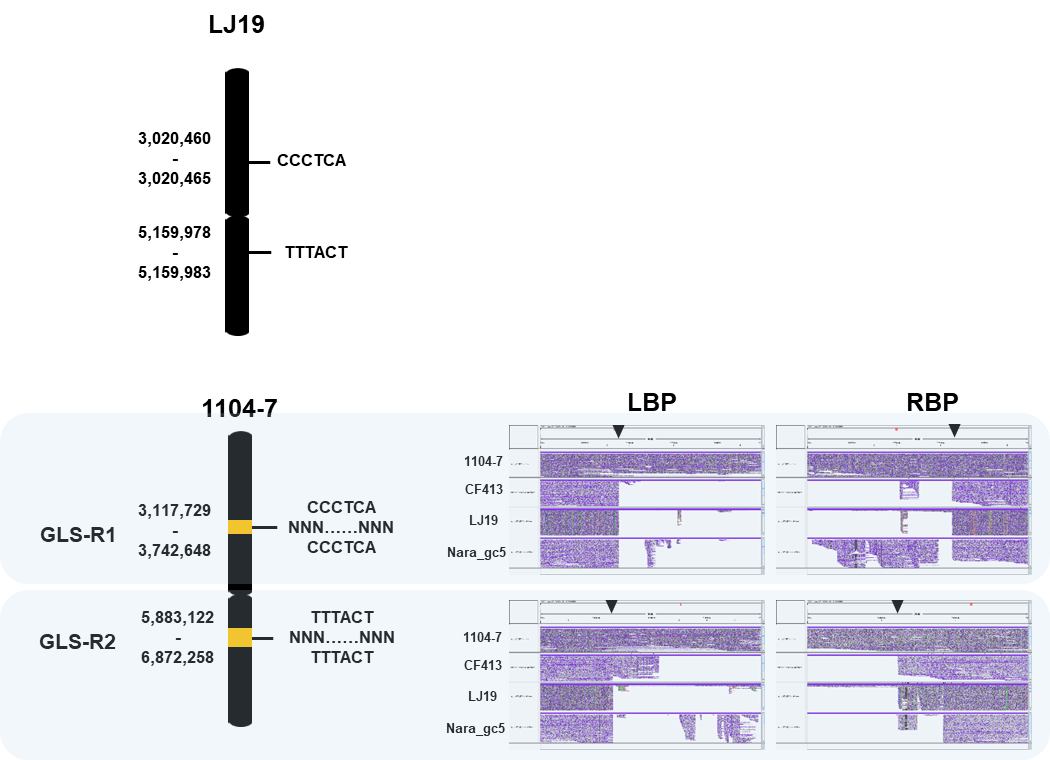
**

**Fig. S22** Schematic representation of the GLS-R1 and GLS-R2 insertion events in 1104-7. Note the 6-bp direct repeats in 1104-7 between the left and right synteny break points for GLS-R1 and GLS-R2. For 1104-7, IGV long read mapping outcome for reads derived from four *C. fructicola* isolates are shown on the right. Synteny break points are indicated as triangles.
