# Supplementary material for: Genome comparisons reveal accessory genes crucial for the evolution of apple Glomerella leaf spot pathogenicity in Colletotrichum fungi
Source: Mol Plant Pathol. 2024 Apr 15;25(4):e13454. doi: 10.1111/mpp.13454 (PMC11018114; doi:10.1111/mpp.13454)
Supplement: Supplementary file 27 — TABLE S1. Genome assembly statistics of Colletotrichum fructicola 1104‐7 and LJ19. [file MPP-25-e13454-s028.docx]

**Table S1 Genome assembly statistics of *Colletotrichum fructicola* 1104-7 and LJ19**

|  | **1104-7^a^** | **LJ19** |
| --- | --- | --- |
| Place of origin | Hebei, China | Shaanxi, China |
| Host | *Malus domestica* 'Gala' | *Capsicum annuum* |
| Glomerella leaf spot pathogenicity | Yes | No |
| Generated nanopore reads (Counts/Bases/N50 read length) | 357984/7.56Gb/27.87kb | 1261750/27.46Gb/28.97kb |
| Assembly software | NextDenovo | NextDenovo |
| Assembly size (Mb) | 58.55 | 56.69 |
| GC content (%) | 53.11 | 53.20 |
| Scaffold number | 12 | 12 |
| Scaffolds with telomeric repeats on one end | 4 | 5 |
| Scaffolds with telomeric repeats on both ends | 5 | 6 |
| Putative centromeres | 12 | 12 |
| BUSCO complete (%) | 98.8 | 98.9 |
| Predicted protein coding genes | 18,094 | 17,740 |

^a^A different version of 1104-7 genome assembly using the same set of nanopore reads data reported here but with different assembling pipeline was reported previously (Liang et al., 2020)

**Reference**

**Liang X, Cao M, Li S, Kong Y, Rollins JA, Zhang R, Sun G. 2020.** Highly contiguous genome resource of *Colletotrichum fructicola* generated using long-read sequencing. Molecular Plant-Microbe Interactions. 33: 790-793.
