# Supplementary material for: Genome comparisons reveal accessory genes crucial for the evolution of apple Glomerella leaf spot pathogenicity in Colletotrichum fungi
Source: Mol Plant Pathol. 2024 Apr 15;25(4):e13454. doi: 10.1111/mpp.13454 (PMC11018114; doi:10.1111/mpp.13454)
Supplement: Supplementary file 28 — TABLE S2. Presence of telomeric repeats at the scaffold ends. [file MPP-25-e13454-s007.docx]

**Table S2 Presence of telomeric repeats at the scaffold ends**

|  | **Telomere-5'** | **Telomere-3'** |
| --- | --- | --- |
| 1104-7-S1 | No/Yes (722 bp) **^a^** | No/Yes (352 bp) |
| 1104-7-S2 | Yes | Yes |
| 1104-7-S3 | No/Yes (26,469 bp) | No/Yes (22,895 bp) |
| 1104-7-S4 | No/Yes (11,981 bp) | No |
| 1104-7-S5 | No | Yes |
| 1104-7-S6 | No | No |
| 1104-7-S7 | No | Yes |
| 1104-7-S8 | No/Yes (146 bp) | Yes |
| 1104-7-S9 | Yes | Yes |
| 1104-7-S10 | Yes | Yes |
| 1104-7-S11 | Yes | Yes |
| 1104-7-S12 | Yes | Yes |
| LJ19-S1 | Yes | Yes |
| LJ19-S2 | Yes | No/Yes (34 bp) |
| LJ19-S3 | No | No |
| LJ19-S4 | Yes | No |
| LJ19-S5 | No | Yes |
| LJ19-S6 | Yes | Yes |
| LJ19-S7 | Yes | Yes |
| LJ19-S8 | Yes | Yes |
| LJ19-S9 | No/Yes (57 bp) | Yes |
| LJ19-S10 | Yes | No |
| LJ19-S11 | No/Yes (100 bp) | Yes |
| LJ19-S12 | Yes | Yes |

^a^For scaffold ends lacking telomeric repeats, self-corrected reads (produced by Canu) aligned to these regions were identified in IGV, and representative reads were manually checked for the presence of telomeric repeats. Slash indicates that reads at scaffold ends extend outwards to reach telomeric repeats, and the extended read lengths are indicated in parenthesis.
