# Supplementary material for: Genome comparisons reveal accessory genes crucial for the evolution of apple Glomerella leaf spot pathogenicity in Colletotrichum fungi
Source: Mol Plant Pathol. 2024 Apr 15;25(4):e13454. doi: 10.1111/mpp.13454 (PMC11018114; doi:10.1111/mpp.13454)
Supplement: Supplementary file 29 — TABLE S3. Annotation of isolate‐specific genomic rearrangement events on core chromosomes of the three Colletotrichum fructicola assemblies (CF413, LJ19, Nara_gc5) relative to the 1104‐7 reference. [file MPP-25-e13454-s008.docx]

**Table S3. Annotation of isolate-specific genomic rearrangement events on core chromosomes of the three *C. fructicola* assemblies (CF413, LJ19, Nara_gc5) relative to the 1104-7 reference**

| **Index** | **1104-7 Chromosome** | **1104-7 BP start (bp)** | **1104-7 BP end (bp)** | **1104-7 interval length (bp)** | **Rearranging strain, chromosome** | **BP-associated repeat element insertion in the rearranging strain** | **Note** |
| --- | --- | --- | --- | --- | --- | --- | --- |
| INV1 | S4 | 3,668,146 | 3,684,045 | 15,899 | LJ19, S4 | Yes, Tc1–Mariner TIR insertions at both BPs. | Both BPs are intergenic, the inversion region locates in a repeat-rich region. Tc1–Mariner TEs are inserted at the LJ19 BP sites in reverse direction. |
| INV2 | S6 | 63,6661 | 1,223,930 | 587,269 | CF413, S5; Nara_gc5, S10; LJ19, S6 | No | Both BPs are intragenic, locates in Cf1104nano2\|06137 (MFS transporter) and Cf1104nano2\|06304 (MFS transporter) respectively. The two genes are products of inversion-associated gene split. |
| INV3 | S7 | 4,091,800 | 4,110,925 | 19,125 | LJ19, S7 | Yes, Tad1 LINE insertions at both BPs. | Both BPs are intergenic. The locus is complex, except for invert TE elements at the LJ19 BP sites, LJ19 losses two DNA fragments (876 bp and 1,969 bp), and gains a TE element (3899 bp) neighboring the BP sites. |
| INV4 | S10 | 125,416 | 3,034,438 | 2,909,022 | LJ19, S10 | Yes, Tad1 LINE insertions at both BPs | Both BPs are intragenic, locates in Cf1104nano2\|02411 (hypothetical protein) and Cf1104nano2\|03341 (MFS transporter) respectively. LINE/Tad1 (CgT1) TEs are inserted at the LJ19 BP sites in reverse direction. The inversion is likely disrupting the functions of both genes. |
| INV5 | S8 | 637,001 | 731,098 | 94,097 | Nara_gc5, S6 | Yes, Gypsy LTR insertions at both BPs | Both BPs are intergenic. LTR-Gypsy TEs are inserted at the Nara_gc5 BP sites in reverse direction. |
| TRA1 | S1, S2 | 9,257,364 for S1, 5,378,517 for S2 | 9,257,365 for S1, and 5,378,518 for S2 | 1 | Nara_gc5, S5, S1, S5 | No | Both BPs are intragenic, locates in Cf1104nano2\|10323 (hypothetical protein) and Cf1104nano2\|05354 (hydrolase) respectively. The translocation is likely disrupting the functions of both genes. |
| TRA2 | S5 | 918,752 | 918,765 | 13 | Nara_gc5, S11, S8 | No | Both BPs are intergenic. |
| TRA3 | S6, S9 | 1,348,957 for S6, 2,696,734 for S9 | 1,349,377 for S6, 2,697,324 for S9 | 420 | Nara_gc5, S10, S3 | No | Both BPs are intergenic. |
