# Supplementary material for: Genome comparisons reveal accessory genes crucial for the evolution of apple Glomerella leaf spot pathogenicity in Colletotrichum fungi
Source: Mol Plant Pathol. 2024 Apr 15;25(4):e13454. doi: 10.1111/mpp.13454 (PMC11018114; doi:10.1111/mpp.13454)
Supplement: Supplementary file 30 — TABLE S4. Clusters of Colletotrichum fructicola secondary metabolite (SM) genes showing homology to fungal SM clusters producing known products. [file MPP-25-e13454-s018.docx]

**Table S4 Clusters of *C. fructicola* SM genes showing homology to fungal SM clusters producing known products**

| **Code** | **Location** | **Total genes** | **Number of matched genes** | **Protein sequence identities of matched genes (range/average)** | **Matched gene cluster** | **Related metabolite and main biological functions** | **Reference** |
| --- | --- | --- | --- | --- | --- | --- | --- |
| 1 | Scaffold 1, 1,260,752 – 1,292,018 | 9 | 9 | 31.4% - 62.2% / 50.9% | BGC0001541 | Cercosporin in diverse fungi, toxin, phytotoxic, assists plant infection | De Jonge et al., 2018 |
| 2 | Scaffold 1, 8,016,757– 8,052,378 | 7 | 7 | 27.1% - 71.7% / 61.3% | BGC0002235 | ACE1 in *Magnaporthe grisea*, cytochalasan-related, confers avirulence in Pi33 rice cultivars and may promote fungal early infection | Collemare et al., 2008 |
| 3 | Scaffold 2, 2,727,388– 2,743,348 | 5 | 4 | 66.3% - 75.7% / 69.5% | BGC0000012 | Alternapyrone in *Alternaria solani*, antimicrobial, antitumor, and cytotoxic activities | Fujii et al., 2005 |
| 4 | Scaffold 2, 4,592,900– 4,620,971 | 12 | 5 | 59.6% - 75.3% / 65.3% | BGC0002710 | metachelin C in *Metarhizium robertsii*, coprogen siderophore | Krasnoff et al., 2014 |
| 5 | Scaffold 3, 5,875,482– 5,907,765 | 9 | 6 | 40.2% - 65.6% / 55.2% | BGC0002093 | ilicicolin H in *Talaromyces variabilis*, broad spectrum antifungal activity | Singh et al., 2012 |
| 6 | Scaffold 4, 1,174,379– 1,198,906 | 6 | 6 | 47.7% - 71.9% / 59.7% | BGC0001545 | chrysogine in *Fusarium graminearum*, yellow pigment, lacks antimicrobial or anticancer activity | Wollenberg et al., 2017 |
| 7 | Scaffold 4, 4,132,431 – 4,151,619 | 7 | 7 | 67.8% - 74.6% / 72.3% | BGC0001609 | Gliovirin in *Trichoderma virens*, anti-oomycete and anti-cancer activity | Sherkhane et al., 2017 |
| 8 | Scaffold 5, 288,389 – 323,997 | 9 | 7 | 44.1% - 55.1% / 51.6% | BGC0000304 | Apicidin in *Fusarium incarnatum*, potent histone deacetylase inhibitor | Jin et al., 2010 |
| 9 | Scaffold 5, 1,668,172 – 1,692,733 | 6 | 6 | 30.1% - 51.5% / 39.1% | BGC0002180 | Asperlin in *Aspergillus nidulans*, antifungal and anti-inflammatory activities | Grau et al., 2018 |
| 10 | Scaffold 7, 342,468 – 360,914 | 7 | 5 | 49.4% - 66.4% / 58.4% | BGC0002165 | Betaenone C in *Parastagonospora nodorum*, phytotoxicity | Li et al., 2019 |

**References**

**Collemare J, Pianfetti M, Houlle AE, Morin D, Camborde L, Gagey MJ, Barbisan C, Fudal I, Lebrun MH, Böhnert HU. 2008**. *Magnaporthe grisea* avirulence gene ACE1 belongs to an infection-specific gene cluster involved in secondary metabolism. New Phytologist. 179: 196-208.

**De Jonge R, Ebert MK, Huitt-Roehl CR, Pal P, Suttle JC, Spanner RE, Neubauer JD, Jurick WM, Stott KA, Secor GA, Thomma B, Van De Peer Y, Townsend CA, Bolton MD. 2018.** Gene cluster conservation provides insight into cercosporin biosynthesis and extends production to the genus *Colletotrichum*. Proceedings of the National Academy of Sciences. 115: E5459-e5466.

**Fujii I, Yoshida N, Shimomaki S, Oikawa H, Ebizuka Y. 2005**. An iterative type I polyketide synthase PKSN catalyzes synthesis of the decaketide alternapyrone with regio-specific octa-methylation. Chemical Biology. 12: 1301-1309.

**Grau MF, Entwistle R, Chiang YM, Ahuja M, Oakley CE, Akashi T, Wang CCC, Todd RB, Oakley BR. 2018**. Hybrid transcription factor engineering activates the silent secondary metabolite gene cluster for (+)-asperlin in *Aspergillus nidulans*. ACS Chemical Biology. 13: 3193-3205.

**Jin JM, Lee S, Lee J, Baek SR, Kim JC, Yun SH, Park SY, Kang S, Lee YW. 2010.** Functional characterization and manipulation of the apicidin biosynthetic pathway in *Fusarium semitectum*. Molecular Microbiology. 76: 456-466.

**Krasnoff SB, Keresztes I, Donzelli BG, Gibson DM. 2014.** Metachelins, mannosylated and N-oxidized coprogen-type siderophores from *Metarhizium robertsii*. Journal of Natural Products. 77: 1685-1692.

**Li H, Hu J, Wei H, Solomon PS, Stubbs KA, Chooi Y-H. 2019.** Biosynthesis of a tricyclo[6.2.2.02,7]dodecane system by a berberine bridge enzyme-like aldolase. Chemistry – A European Journal. 25: 15062-15066.

**Sherkhane PD, Bansal R, Banerjee K, Chatterjee S, Oulkar D, Jain P, Rosenfelder L, Elgavish S, Horwitz BA, Mukherjee PK. 2017.** Genomics-driven discovery of the gliovirin biosynthesis gene cluster in the plant beneficial fungus *Trichoderma virens*. Chemistry Select. 2: 3347-3352.

**Singh SB, Liu W, Li X, Chen T, Shafiee A, Card D, Abruzzo G, Flattery A, Gill C, Thompson JR, Rosenbach M, Dreikorn S, Hornak V, Meinz M, Kurtz M, Kelly R, Onishi JC. 2012.** Antifungal spectrum, in vivo efficacy, and structure-activity relationship of ilicicolin h. ACS Medicinal Chemistry Letters. 3: 814-817.

**Wollenberg RD, Saei W, Westphal KR, Klitgaard CS, Nielsen KL, Lysøe E, Gardiner DM, Wimmer R, Sondergaard TE, Sørensen JL. 2017.** Journal of Natural Products. 80: 2131-2135.
