# Supplementary material for: Genome comparisons reveal accessory genes crucial for the evolution of apple Glomerella leaf spot pathogenicity in Colletotrichum fungi
Source: Mol Plant Pathol. 2024 Apr 15;25(4):e13454. doi: 10.1111/mpp.13454 (PMC11018114; doi:10.1111/mpp.13454)
Supplement: Supplementary file 31 — TABLE S5. Summary statistics of genome sequencing and reads mapping outcome for Colletotrichum fructicola and C. aenigma isolates against the 1104‐7 reference genome. [file MPP-25-e13454-s030.docx]

**Table S5. Summary statistics of genome sequencing and reads mapping outcome for *C. fructicola* and *C. aenigma* isolates against the 1104-7 reference genome.**

| **Strain ID^1^** | **Reads Type** | **Library Average Insert Size (bp)** | **Total Reads** | **Total Length (Mb)** | **Mapped Reads** | **Average Reads Depth** |
| --- | --- | --- | --- | --- | --- | --- |
| 1104-7 | Illumina | 100 | 59,476,600 | 6,006 | 57,296,525 | 97.9 |
| Al1-02B | Illumina | 150 | 8,288,516 | 1,240 | 8,071,846 | 20.7 |
| Al1-04B | Illumina | 150 | 7,363,248 | 1,102 | 7,203,983 | 18.5 |
| PGYGH01 | Illumina | 150 | 83,624,884 | 12,542 | 80,164,480 | 205.4 |
| PGRS02 | Illumina | 150 | 57,047,214 | 8,556 | 52,778,702 | 135.2 |
| YTQS04 | Illumina | 150 | 46,491,996 | 6,974 | 43,506,417 | 111.5 |
| MHTWG02 | Illumina | 150 | 38,281,918 | 5,742 | 36,428,346 | 93.3 |
| LZLQ01 | Illumina | 150 | 56,299,762 | 8,444 | 53,927,686 | 138.2 |
| LJMX19 | Illumina | 150 | 62,333,878 | 9,350 | 58,630,151 | 150.2 |
| XY15 | Illumina | 150 | 60,800,896 | 7,576 | 44,832,763 | 114.9 |
| PC-WS-1 | Illumina | 150 | 46,456,380 | 6,968 | 33,389,218 | 88.5 |
| LC0557 | Illumina | 150 | 56,459,922 | 8,468 | 53,171,750 | 136.2 |
| LC0558 | Illumina | 150 | 54,948,708 | 8,242 | 52,731,269 | 135.1 |
| LC0876 | Illumina | 150 | 50,902,828 | 7,636 | 48,637,347 | 124.6 |
| LC0966 | Illumina | 150 | 56,249,098 | 8,438 | 53,291,686 | 136.5 |
| LC0146 | Illumina | 150 | 62,474,668 | 9,372 | 59,640,549 | 152.8 |
| LC0150 | Illumina | 150 | 55,957,330 | 8,394 | 53,653,526 | 137.5 |
| LC3674 | Illumina | 150 | 56,061,898 | 8,410 | 53,139,211 | 136.1 |
| LC3680 | Illumina | 150 | 54,802,962 | 8,220 | 53,065,829 | 136 |
| LC0033 | Illumina | 150 | 61,889,954 | 9,284 | 58,391,242 | 149.6 |
| LC3155 | Illumina | 150 | 57,553,962 | 8,634 | 55,588,231 | 142.4 |
| Cg56 | Illumina | 100 | 43,095,028 | 4,354 | 27,722,246 | 71 |
| Cf413 | Pacbio | / | 632,784 | 10,384 | 525,952 | 147.4 |
| Nara_gc5 | Pacbio | / | 669,270 | 11,108 | 545,550 | 154.6 |
