# Supplementary material for: Genome comparisons reveal accessory genes crucial for the evolution of apple Glomerella leaf spot pathogenicity in Colletotrichum fungi
Source: Mol Plant Pathol. 2024 Apr 15;25(4):e13454. doi: 10.1111/mpp.13454 (PMC11018114; doi:10.1111/mpp.13454)
Supplement: Supplementary file 32 — TABLE S6. PFAM functional enrichment of the 208 variable genes located within GLS‐R1 and GLS‐R2 regions. [file MPP-25-e13454-s014.docx]

**Table S6. PFAM functional enrichment of the 208 variable genes located within GLS-R1 and GLS-R2 regions**

| **PFAM ID** | **PFAM annotation** | **Gene number** | **Fold enrichment** | **P-value (Hypergeometric test)** | **Q-value (Storey-Tibshirani method)** |
| --- | --- | --- | --- | --- | --- |
| PF07727 | Reverse transcriptase (RNA-dependent DNA polymerase) | 5 | 101 | 4.31E-10 | 1.18E-05 |
| PF13374 | Tetratricopeptide repeat | 4 | 34 | 4.84E-06 | 0.067 |
| PF01231 | Indoleamine 2,3-dioxygenase | 3 | 54 | 1.85E-05 | 0.17 |
| PF13424 | Tetratricopeptide repeat | 3 | 32 | 9.76E-05 | 0.62 |
| PF16187 | Middle or third domain of peptidase_M16 | 2 | 108 | 0.00011 | 0.62 |
| PF12340 | Protein of unknown function (DUF3638) | 2 | 81 | 0.00022 | 0.88 |
| PF12359 | Protein of unknown function (DUF3645) | 2 | 81 | 0.00022 | 0.88 |
| PF10373 | Est1 DNA/RNA binding domain | 2 | 64 | 0.00037 | 1 |
| PF00291 | Pyridoxal-phosphate dependent enzyme | 3 | 19 | 0.00047 | 1 |
