# Supplementary material for: Genome comparisons reveal accessory genes crucial for the evolution of apple Glomerella leaf spot pathogenicity in Colletotrichum fungi
Source: Mol Plant Pathol. 2024 Apr 15;25(4):e13454. doi: 10.1111/mpp.13454 (PMC11018114; doi:10.1111/mpp.13454)
Supplement: Supplementary file 33 — TABLE S7. PFAM functional enrichment of the 76 GLS‐specific genes located within GLS‐R1 and GLS‐R2 regions. [file MPP-25-e13454-s034.docx]

**Table S7. PFAM functional enrichment of the 76 GLS-specific genes located within GLS-R1 and GLS-R2 regions**

| **PFAM ID** | **PFAM annotation** | **Gene number** | **Fold enrichment** | **P-value (Hypergeometric test)** | **Q-value (Storey-Tibshirani method)** |
| --- | --- | --- | --- | --- | --- |
| PF00501 | AMP-binding enzyme | 4 | 28 | 1.88E-05 | 0.26 |
| PF00201 | UDP-glucoronosyl and UDP-glucosyl transferase | 3 | 57 | 1.81E-05 | 0.26 |
| PF13193 | AMP-binding enzyme C-terminal domain | 3 | 43 | 4.24E-05 | 0.39 |
| PF00425 | chorismate binding enzyme | 2 | 140 | 8.17E-05 | 0.56 |
| PF00078 | Reverse transcriptase (RNA-dependent DNA polymerase) | 2 | 84 | 0.00024 | 1 |
