# Supplementary material for: Genome comparisons reveal accessory genes crucial for the evolution of apple Glomerella leaf spot pathogenicity in Colletotrichum fungi
Source: Mol Plant Pathol. 2024 Apr 15;25(4):e13454. doi: 10.1111/mpp.13454 (PMC11018114; doi:10.1111/mpp.13454)
Supplement: Supplementary file 34 — TABLE S8. PFAM functional enrichment of the 49 GLS‐associated genes located within GLS‐R1 and GLS‐R2 regions. [file MPP-25-e13454-s017.docx]

**Table S8. PFAM functional enrichment of the 49 GLS-associated genes located within GLS-R1 and GLS-R2 regions**

| **PFAM ID** | **PFAM annotation** | **Gene number** | **Fold enrichment** | **P-value (Hypergeometric test)** | **Q-value (Storey-Tibshirani method)** |
| --- | --- | --- | --- | --- | --- |
| PF00425 | chorismate binding enzyme | 2 | 189 | 4.43E-05 | 1 |
| PF00078 | Reverse transcriptase (RNA-dependent DNA polymerase) | 2 | 113 | 0.00013 | 1 |
| PF13424 | Tetratricopeptide repeat | 2 | 76 | 0.00031 | 1 |
| PF17100 | N-terminal domain of NWD NACHT-NTPase | 2 | 71 | 0.00035 | 1 |
| PF01408 | Oxidoreductase family | 2 | 33 | 0.0016 | 1 |
